# Novel Phthalic-Based Anticancer Tyrosine Kinase Inhibitors: Design, Synthesis and Biological Activity

**DOI:** 10.3390/cimb45030117

**Published:** 2023-02-22

**Authors:** Elena Kalinichenko, Aliaksandr Faryna, Tatyana Bozhok, Anna Golyakovich, Alesya Panibrat

**Affiliations:** Institute of Bioorganic Chemistry, National Academy of Sciences of Belarus, 5/2 Academician V.F. Kuprevich Street, BY-220141 Minsk, Belarus

**Keywords:** terephthalic and isophthalic derivatives, anticancer activity, protein kinase inhibitors, VEGFR, cellular apoptosis, ROS, MM-PBSA/MM-GBSA, molecular docking

## Abstract

In this work, fragments of isophthalic and terephthalic acids are proposed as a structural scaffold to develop potential inhibitors of protein kinases. Novel isophthalic and terephthalic acid derivatives were designed as type-2 protein kinase inhibitors, synthesized and subjected to physicochemical characterization. The screening of their cytotoxic actions against a panel of cell lines derived from different types of tumors (liver, renal, breast and lung carcinomas, as well as chronic myelogenous and promyelocytic leukemia) and normal human B lymphocyte, for the sake of comparison, was performed. Compound **5** showed the highest inhibitory activity against four cancer cell lines, K562, HL-60, MCF-7 and HepG2 (IC_50_ = 3.42, 7.04, 4.91 and 8.84 µM, respectively). Isophthalic derivative **9** revealed a high potency against EGFR and HER2, at the levels of 90% and 64%, respectively, being comparable to lapatinib at 10 µM. In general, tumor cell cultures were more sensitive to isophthalic acid derivatives than to terephthalic acid ones. In cell cycle studies, isophthalic analogue **5** showed a pronounced dose-dependent effect, and with the increase in its concentration up to 10.0 µM, the number of living cells decreased to 38.66%, while necrosis reached 16.38%. The considered isophthalic compounds had a similar docking performance to that of sorafenib against the VEGFR-2 (PDB id: 4asd, 3wze). The correct binding of compounds **11** and **14** with VEGFR-2 was validated using MD simulations and MM-GPSA calculations.

## 1. Introduction

Targeted therapy with protein kinase inhibitors (PKIs) has significantly improved the treatment results of many cancers. PKI development was greatly enhanced by the success of imatinib for the treatment of chronic myelogenous leukemia (CML), which was approved by the FDA in 2001 [1]. Imatinib inhibits the specific CML cell protein, Bcr-Abl tyrosine kinase. The high and uncontrolled phosphorylating activity of this protein disturbs cell signaling and promotes the fast growth of cancer tissue.

It was later discovered that the dysregulation of protein kinase function could be seen in many other cancer types and that the blocking of these proteins reduced the development of a disease much more effectively than classic chemotherapy, generally without major side effects [2]. Since imatinib first showed its effectiveness, the number of approved PKIs has been constantly growing. To date, approximately 70 PKIs have been approved, targeting approximately 20 different classes of protein kinases [3,4]. Moreover, Bcr-Abl, the largest group of approved drugs, comprises the inhibitors of various growth factor receptors, including EGFR, VEGFR and PDGFR [5,6].

The modulation of selectivity is one of the most important aspects of the development of new PKIs. Since the treatment protocol for PKIs assumes that a patient needs to take the drug for a very long period of time, cancer cells have the opportunity to develop resistance to an inhibitor. The resistance mechanisms can cause major changes in the kinase overexpression profiles of cancer cells, which are often revealed in the form of mutations of the primary target of an inhibitor [7]. In particular, in the case of CML, second- and third-generation Bcr-Abl inhibitors (nilotinib, dasatinib, bosutinib), in addition to having a higher affinity for the main target, are also active against most mutant forms of this enzyme, although the T315I mutation is still a problem [8].

It is worth noting that there is a high level of structural similarity between protein kinases, as all of them accept ATP as a substrate [9]. This, in most cases, results in the non-specificity of a particular inhibitor for its main target. For example, in addition to Bcr-Abl, imatinib is able to inhibit c-Kit and PDGFR kinases, which also makes it possible to use this substance for the treatment of gastrointestinal stromal tumors (GIST) [10]. Lapatinib very specifically inhibits only two kinases: EGFR and the related Her2. This determines the effectiveness of lapatinib for the treatment of Her2-positive breast cancer [11,12]. Sunitinib, on the contrary, has an extremely wide spectrum of anti-kinase activities. It inhibits VEGFR, PDGFR, Kit and Ret, as well as many other protein kinases, and is used to treat GIST, RCC and pancreatic cancer [11,13]. Sorafenib has a similar selectivity profile to sunitinib, though it is not as broad, but inhibits Raf kinase, which enables sorafenib to treat not only RCC but also liver cancer and melanoma [11,14,15]. In the case of RCC, sunitinib has more pronounced side effects compared to sorafenib, which, to some extent, can be explained by differences between the kinase inhibition profiles of these two compounds [16]. In addition, Atg5-deficient DU145 prostate cancer cells have been shown to undergo caspase-independent autophagic cell death in response to sorafenib [17,18,19]. 

Crizotinib was originally developed as a met-kinase inhibitor. However, it was later discovered that the efficacy of crizotinib for the treatment of NSCLC can be attributed to its ability to inhibit alk-kinase [20], which likely contributed to the further development of many other alk inhibitors.

The problem of the selectivity of PKIs becomes more complicated when taking into account the fact that the profile of kinase hyperactivity significantly depends on the subtype of a particular cancer, the stage of the disease, and the patient’s specificity. These factors, combined, may affect the choice of the optimal inhibitor for the initial and subsequent treatment. For example, when considering treatment options for CML in cases of resistance to imatinib, one should focus on the empirically observed Bcr-Abl mutants, since the inhibitory activities of second-line drugs are different in relation to specific mutations [21,22]. At the same time, a fairly large number of inhibitors have been approved for the treatment of RCC. All these drugs bind to VEGFR as the main target, but they all have different activities against other protein kinases [23]. As a result, personalized medicine has the potential to be an extremely effective tool for selecting the best first-line and second-line therapies [24,25,26].

Considering how greatly the selectivity of an inhibitor affects its pharmacological properties, it is important to note that the obtained experimental data indicate that a significant proportion of the approved drugs are, in fact, not competitors of ATP as they bind to the biologically inactive conformations of their targets and are thus classified as type-2 PKIs [5]. The binding region of these inactive conformations is the ATP pocket, which is extended and modified to varying degrees, of biologically active conformations. This conformational flexibility of protein kinases is certainly a major tool for modulating PKI selectivity, as it allows inhibitors to more completely differentiate their targets in structural terms, a fact which is supported by in vitro profiling data [27]. For example, the high selectivity of lapatinib can be explained by the fact that it binds to a rather specific egfr conformation, which is a kind of an intermediate state between the kinase conformations that are typical of type-1 (bosutinib, dasatinib) and type-2 (sorafenib, imatinib) inhibitors [28].

It should also be noted that the size of the human kinome is approximately 500 kinases, with most protein kinases not being identified as drug targets. Additionally, if we also consider the recent advances in the use of protein kinase inhibitors for the treatment of non-cancer diseases [29], it becomes obvious that the search for novel chemical compounds that can inhibit protein kinases with a given selectivity is an urgent area of scientific research.

This work continues our previous studies on pharmacophore modeling and synthesis and the biological activities of potential type-2 PKIs. In our previous studies, based on the results of docking and molecular dynamics, we used the 4-methylbenzamide structural fragment as a basis for designing new chemical compounds that had pharmacophore similarity to imatinib, sorafenib and other known inhibitors [30]. These 4-methylbenzamide derivatives were able to inhibit protein kinases and block the growth of tumor cells in vitro [31,32].

In this work, fragments of isophthalic and terephthalic acids were proposed as a structural scaffold to develop potential inhibitors of protein kinases.

## 2. Materials and Methods

### 2.1. Chemistry

Organic solvents were purified and dried by standard methods before usage in the synthesis. Analytical thin-layer chromatography (TLC) was performed on aluminum-backed sheets on plates with Silica gel 60 F254 (Merck KGaA, Darmstadt, Germany). The following solvent systems were used: CHCl_3_: MeOH, 7:1, and *v*/*v* or EtOAc, isocratic. Preparative column chromatography was performed on Silica gel Merck 60, 70–230 mesh (Merck KGaA, Darmstadt, Germany). NMR spectra were recorded on a Bruker Avance 500 MHz spectrometer (Bruker BioSpin GmbH, Rheinstetten, Germany) at 500 MHz (^1^H), 125 MHz (^13^C) and 470 MHz (^19^F). Multiplicities are abbreviated as follows: s, singlet; d, doublet; t, triplet; q, quartet; and m, multiplet. High-resolution mass spectra (HRMS) were recorded on an Agilent 1290 Accurate-Mass 6500 Series Q-TOF (Agilent Technologies, Santa Clara, CA, USA) using ESI (electrospray ionization). Melting points (m.p.) were determined using an electrically heated melting point apparatus and were left uncorrected. The purities of the compounds were greater than 95%, as determined by reversed-phase HPLC.

#### 2.1.1. Synthesis of Isophthaloyl and Terephthaloyl Dichlorides **3** and **4**

To a suspension of isophthalic acid (**1**) or terephthalic acid (**2**) (one equivalent) and N,N-dimethylformamide (DMF, 0.1 equivalent) in tetrahydrofuran (THF) cooled to 0 °C, we added (COCl)_2_ (4 equivalents) dropwise. The reaction mixture was stirred at room temperature for 3 h. The progress was monitored by TLC. Upon completion of the reaction, the solvent was evaporated under reduced pressure. The residue was washed with CHCl_3_ and the solvent was evaporated under reduced pressure. The resulting compound **3** or **4** was immediately introduced to a further reaction.

#### 2.1.2. General Method for the Synthesis of Compounds **5**–**28**

To a solution of terephthaloyl dichloride (**2**) (1.1 equivalent) in THF cooled to 0 °C, we added a solution of amine (**R_1_H** = **a**/**b**/**c**) (one equivalent) and Et_3_N (one equivalent) in THF dropwise under nitrogen. The reaction mixture was stirred at room temperature. The progress was monitored by TLC. Upon full conversion of the starting amine, a solution of another amine (**R_2_H** = **b**/**c**/**e**/**g**/**h**) (one equivalent) and Et_3_N (one equivalent) in THF was added dropwise under nitrogen. The reaction mixture was stirred at room temperature. The progress was monitored by TLC. Upon completion of the reaction, water was added to the reaction mixture, and rotary evaporation was performed to remove the THF. The resulting mixture was extracted with CHCl_3_ three times. The combined organic layers were dried over Na_2_SO_4_, filtered and concentrated under reduced pressure. The resulting product was purified by column chromatography on silica gel.

*N1-(3-(4-methyl-1H-imidazol-1-yl)-5-(trifluoromethyl)phenyl)-N3-(3-(trifluoromethyl)phenyl)isophthalamide* (**5**): yield 15%, white solid, m.p. 250–252 °C. ^1^H NMR (DMSO-d_6_): *δ* 10.88 (s, 1H, N*H*CO), 10.76 (s, 1H, N*H*CO), 8.61 (s, 1H), 8.31 (s, 1H), 8.27 (s, 1H), 8.21–8.23 (m, 3H), 8.18 (s, 1H), 8.09 (d, *J* = 8.3, 1H), 7.75–7.78 (m, 2H), 7.63 (t, *J* = 8.0, 1H), 7.48–7.50 (m, 2H), 2.18 (s, 3H, C*H*_3_). ^13^C NMR (DMSO-d_6_): *δ* 165.4, 165.3, 141.1, 139.7, 138.8, 137.9, 134.9, 134.8, 134.3, 131.1, 131.0, 130.7 (d, *J_F,C_* = 32.7, *C*-CF_3_), 130.0, 129.3 (d, *J_F,C_* = 31.9, *C*-CF_3_), 128.9, 127.1, 124.1 (d, *J_C,F_* = 272.0, *C*F_3_), 122.5 (d, *J_C,F_* = 272.6, *C*F_3_), 123.8, 120.1 (d, 2C), 116.3 (d, 2C), 114.9, 114.3 (d, 2C), 114.1, 111.8, 13.5. ^19^F NMR (DMSO-d_6_): *δ* -61.26, -61.33. HRMS (ESI): calcd. C_26_H_18_F_6_N_4_O_2_, [M+H]^+^ *m*/*z*: 533,1407, found: 533.1401. HPLC purity: 99.9%.

*N1-(4-((2-(methylcarbamoyl)pyridin-4-yl)oxy)phenyl)-N3-(3-(trifluoromethyl)phenyl)isophthalamide* (**6**): yield 23%, white solid, m.p. 258–260 °C. ^1^H NMR (DMSO-d_6_): *δ* 10.74 (s, 1H, N*H*CO), 10.60 (s, 1H, N*H*CO), 8.78 (q, 1H, *J* = 4.5, 9.4), 8.58 (s, 1H), 8.52 (d, 1H, *J* = 5.6), 8.28 (s, 1H), 8.18–8.20 (m, 2H), 8.10 (d, 1H, *J* = 8.3), 7.94–7.96 (m, 2H), 7.74 (t, 1H, *J* = 7.8), 7.63 (t, 1H, *J* = 7.9), 7.49 (d, 1H, *J* = 7.7), 7.41 (d, 1H, *J* = 2.6), 7.25–7.28 (m, 2H), 7.18 (dd, 1H, *J* = 5.5, 2.6), 2.8 (d, 3H, *J* = 4.8, C*H*_3_). ^13^C NMR (DMSO-d_6_): *δ* 165.8, 165.3, 165.0, 163.7, 152.4, 150.4, 148.9, 139.8, 136.8, 135.1, 134.6, 130.9, 130.8, 129.9, 129.3 (d, *J_C,F_* = 31.7, *C*-CF_3_), 128.8, 127.1, 124.1 (d, *J_C,F_* = 272.1, *C*F_3_), 123.0, 122.1, 121.3, 120.1 (d, 2C), 116.3 (d, 2C), 114.1, 108.7, 26.0. ^19^F NMR (DMSO-d_6_): *δ* -61.25. HRMS (ESI): calcd. C_28_H_21_F_3_N_4_O_4_, [M+H]^+^ *m*/*z*: 535,1588, found: 535.1588. HPLC purity: 99.9%.

*3-(4-(4-Methoxybenzoyl)piperazine-1-carbonyl)-N-(3-(trifluoromethyl)phenyl)benzamide* (**7**): yield 15%, white solid, m.p. 72–74 °C. ^1^H NMR (DMSO-d_6_): *δ* 10.64 (s, 1H, N*H*CO), 8.25 (s, 1H), 8.08–8.04 (m, 3H), 7.68–7.60 (m, 3H), 7.47 (d, 1H, *J* = 7.7), 7.41–7.40 (d, 2H), 7.00–6.96 (d, 2H), 3.79 (s, 3H, OC*H*_3_), 3.71–3.43 (m, 8H, *piperazine*). ^13^C NMR (DMSO-d_6_): *δ* 169.1, 168.4, 165.1, 160.2, 139.7, 135.7, 134.5, 130.2, 129.8, 129.3 (d, *J_C,F_* = 31.9, *C*-CF_3_), 129.1, 128.9, 128.7, 127.4, 126.2, 124.1 (d, *J_C,F_* = 272.2, *C*F_3_), 123.7, 120.0 (d, 2C), 116.3 (d, 2C), 113.6, 55.2, 47.6, 41.6. ^19^F NMR (DMSO-d_6_): *δ* -61.26. HRMS (ESI): calcd. C_27_H_24_F_3_N_3_O_4_, [M+H]^+^ *m*/*z*: 512,1792, found: 512.1791. HPLC purity: 99.4%.

*3-(4-(2-Fluorobenzoyl)piperazine-1-carbonyl)-N-(3-(trifluoromethyl)phenyl)benzamide* (**8**): yield 57%, white solid, m.p. 145–147 °C. ^1^H NMR (DMSO-d_6_): *δ* 10.62 (s, 1H, N*H*CO), 8.24 (s, 1H), 8.03–8.07 (m, 3H), 7.59–7.67 (m, 3H), 7.44–7.51 (m, 3H), 7.30 (bs, 2H), 3.65–3.76 (m, 4H, *piperazine*), 3.29–3.46 (m, 4H, *piperazine*). ^13^C NMR (DMSO-d_6_): *δ* 168.5, 165.1, 164.1, 157.5 (d, *J_C,F_* = 245.5, *C*F), 139.7, 135.7, 134.5, 131.6, 130.5, 130.2, 129.8, 129.3 (d, *J_F,C_* = 31.7, *C*-CF_3_), 128.9, 128.8, 128.7, 126.2, 124.9, 124.1 (d, *J_C,F_* = 272.3, *C*F_3_), 123.8, 123.5, 120.0, 116.4 (d, 2C), 115.8, 115.6. 46.6, 41.4. ^19^F NMR (DMSO-d_6_): *δ* -116.18, -61.25. HRMS (ESI): calcd. C_26_H_21_F_4_N_3_O_3_, [M+H]^+^ *m*/*z*: 500,1592, found: 500.1591. HPLC purity: 99.8%.

*3-(4-(3-Fluorobenzoyl)piperazine-1-carbonyl)-N-(3-(trifluoromethyl)phenyl)benzamide* (**9**): yield 44%, white solid, m.p. 97–101 °C. ^1^H NMR (DMSO-d_6_): *δ* 10.63 (s, 1H, N*H*CO), 8.24 (s, 1H), 8.03–8.07 (m, 3H), 7.60–7.66 (m, 3H), 7.46–7.51 (m, 2H), 7.26–7.30 (m, 3H), 3.70 (br.s, 4H, *piperazine*), 3.43 (br.s, 4H, *piperazine*). ^13^C NMR (DMSO-d_6_): *δ* 168.4, 167.7, 165.1, 161.8 (d, *J_C,F_* = 245.6, *C*F), 139.7, 137.8 (d, *J* = 7.0), 135.7, 134.5, 130.7 (d, *J* = 8.0), 130.2, 129.8, 129.3 (d, *J_C,F_* = 31.6, *C*-CF_3_), 128.9, 128.7, 126.2, 124.1 (d, *J_C,F_* = 272.4, *C*F_3_), 123.7, 122.9, 120.0 (d, 2C), 116.5, 116.4 (d, 2C), 114.0, 113.8, 46.8, 41.6. ^19^F NMR (DMSO-d_6_): *δ* -61.25, -116.18. HRMS (ESI): calcd. C_26_H_21_F_4_N_3_O_3_, [M+H]^+^ *m*/*z*: 500,1592, found: 500.1587. HPLC purity: 96.5%.

*N1,N3-bis(3-(4-methyl-1H-imidazol-1-yl)-5-(trifluoromethyl)phenyl)isophthalamide* (**10**)**:** yield 14%, white solid, m.p. 304–306 °C. ^1^H NMR (DMSO-d_6_): *δ* 11.58 (s, 2H, 2×N*H*CO), 9.66 (d, 2H, *J* = 1.2), 9.18 (s, 1H), 8.77 (s, 2H), 8.58 (s, 2H), 8.27 (dd, 2H, *J* = 7.7, 1.0), 8.02 (s, 2H), 7.95 (s, 2H), 7.76 (t, 1H, *J* = 7.8), 2.37 (s, 6H, 2×C*H*_3_). ^13^C NMR (DMSO-d_6_): *δ* 165.3, 141.5, 136.0, 134.3, 133.5, 132.0, 131.1, 130.9 (d, *J_F,C_* = 32.7, *C*-CF_3_), 129.3, 126.9, 123.3 (d, *J_F,C_* = 273.2, *C*F_3_), 117.4, 116.9, 114.1, 9.8. ^19^F NMR (DMSO-d_6_): *δ* -61.30. HRMS (ESI): calcd. C_30_H_22_F_6_N_6_O_2_, [M+H]^+^ *m*/*z*: 613,1781, found: 613.1776. HPLC purity: 96.3%. 

*N1-(3-(4-methyl-1H-imidazol-1-yl)-5-(trifluoromethyl)phenyl)-N3-(4-((2-(methylcarbamoyl)pyridin-4-yl)oxy)phenyl)isophthalamide* (**11**): yield 11%, white solid, m.p. 313–315 °C. ^1^H NMR (DMSO-d_6_): *δ* 10.90 (s, 1H, N*H*CO), 10.63 (s, 1H, N*H*CO), 8.75 (q, 1H, *J* = 4.4, 9.3), 8.62 (s, 1H), 8.53 (d, 1H, *J* = 5.8), 8.33 (s, 1H), 8.19–8.23 (m, 4H), 7.95 (d, 2H, *J* = 8.9), 7.76 (t, 2H, *J* = 7.8), 7.50 (s, 1H), 7.40 (d, 1H, *J* = 2.3), 7.27 (d, 2H), 7.18 (dd, 1H, *J* = 5.5, 2.6), 2.79 (d, 3H, C*H*_3_), 2.19 (s, 3H, C*H*_3_). ^13^C NMR (DMSO-d_6_): *δ* 165.8, 165.5, 165.0, 163.7, 152.4, 150.4, 149.0, 141.2, 138.9, 137.9, 136.8, 135.2, 134.9, 134.3, 131.1, 130.8 (d, *J_C,F_* = 32.2, *C*-CF_3_), 130.9, 128.8, 127.1, 123.7 (d, *J_F,C_* = 272.6, *C*F_3_) 122.1, 121.3, 114.9, 114.1–114.2 (m, 4C), 111.8, 108.8, 26.0, 13.5. ^19^F NMR (DMSO-d_6_): *δ* -61.32. HRMS (ESI): calcd. C_32_H_25_F_3_N_6_O_4_, [M+H]^+^ *m*/*z*: 615,1962, found: 615.1946. HPLC purity: 95.4%.

*N1-(3-(4-methyl-1H-imidazol-1-yl)-5-(trifluoromethyl)phenyl)-N3-(2-methyl-5-nitrophenyl)isophthalamide* (**12**): yield 13%, pale-yellow solid, m.p. 290–292 °C. ^1^H NMR (DMSO-d_6_): *δ* 10.90 (s, 1H, N*H*CO), 10.37 (s, 1H, N*H*CO), 8.62 (s, 1H), 8.38 (d, 1H, *J* = 2.5), 8.31 (s, 1H), 8.22–8.26 (m, 3H), 8.18 (s, 1H), 8.06 (dd, 1H, *J* = 8.4, 1.3), 7.76–7.79 (m, 2H), 7.61 (d, 1H, *J* = 8.6), 7.50 (s, 1H), 2.43 (s, 3H, C*H*_3_), 2.19 (s, 3H, C*H*_3_). ^13^C NMR (DMSO-d_6_): *δ* 165.4, 165.2, 145.7, 141.6, 141.1, 138.9, 137.9, 137.2, 134.9, 134.4, 131.5, 131.2, 131.0, 130.7 (d, *J_C,F_* = 32.5, *C*-CF_3_), 128.9, 127.3, 123.6 (d, *J_C,F_* = 272.9, *C*F_3_), 120.5, 114.9, 114.3, 114.2, 111.8, 18.2, 13.5. ^19^F NMR (DMSO-d_6_): *δ* -61.33. HRMS (ESI): calcd. C_26_H_20_F_3_N_5_O_4_, [M+H]^+^ *m*/*z*: 524,1540, found: 524.1527. HPLC purity: 98.0%.

*3-(4-(4-Methoxybenzoyl)piperazine-1-carbonyl)-N-(3-(4-methyl-1H-imidazol-1-yl)-5-(trifluoromethyl)-phenyl)benzamide* (**13**): yield 10%, white solid, m.p. 144–146 °C. ^1^H NMR (DMSO-d_6_): *δ* 10.78 (s, 1H, N*H*CO), 8.29 (s, 1H), 8.22 (s, 1H), 8.16 (s, 1H), 8.06–8.09 (m, 2H), 7.75 (s, 1H), 7.65–7.70 (m, 2H), 7.49 (s, 1H), 7.40–4.41 (d, 2H), 6.98–6.99 (d, 2H), 3.79 (s, 3H, OC*H*_3_), 3.71–3.42 (m, 8H, *piperazine*), 2.18 (s, 3H, C*H*_3_). ^13^C NMR (DMSO-d_6_): *δ* 169.3, 168.5, 165.3, 160.4, 141.2, 138.9, 138.0, 136.0, 135.0, 134.3, 130.9 (d, *J_C,F_* = 31.8, *C*-CF_3_), 130.5, 129.2, 129.1, 128.9, 127.5, 126.3, 123.6 (d, *J_C,F_* = 272.9, *C*F_3_), 115.0, 114.3 (d, 2C), 113.7, 111.9, 55.3, 47.0, 41.7, 13.5. ^19^F NMR (DMSO-d_6_): *δ* -61.33. HRMS (ESI): calcd. C_31_H_28_F_3_N_5_O_4_, [M+H]^+^ *m*/*z*: 592,2166, found: 592.2166. HPLC purity: 98.9%.

*3-(4-(2-Fluorobenzoyl)piperazine-1-carbonyl)-N-(3-(4-methyl-1H-imidazol-1-yl)-5-(trifluoromethyl)phenyl)benzamide* (**14**): yield 25%, white solid, m.p. 135–138 °C. ^1^H NMR (DMSO-d_6_): *δ* 10.77 (s, 1H, N*H*CO), 8.29 (s, 1H), 8.23 (s, 1H), 8.16 (s, 1H), 8.06 (bs, 2H), 7.75 (s, 1H), 7.64–7.70 (m, 2H), 7.44–7.50 (m, 2H), 7.30 (bs, 2H), 3.66–3.77 (m, 4H, *piperazine*), 3.35–3.45 (m, 4H, *piperazine*), 2.19 (s, 3H, C*H*_3_). ^13^C NMR (DMSO-d_6_): *δ* 168.4, 165.2, 164.2, 157.5 (d, *J_C,F_* = 245.7, *C*F), 141.1, 138.9, 137.9, 135.8, 134.9, 134.2, 131.6, 130.5 (d, *J_C,F_* = 32.9, *C*-CF_3_), 130.5, 128.9, 128.8, 128.7, 126.2, 124.9, 123.6 (d, *J_C,F_* = 272.7, *C*F_3_), 123.7, 123.6, 115.8, 115.7, 114.9, 114.2, 114.1 (d, 2C), 111.7, 46.5, 41.3, 13.7. ^19^F NMR (DMSO-d_6_): *δ* -56.58, -111,39. HRMS (ESI): calcd. C_30_H_25_F_4_N_5_O_3_, [M+H]^+^ *m*/*z*: 580,1966, found: 580.1963. HPLC purity: 96.9%.

*3-(4-(3-Fluorobenzoyl)piperazine-1-carbonyl)-N-(3-(4-methyl-1H-imidazol-1-yl)-5-(trifluoromethyl)phenyl)-benzamide* (**15**): yield 17%, white solid, m.p. 130–135 °C. ^1^H NMR (DMSO-d_6_): *δ* 10.78 (s, 1H, N*H*CO), 8.30 (s, 1H), 8.24 (s, 1H), 8.16 (s, 1H), 8.09–8.06 (m, 2H), 7.76 (s, 1H), 7.68–7.66 (d, 2H), 7.50 (s, 2H), 7.30–7.27 (m, 3H), 3.43–3.69 (m, 8H, *piperazine*), 2.19 (s, 3H, C*H*_3_). ^13^C NMR (DMSO-d_6_): *δ* 168.4, 167.9, 165.2, 161.7 (d, *J_C,F_* = 245.3, *C*F), 141.1, 138.7, 137.8, 137.7, 135.8, 134.9, 134.2, 130.8 (d, *J_C,F_* = 32.7, *C*-CF_3_), 130.7, 130.4, 130.3, 128.9, 128.8, 127.8, 123.5 (d, *J_C,F_* = 272.5, *C*F_3_), 123.0, 116.6, 116.4, 114.9, 114.3 (d, 2C), 114.2, 114.0, 113.9, 111.7, 46.8, 41.4, 13.4. ^19^F NMR (DMSO-d_6_): *δ* -112.20, -61.33. HRMS (ESI): calcd. C_30_H_25_F_4_N_5_O_3_, [M+H]^+^ *m*/*z*: 580,1966, found: 580.1965. HPLC purity: 95.4%.

*N1-(2-methyl-5-nitrophenyl)-N3-(4-((2-(methylcarbamoyl)pyridin-4-yl)oxy)phenyl)isophthalamide* (**16**): yield 9%, white solid, m.p. 138–141 °C. ^1^H NMR (DMSO-d_6_): *δ* 10.61 (s, 1H, N*H*CO), 10.35 (s, 1H, N*H*CO), 8.78 (q, 1H, *J* = 4.2, 9.1), 8.59 (s, 1H), 8.52 (d, 1H, *J* = 5.7), 8.38 (d, 1H, *J* = 2.4), 8.20–8.22 (m, 2H), 8.06 (dd, 1H, *J* = 8.6, 2.5), 7.93–7.96 (m, 2H), 7.75 (t, 1H, *J* = 7.7), 7.60 (d, 1H, *J* = 8.5), 7.41 (d, 1H, *J* = 2.6), 7.25–7.27 (m, 2H), 7.18 (dd, 1H, *J* = 5.6, 2.6), 2.79 (d, 3H, *J* = 4.8, C*H*_3_), 2.43 (s, 3H, C*H*_3_). ^13^C NMR (DMSO-d_6_): *δ* 165.8, 165.2, 165.0, 163.7, 152.4, 150.4, 148.9, 145.7, 141.6, 137.2, 136.8, 135.1, 134.2, 131.5, 130.9, 130.8, 128.7, 127.2, 122.1, 121.1, 120.5, 120.4, 114.0, 108.7, 25.9, 18.2. HRMS (ESI): calcd. C_28_H_23_N_5_O_6_, [M+H]^+^ *m*/*z*: 526,1721, found: 526.1721. HPLC purity: 97.6%.

*N1-(3-(4-methyl-1H-imidazol-1-yl)-5-(trifluoromethyl)phenyl)-N4-(3-(trifluoromethyl)phenyl)-terephthalamide* (**17**): yield 20%, white solid, m.p. 208–210 °C. ^1^H NMR (DMSO-d_6_): δ 10.85 (s, 1H, N*H*CO), 10.72 (s, 1H, N*H*CO), 8.31 (s, 1H), 8.27 (s, 1H), 8.22 (d, 1H, *J* = 1.3), 8.15–8.17 (m, 5H), 8.08 (d, 1H, *J* = 8.4), 7.76 (s, 1H), 7.63 (t, 1H, *J* = 8.0), 7.48–7.50 (m, 2H), 2.19 (s, 3H, C*H*_3_). ^13^C NMR (DMSO-d_6_): *δ* 165.4, 165.1, 141.0, 139.7, 138.9, 137.9, 137.4, 136.7, 134.9, 130.9 (d, *J_C,F_* = 31.9, *C*-CF_3_), 129.9, 129.3 (d, *J_C,F_* = 31.8, *C*-CF_3_), 127.9, 127.8, 124.1 (d, *J_C,F_* = 272.5, *C*F_3_), 123.9, 123.6 (d, *J_C,F_* = 272.5, *C*F_3_), 120.2 (d, 2C), 116.4 (d, 2C), 115.0, 114.3 (d, 2C), 114.1, 111.8, 13.5. ^19^F NMR (DMSO-d_6_): *δ* -61.27, -61.34. HRMS (ESI): calcd. C_26_H_18_F_6_N_4_O_2_, [M+H]^+^ *m*/*z*: 533,1407, found: 533.1401. HPLC purity: 98.9%.

*N1-(4-((2-(methylcarbamoyl)pyridin-4-yl)oxy)phenyl)-N4-(3-(trifluoromethyl)phenyl)terephthalamide* (**18**): yield 10%, white solid, m.p. 248–250 °C. ^1^H NMR (DMSO-d_6_): *δ* 10.71 (s, 1H, N*H*CO), 10.57 (s, 1H, N*H*CO), 8.77 (q, 1H, *J* = 4.4, 9.2), 8.52 (d, 1H, *J* = 5.6), 8.28 (s, 1H), 8.12–8.15 (m, 4H), 8.09 (d, 1H, *J* = 8.4), 7.95 (d, 2H), 7.63 (t, 1H, *J* = 8.0), 7.49 (d, 1H, *J* = 7.8), 7.41 (d, 1H, *J* = 2.5), 7.26 (d, 2H, *J* = 8.9), 7.18 (dd, 1H, *J* = 2.9, 6.0), 2.80 (d, 3H, *J* = 4.9, C*H*_3_). ^13^C NMR (DMSO-d_6_): *δ* 165.7, 165.1, 164.8, 163.7, 152.4, 150.3, 148.9, 139.7, 137.6, 138.9, 136.7, 129.9, 129.3 (d, *J_C,F_* = 31.4, *C*-CF_3_), 127.7, 124.0 (d, *J_C,F_* = 272.3, *C*F_3_), 123.8, 122.2, 121.2, 120.1 (d, 2C), 116.4 (d, 2C), 114.0, 108.7, 25.9. ^19^F NMR (DMSO-d_6_): *δ* -61.25. HRMS (ESI): calcd. C_28_H_21_F_3_N_4_O_4_, [M+H]^+^ *m*/*z*: 535,1588, found: 535.1578. HPLC purity: 99.1%.

*4-(4-(4-Methoxybenzoyl)piperazine-1-carbonyl)-N-(3-(trifluoromethyl)phenyl)benzamide* (**19**): yield 16%, white solid, m.p. 202–205 °C. ^1^H NMR (DMSO-d_6_): *δ* 10.64 (s, 1H, N*H*CO), 8.26 (s, 1H), 8.05 (d, 3H, *J* = 8.0), 7.59–7.63 (m, 3H), 7.47 (d, 1H, *J* = 7.7), 7.40–7.42 (m, 2H), 6.99 (d, 2H), 3.79 (s, 3H, OC*H*_3_), 3.69–3.39 (m, 8H, *piperazine*). ^13^C NMR (DMSO-d_6_): δ 169.1, 168.4, 165.2, 160.3, 139.7, 138.8, 135.2, 129.8, 129.3 (d, *J_C,F_* = 32.1, *C*-CF_3_), 129.1, 127.9, 127.3, 127.0, 124.1 (d, *J_C,F_* = 272.3, *C*F_3_), 123.7, 120.0 (d, 2C), 116.3 (d, 2C), 113.6, 55.2, 46.9, 41.5. ^19^F NMR (DMSO-d_6_): *δ* -61.26. HRMS (ESI): calcd. C_27_H_24_F_3_N_3_O_4_, [M+H]^+^ *m*/*z*: 512,1792, found: 512.1790. HPLC purity: 98.4%.

*4-(4-(2-Fluorobenzoyl)piperazine-1-carbonyl)-N-(3-(trifluoromethyl)phenyl)benzamide* (**20**): yield 35%, white solid, m.p. 215–217 °C. ^1^H NMR (DMSO-d_6_): *δ* 10.64 (s, 1H, N*H*CO), 8.27 (s, 1H), 8.05–8.06 (m, 3H), 7.60–7.63 (m, 3H), 7.44–7.52 (m, 3H), 7.32 (bs, 2H), 3.60–3.76 (m, 4H, *piperazine*), 3.35–3.42 (m, 4H, *piperazine*). ^13^C NMR (DMSO-d_6_): *δ* 168.6, 165.2, 164.1, 157.5 (d, *J_C,F_* = 245.4, *C*F), 139.7, 138.7, 135.2, 131.6, 130.5, 129.8, 129.3 (d, *J_C,F_* = 31.7, *C*-CF_3_), 128.8 (d, 2C), 128.7, 127.0, 124.9, 124.0 (d, *J_C,F_* = 272.3, *C*F_3_), 123.8, 123.6, 120.0 (d, 2C), 116.3 (d, 2C), 115.8, 115.6, 46.4, 41.2. ^19^F NMR (DMSO-d6): *δ* -61.26, -116.21. HRMS (ESI): calcd. C_26_H_21_F_4_N_3_O_3_, [M+H]^+^ *m*/*z*: 500,1592, found: 500.1586. HPLC purity: 99.5%.

*4-(4-(3-Fluorobenzoyl)piperazine-1-carbonyl)-N-(3-(trifluoromethyl)phenyl)benzamide* (**21**): yield 50%, white solid, m.p. 228–300 °C. ^1^H NMR (DMSO-d_6_): *δ* 10.64 (s, 1H, N*H*CO), 8.26 (s, 1H), 8.04–8.06 (m, 3H), 7.60–7.63 (m, 3H), 7.46–7.51 (m, 2H), 7.26–7.30 (m, 3H), 3.67 (br.s, 4H, *piperazine*), 3.43 (br.s, 4H, *piperazine*). ^13^C NMR (DMSO-d_6_): *δ* 168.4, 167.7, 165.2, 161.7 (d, *J_C,F_* = 245.4, *C*F), 139.7, 138.7, 137.8 (d, *J* = 6.8), 135.2, 130.7 (d, *J* = 7.7), 129.8, 129.3 (d, *J_C,F_* = 31.7, *C*-CF_3_), 127.9, 127.0, 124.1 (d, *J_C,F_* = 272.3, *C*F_3_), 123.7, 123.0, 120.0 (d, 2C), 116.6, 116.3 (d, 2C), 114.0, 113.8, 46.7, 41.6. ^19^F NMR (DMSO-d6): *δ* -61.26, -112.19. HRMS (ESI): calcd. C_26_H_21_F_4_N_3_O_3_, [M+H]^+^ *m*/*z*: 500,1592, found: 500.1591. HPLC purity: 97.1%.

*N1,N4-bis(3-(4-methyl-1H-imidazol-1-yl)-5-(trifluoromethyl)phenyl)terephthalamide* (**22**): yield 27%, white solid, m.p. 295–296 °C. ^1^H NMR (DMSO-d_6_): *δ* 11.47 (s, 2H, 2×N*H*CO), 9.68 (d, 2H), 8.73 (s, 2H), 8.47 (s, 2H), 8.30 (s, 4H), 8.02 (s, 2H), 7.96 (s, 2H), 2.37 (s, 6H, 2×C*H*_3_). ^13^C NMR (DMSO-d_6_): *δ* 165.2, 141.4, 136.6, 136.0, 134.2, 131.0, 130.7 (d, *J_C,F_* = 32.7, *C*-CF_3_), 128.1, 123.3 (d, *J_C,F_* = 272.8, *C*F_3_), 117.3, 117.1, 114.0, 9.8. ^19^F NMR (DMSO-d_6_): *δ* -61.30. HRMS (ESI): calcd. C_30_H_22_F_6_N_6_O_2_, [M+H]^+^ *m*/*z*: 613,1781, found: 613.1778. HPLC purity: 96.9%.

*N1-(3-(4-methyl-1H-imidazol-1-yl)-5-(trifluoromethyl)phenyl)-N4-(4-((2-(methylcarbamoyl)pyridin-4-yl)oxy)phenyl)terephthalamide* (**23**): yield 12%, white solid, m.p. 299–300 °C. ^1^H NMR (DMSO-d_6_): *δ* 10.85 (s, 1H, N*H*CO), 10.59 (s, 1H, N*H*CO), 8.78 (q, 1H, *J* = 4.6, 9.4), 8.52 (d, 1H, *J* = 5.6), 8.32 (s, 1H), 8.23 (d, 1H, *J* = 1.0), 8.13–8.17 (m, 5H), 7.93–7.95 (m, 2H), 7.77 (s, 1H), 7.50 (s, 1H), 7.40 (d, 1H, *J* = 2.5), 7.25–7.27 (m, 2H), 7.18 (dd, 1H, *J* = 2.6, 5.6), 2.79 (d, 3H, *J* = 4.9, C*H*_3_), 2.19 (s, 3H, C*H*_3_). ^13^C NMR (DMSO-d_6_): *δ* 165.8, 165.3, 164.8, 163.7, 152.4, 150.4, 148.9, 141.0, 138.9, 137.9 137.7, 136.7, 136.5, 134.9, 130.8 (d, *J_C,F_* = 31.9, *C*-*C*F_3_), 127.8 (d, 2C), 123.6 (d, *J_C,F_* = 273.3, *C*F_3_), 122.2, 121.2, 115.0, 114.3 (d, 2C), 114.1 (d, 2C), 111.8, 108.7, 25.9, 13.5. ^19^F NMR (DMSO-d_6_): *δ* -61.30. HRMS (ESI): calcd. C_32_H_25_F_3_N_6_O_4_, [M+H]^+^ *m*/*z*: 615,1962, found: 615.1955. HPLC purity: 97.0%.

*N1-(3-(4-methyl-1H-imidazol-1-yl)-5-(trifluoromethyl)phenyl)-N4-(2-methyl-5-nitrophenyl)terephthalamide* (**24**): yield 20%, pale-yellow solid, m.p. 269–270 °C. ^1^H NMR (DMSO-d_6_): *δ* 10.85 (s, 1H, N*H*CO), 10.33 (s, 1H, N*H*CO), 8.38 (d, 1H, *J* = 2.4), 8.32 (s, 1H), 8.23 (d, 1H, *J* = 1.2), 8.15–8.19 (m, 5H), 8.07 (dd, 1H, *J* = 2.5, 8.4), 7.77 (s, 1H), 7.60 (d, 1H, *J* = 8.6), 7.51 (s, 1H), 2.42 (s, 3H, C*H*_3_), 2.19 (s, 3H, C*H*_3_). ^13^C NMR (DMSO-d_6_): *δ* 165.2, 165.0, 145.7, 141.6, 141.0, 138.8, 137.9, 137.1, 137.0, 136.7, 134.9, 131.5, 130.8 (d, *J_C,F_* = 31.8, *C*-CF_3_), 127.9 (d, 2C), 123.5 (d, *J_C,F_* = 273.6, *C*F_3_), 120.5 (d, 2C), 115.0, 114.3 (d, 2C), 114.1, 111.8, 18.2, 13.4. ^19^F NMR (DMSO-d_6_): *δ* -61.33. HRMS (ESI): calcd. C_26_H_20_F_3_N_5_O_4_, [M+H]^+^ *m*/*z*: 524,1540, found: 524.1533. HPLC purity: 99.2%.

*4-(4-(4-methoxybenzoyl)piperazine-1-carbonyl)-N-(3-(4-methyl-1H-imidazol-1-yl)-5-(trifluoromethyl)-phenyl)benzamide* (**25**): yield 14%, white solid, m.p. 212–214 °C. ^1^H NMR (DMSO-d_6_): *δ* 10.78 (s, 1H, N*H*CO), 8.30 (s, 1H), 8.23 (s, 1H), 8.16 (s, 1H), 8.07 (d, 2H, *J* = 8.1), 7.75 (s, 1H), 7.63 (d, 2H, *J* = 8.1), 7.50 (s, 1H), 7.41 (d, 2H, *J* = 8.4), 6.99 (d, 2H, *J* = 8.4), 3.79 (s, 3H), 3.70–3.39 (m, 8H, *piperazine*), 2.19 (s, 3H, C*H*_3_). ^13^C NMR (DMSO-d_6_): *δ* 169.1, 168.3, 165.3, 160.3, 141.1, 139.0, 138.7, 137.8, 134.8 (d, 2C), 130.8 (d, *J_C,F_* = 32.6, *C*-CF_3_), 129.1, 127.9, 127.3, 127.1, 123.5 (d, *J_C,F_* = 273.2, *C*F_3_), 114.9, 114.2 (d, 2C), 113.6, 111.8, 55.2, 46.8, 41.5, 13.4. ^19^F NMR (DMSO-d_6_): *δ* -61.33. HRMS (ESI): calcd. C_31_H_28_F_3_N_5_O_4_, [M+H]^+^ *m*/*z*: 592,2166, found: 592.2158. HPLC purity: 98.3%.

*4-(4-(2-fluorobenzoyl)piperazine-1-carbonyl)-N-(3-(4-methyl-1H-imidazol-1-yl)-5-(trifluoromethyl)phenyl)-benzamide* (**26**): yield 18%, white solid, m.p. 142–145 °C. ^1^H NMR (DMSO-d_6_): *δ* 10.77 (s, 1H, N*H*CO), 8.29 (s, 1H), 8.21 (s, 1H), 8.15 (s, 1H), 8.06 (br.s, 2H), 7.75 (s, 1H), 7.63 (br.s, 2H), 7.44–7.51 (m, 2H), 7.31 (bs, 2H), 3.42–3.75 (m, 8H, *piperazine*), 2.19 (s, 3H, C*H*_3_). ^13^C NMR (DMSO-d_6_): *δ* 168.3, 165.3, 164.2, 157.5 (d, *J_C,F_* = 245.6, *C*F), 141.1, 138.9, 138.8, 137.9, 134.9, 131.6 (d, *J* = 7.6), 130.8 (d, *J_C,F_* = 32.3, *C*-CF_3_), 128.9, 128.8, 127.9, 124.9, 123.7, 123.6, 123.5 (d, *J_C,F_* = 272.7, *C*F_3_), 115.8, 115.7, 114.9, 114.2 (d, 2C), 114.1, 111.7, 46.6, 41.3, 13.5. ^19^F NMR (DMSO-d_6_): *δ* -116.19, -61.34. HRMS (ESI): calcd. C_30_H_25_F_4_N_5_O_3_, [M+H]^+^ *m*/*z*: 580,1966, found: 580.1965. HPLC purity: 98.0%.

*4-(4-(3-fluorobenzoyl)piperazine-1-carbonyl)-N-(3-(4-methyl-1H-imidazol-1-yl)-5-(trifluoromethyl)phenyl)-benzamide* (**27**): yield 12%, white solid, m.p. 110–113 °C. ^1^H NMR (DMSO-d_6_): *δ* 10.77 (s, 1H, N*H*CO), 8.29 (s, 1H), 8.21 (s, 1H), 8.15 (s, 1H), 8.07–8.06 (d, 2H), 7.75 (s, 1H), 7.63–7.61 (d, 2H), 7.51–7.49 (m, 2H), 7.30–7.26 (m, 3H), 3.42–3.72 (m, 8H, *piperazine*), 2.19 (s, 3H, C*H*_3_). ^13^C NMR (DMSO-d_6_): *δ* 168.3, 167,7, 165.3, 161.7 (d, *J_C,F_* = 245.1, *C*F), 141.1, 138.9, 138.8, 137.9, 137.8 (d, 2C), 134.9, 134.8, 130.8 (d, *J_C,F_* = 32.8, *C*-CF_3_), 130.7 (d, 2C), 127.9, 127.1, 123.5 (d, *J_C,F_* = 272.7, *C*F_3_), 123.0, 116.6, 116.5, 114.9, 114.2 (d, 2C), 114.1, 114.0, 113.9, 111.7, 46.6, 41.3, 13.4. ^19^F NMR (DMSO-d_6_): *δ* -112.14, -61.34. HRMS (ESI): calcd. C_30_H_25_F_4_N_5_O_3_, [M+H]^+^ *m*/*z*: 580,1966, found: 580.1960. HPLC purity: 98.8%.

*N1-(2-methyl-5-nitrophenyl)-N4-(4-((2-(methylcarbamoyl)pyridin-4-yl)oxy)phenyl)terephthalamide* (**28**): yield 10%, pale-yellow solid, m.p. 285–287 °C. ^1^H NMR (DMSO-d_6_): *δ* 10.58 (s, 1H, N*H*CO), 10.32 (s, 1H), 8.79 (d, 1H, *J* = 4.7), 8.53 (d, 1H, *J* = 5.5), 8.38 (d, 1H, *J* = 2.1), 8.13–8.16 (m, 4H), 8.05–8.07 (dd, 1H, *J* = 2.2, 8.4), 7.95–7.96 (m, 2H), 7.60 (d, 1H, *J* = 8.4), 7.41 (d, 1H, *J* = 2.3), 7.26–7.27 (m, 2H), 7.18 (dd, 1H, *J* = 4.5, 5.5), 2.79 (d, 3H, *J* = 4.8, C*H*_3_), 2.42 (s, 3H, C*H*_3_). ^13^C NMR (DMSO-d_6_): *δ* 165.7, 165.0, 164.7, 163.7, 152.4, 150.3, 148.9, 145.7, 141.6, 137.6, 137.1, 136.7, 136.5, 131.5, 127.8, 127.7, 122.2, 121.2, 120.5, 120.4, 114.0, 108.7, 25.9, 18.2. HRMS (ESI): calcd. C_28_H_23_N_5_O_6_, [M+H]^+^ *m*/*z*: 526,1721, found: 526.1718. HPLC purity: 97.9%.

### 2.2. Cell Proliferation Assay

Tests to determine the effects of the synthesized compounds on cell viability and the half-maximal inhibitory concentration (IC_50_) were performed using the MTT assay, as described in our prior work [32].

### 2.3. Kinase Inhibitory Assay

The ADP-Glo kinase assay (Promega, Madison, WI, USA) was used to screen compounds **5**, **8**–**10**, **14**, **17**, **19** and **24** for the tyrosine kinase (Kinase Selectivity Profiling System TK-1, Promega, Madison, WI, USA) and serine/threonine kinase (Kinase Selectivity Profiling System Other/CK-1, Promega, Madison, WI, USA) inhibition effects according to the manufacturer’s technical manual. The luminescence data were analyzed as described in our prior work [32].

### 2.4. Cell Apoptosis Assay

K562 tumor cells were plated on a 6-well plate at a density of 400,000 per well. Test compounds **5, 14** and **15** were added at concentrations of 1, 5 and 10 μM in duplicate for 48 h. The methodology and analysis of the obtained data were performed as described in our prior work [32].

### 2.5. In Vitro Cell Cycle Effects

Tumor cells were seeded on a 6-well plate at a density of 400,000 per well for K562. Test compounds **5, 14** and **15** were added at concentrations of 1, 5 and 10 μM in duplicate for 48 h. The methodology and analysis of the obtained data were performed as described in our prior work [32].

### 2.6. ROS Determination

The redox status was assessed using the K562 cell line. A total of 400,000 of cells per well were seeded on a 6-well plate and treated with compounds **5**, **14** and **15**, as well as lapatinib and sorafenib, at concentrations of 5, 10, and 20 μM in duplicate for 48 h. After treatment, staining was performed with a 10 µM concentration of 2′,7′-dichlorodihydrofluorescein diacetate (Acros Organics, Somerville, NJ, USA) for 30 min at 37 °C in the dark. Then, the cells were washed to remove the 2′,7′-dichlorodihydrofluorescein diacetate by centrifugation at 1000 rpm for 5 min. The measurements were conducted using a Cytomics FC500 Beckman Coulter flow cytometer (FL1 channel for DCF).

### 2.7. Molecular Docking and Molecular Dynamics

The docking and MD protocols were the same as those described in our previous publication [32], except for the fact that QVina 2 (Nanyang Technological University, Singapore, 2015) [33] was used as docking software and the Cactus web server was used [34] to obtain 3D structures of the ligands.

## 3. Results and Discussion

### 3.1. Molecular Design 

**Figure 1:**
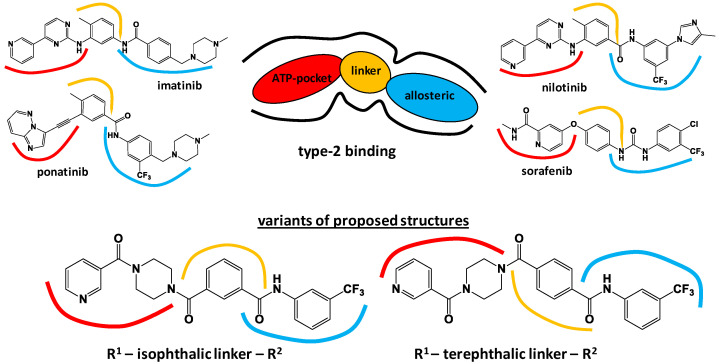
Pharmacophore-based approach to the design of target compounds. Structural fragments that bind to the ATP pocket are highlighted in red, and those binding to the allosteric pocket are in blue. Linkers are labeled in yellow.

As shown in our previous work [31,32], the structure of a typical type-2 protein kinase inhibitor can be decomposed into three main fragments (Figure 1). Since the reengineering of imatinib into nilotinib, 3-(trifluoromethyl)aniline has emerged as the best choice for handling a kinase’s allosteric site. While type-2 inhibitors use various heterocyclic systems to mimic ATP interactions in the ATP-binding site, the linker is responsible for the spatial orientation of the inhibitor’s ATP and allosteric fragments. Taking these considerations into account, we proposed examining various linkers attached to the 3-(trifluoromethyl)aniline moiety as a rational strategy towards the development of novel chemical scaffolds that are able to target protein kinases. As part of this approach, we used 4-methylbenzamide linker to develop promising anticancer agents [31,32]. In the present study, we propose using isophthalic and terephthalic linkers as a framework for constructing novel type-2 inhibitors. Compared to 4-methylbenzamide, these phthalic linkers have some advantages. Firstly, they are more rigid, which can render inhibitor positioning more restrained and stable. Secondly, a non-allosteric amide bond may be involved in hydrogen bonding in the ATP pocket, resulting in additional interactions.

It should be noted that phthalic linkers with adjacent 1,2-carbonyl groups were not considered as a good choice, since they would not provide the optimal angle between the ATP and allosteric pharmacophoric groups of the potential inhibitors. In this context, both isophthalic and terephthalic linkers seemed to be rational choices, as they have more structural similarity with the known inhibitors.

In general, we suggest that, at least theoretically, the binding effects of the designed isophthalic derivatives and their terephthalic counterparts to the protein kinase receptors are quite similar. Considering our primary structures of focus (Figure 1), which are closely related to previously discovered anticancer 4-methylbenzamide analogues [31,32], the 3-trifluoromethylaniline should rest in the allosteric pocket, as it does in the case of imatinib/nilotinib. Phthalic acid fragments, whether isophthalic or terephthalic, is intended to play the role of a linker, ensuring the ability of the substituted piperazine to reach the ATP pocket. We supposed that the piperazine ring is flexible enough to fit into the ATP pocket, no matter which type of linker it is attached to. However, clearly, one of those linker types must provide a more favorable placement of piperazine in the ATP pocket than the other, with more electrostatic and van der Waals contacts. In the design stage, it was not particularly clear which phthalic linker was more promising; thus, two series were synthesized.

In fact, we used a combinational strategy with various amines to obtain target phthalimides so as to gain more knowledge about promising linker types and structural fragments, as shown in the Chemistry section. For example, to target the allosteric pocket of a kinase, we might consider the nitro-group as an alternative pharmacophore to the trifluoromethyl group. The nitro-group is polar and can engage in specific interactions with a protein. In the case of strong observed in vitro activity, the bioisosteric replacement of the nitro-group (with oxazole, for example) could be a valuable step towards the identification of a promising drug candidate.

### 3.2. Chemistry

**Scheme 1:**
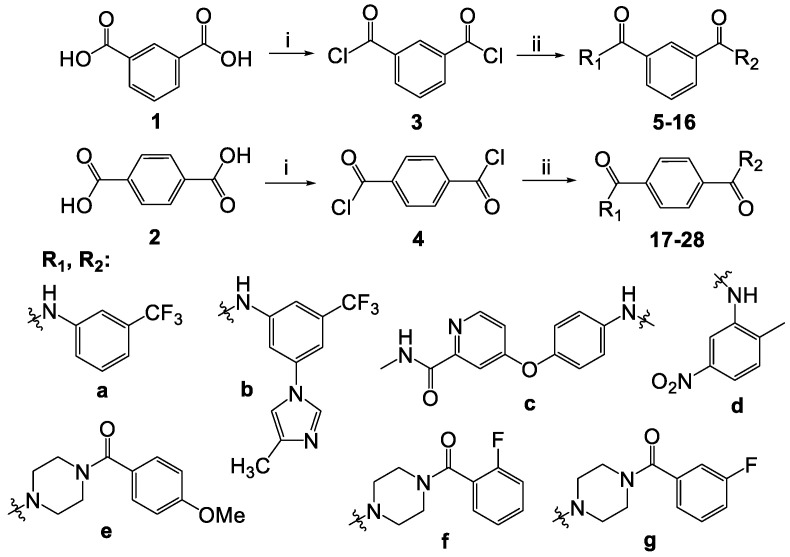
Synthetic route of derivatives **5**–**28**. Reagents and conditions: (**i**) (COCl)_2_, DMF, THF, rt, 3 h; (**ii**) R_1_H, R_2_H, THF, Et_3_N, rt.

Twenty-four target compounds were obtained by a two-step synthesis, starting with isophthalic acid (**1**) and terephthalic acid (**2**). In the first stage, corresponding phthalic acids were treated with a 4-fold excess of oxalyl chloride in tetrahydrofuran and DMF (Scheme 1). In the second stage, the resulting acid dichlorides **3** and **4** (1.1 equivalent) were subsequently condensed with the amines **a**–**g**. The last reaction was carried out in tetrahydrofuran in the presence of an equimolar amount of Et_3_N at room temperature for 3 h. The yields of the target compounds **5**–**28** after isolation on silica gel varied within the range of 9–57%. The structures and yields of the synthesized compounds are presented in Table 1.

The structures of the synthetic products were established by ^1^H, ^13^C and ^19^F NMR spectroscopy and high-resolution mass spectrometry (HRMS).

The ^1^H and ^13^C NMR spectra of isophthalic and terephthalic derivatives **5**–**28** are complicated by crowded aromatic regions (see Supplementary Materials). However, some characteristic features can be distinguished. In the ^1^H NMR spectra, the weakest signals correspond to protons of secondary amide bonds and are defined by a chemical shift from 11.58 to 10.32 ppm. For compounds ***7***–**9**, **13**–**15**, **19**–**21** and **25**–**27**, containing a piperazine moiety, CH_2_ resonance signals can be observed in the upfield at 3.29–3.76 ppm.

The ^19^F NMR spectra of the target compounds, except for **16** and **28**, exhibit the characteristic peaks near -61.25 ppm assigned to the F-atoms of the 5-(trifluoromethyl)phenyl fragment [35]. In the similar spectra of compounds **8**, **9**, **14** and **15** as well as **20**, **21**, **26** and **27**, additional signals appear at -116.3 and -112.2 ppm, respectively, which are related to the fluorine atom of the 2(3)-fluorobenzoic acid moiety, in addition to the CF_3_ signal. The C-F coupling constant (*J*_C,F_) for the CF_3_ and 2(3)-F-benzoic acid moiety was observed at 275 Hz and 245 Hz, respectively.

### 3.3. Biological Studies

#### 3.3.1. Antiproliferative Activity

The target compounds **5**, **6**, **8**–**12**, **14**, **15**, **17**–**22** and **24**–**26** were tested for antiproliferative activity against six cancer cell lines: K562 (chronic myelogenous leukemia), HL-60 (promyelocytic leukemia), MCF-7 (breast adenocarcinoma), HepG2 (human liver cancer), A549 (lung carcinoma) and OKP-GS (renal cell carcinoma). The RPMI 1788 cells (B lymphocyte, human) were used as a control to evaluate the system toxicity of the studied compounds to normal cells. Sorafenib, nilotinib and lapatinib were utilized as reference standards for comparison.

The IC_50_ values are presented in Table 2. In the case of nilotinib, the IC_50_ value for the chronic myeloid leukemia cell line (K562) was determined to be less than 0.1 μmol, which is a marker of Bcr-Abl protein expression and is consistent with data obtained previously [36]. The antiproliferative activity of nilotinib and lapatinib for the studied cell cultures was characterized by IC_50_ values ranging from 2 to 27 µM.

It was found that, in general, the tumor cell cultures were more sensitive to isophthalic acid derivatives than to terephthalic acid ones. Thus, compound **5**, containing 3-(4-methyl-1H-imidazol-1-yl)-5-(trifluoromethyl)aniline and 5-(trifluoromethyl)aniline fragments, showed the highest inhibitory activity against four cancer cell lines, K562, HL-60, MCF-7 and HepG2 (IC_50_ = 3.42, 7.04, 4.91 and 8.84 µM, respectively), compared to those of the corresponding derivatives of terephthalic acid **17**, which had almost no effect on the cell lines’ viability. Compounds **14** and **15**, containing a 2(3)-fluorobenzoyl piperazine moiety **f**, **g**, showed moderate inhibitory activity (IC_50_ = 7.71 and 10.51 μM, respectively) comparable to that of lapatinib against the K562 cell line. The replacement of the isophthalic linker with a terephthalic linker in these compounds led to a 2-fold decrease in their activity.

The other synthesized compounds showed moderate to weak antiproliferative activity against most of the cancer cell lines (IC_50_ ranging from 15 to 49 μM), with most of the active compounds being related to derivatives of isophthalic acid. The introduction of amines **c** and **d** into the molecule with an isophthalic linker resulted in a lack of sensitivity of the cancer cells in the studied concentration range (compounds **6**, **11** and **12**). The A549 cell line was insensitive to all the synthesized compounds.

According to Table 2, most of the synthesized compounds were toxic to normal RPMI 1788 cells. However, this finding is consistent with the results for reference compounds inhibiting the growth of normal human dermal fibroblasts (NHDF) based on in vitro experiments [37,38].

#### 3.3.2. Kinase Inhibitory Assays

The selectivity of the potential kinase inhibitor against a specific protein kinase or a specific family of kinases was investigated using the Kinase Selectivity Profiling System TK-1, Other/CK-1 and ADP-Glo™ Kinase assay kit (Promega, Madison, WI, USA). The target compounds **5**, **8**–**10**, **14**, **17**, **19** and **24** were tested against eight receptor tyrosine kinases, including EGFR, HER2 and HER4 (epidermal growth factor receptors), IGF1R (insulin-like growth factor 1 receptor), InsR (insulin receptor), KDR (vascular endothelial growth factor receptor 2—VEGFR-2), PDGFRα and PDGFRβ (platelet-derived growth factor receptors), and two serine/threonine kinases, Aurora A and B. Lapatinib, sorafenib and sunitinib were taken as comparison compounds. The inhibition percentage results at a concentration of 10 µM are presented in Table 3.

Isophthalic derivative **9** revealed a high potency against EGFR and HER2, at the levels of 90% and 64%, respectively, being comparable to lapatinib at 10 µM (Table 3). Kinase insert domain receptor, KDR, is a tyrosine kinase receptor for VEGFs that plays a central role in tumor angiogenesis and is a major therapeutic target for the inhibition of angiogenesis and tumor growth. Compounds **5**, **8**–**10**, **14** and **19** showed inhibitory activity against KDR in the range of 16–34% at a concentration of 10 µM. 

Compound **5** exhibited a high inhibitory activity against PDGFRα, at the level of 73%, although this was slightly lower compared to sorafenib. The activity of the isomeric terephthalic analogue **17** did not exceed 20%. The influence of the linker type on the possible binding of isomers **5** and **17** in the ATP pocket of a kinase was clear. In this sense, the higher activity of the isophthalic derivatives, compared to their terephthalic analogues, can be explained by the peculiarities of the geometry of the molecules.

It is noteworthy that none of the studied phthalic acid analogues were shown to be active against Aurora A and B (serine/threonine kinases).

#### 3.3.3. In Vitro Cell Cycle Effects of Compounds **5**, **14** and **15**

**Figure 2:**
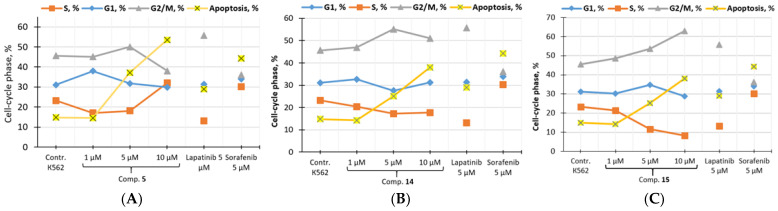
Cell cycle inhibition of K562 cells treated with different concentrations of compounds **5** (**A**), **14** (**B**) and **15** (**C**), with 5 µM of sorafenib and lapatinib as a reference compound.

As shown above, compounds **5**, **14** and **15** had significant antiproliferative activities, which is why their influences on the cell cycle and apoptosis level were determined, with the aim of understanding whether these effects are cytostatic or cytotoxic. In the cell cycle experiments, K562 cells were used. The cells were treated for 48 h with 1 µM, 5 µM and 10 µM concentrations of compounds **5**, **14** and **15**, as well as lapatinib and sorafenib, respectively. For the determination of changes in the distribution of cells in the phases of the cell cycle, the widely employed method of staining the cells with propidium iodide was used [39]. The results are shown in Table 4 and Figure 2.

The cell cycle analysis showed a decrease in the number of K562 cells in phase S to 17.0% and 18.14%, respectively, when treated with isophthalic analogue **5** at concentrations of 1 µM and 5 µM, down from 23.27% in the control (DMSO). While the number of cells in the G2/M phase decreased from 45.6% in the control (DMSO) to 38.0%, apoptosis increased to 53.50% (Table 4, Figure 2A).

In the case of the isomeric ortho- and meta-isophthalic fluorine derivatives **14** and **15**, for compound **15**, a pronounced dose-dependent effect was observed in reducing the number of K562 cells in the S phase from 23.3% in the control (DMSO) to 11.59% (5.0 μM) and 8.16% (10, 0 μM) after 48 h. With a practically unchanged number of cells in the G1 phase, the number of cells in the G2/M phase increased from 45.62% in the control (DMSO) to 53.65% (5.0 μM) and 62.99% (10 μM) for compound **15** and up to 55.16% (5.0 µM) and 51.08% (10 µM) for isomer **14**.

The arrest of division in the G2/M phase is one of the main mechanisms of response to cell DNA damage, and the molecular mechanisms of the initiation of this arrest are being actively studied. It is likely that the action of ortho-analogue **15** may induce double-stranded DNA breaks and increase the proportion of cells that are delayed in the G2/M phase in a dose-dependent manner. At the same time, the number of cells passing into apoptosis increases linearly from 17.90% (1.0 µM) to 32.72% (10.0 µM). Based on the obtained results, it can be assumed that some of the cells overcome the block in G2/M and enter the G1 phase, while the rest undergo apoptosis. In the case of isomeric compound **14**, this tendency is less pronounced. It should be noted that the phase distribution of the cell cycle of compound **15** is similar to the action of lapatinib [40]. The data obtained suggest that compounds **5**, **14** and **15** have cytostatic effects, as they reduce the number of cells in the S phase and cause an accumulation of cells in the G2/M phase. This cell cycle arrest then results in apoptosis.

#### 3.3.4. Apoptosis Assay

One of the major modes of action of chemotherapeutic drugs may be the activation of apoptosis. Sorafenib has previously been shown to not only exert anti-tumor effects by inhibiting kinases involved in cell proliferation and survival but also induce apoptosis and necrosis in various types of cancer [17].

**Figure 3:**
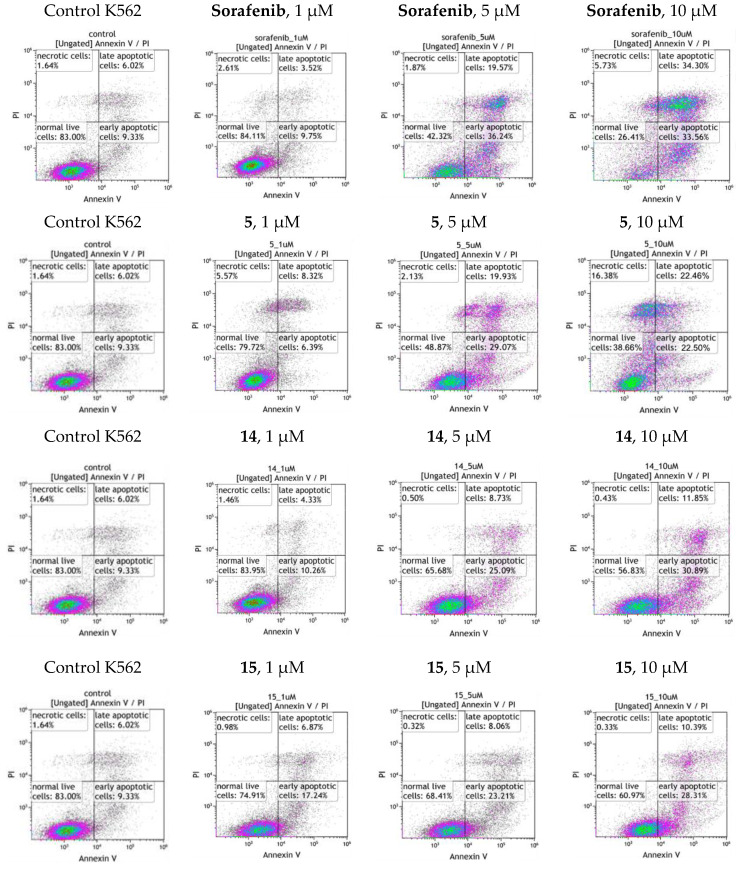
Effects of compounds **5**, **14** and **15** on apoptosis in K562 cells compared with the positive control, sorafenib.

The effects of compounds **5**, **14** and **15** on cell apoptosis were examined using the Annexin V-FITC Kit (Beckman Coulter, Brea, CA, USA) according to the protocols provided. The Annexin V-FITC Kit is an apoptosis detection kit based on the binding properties of annexin V to phosphatidylserine (PS) and on the DNA intercalating capability of propidium iodide (PI) [41]. K562 cells were treated with compound **5**, **14** and **15** at 1, 5 and 10 μM for 48 h, after which annexin V/PI binding was measured by flow cytometry. The dot plot flow cytometry data of the stained cells are displayed in Figure 3.

**Figure 4:**
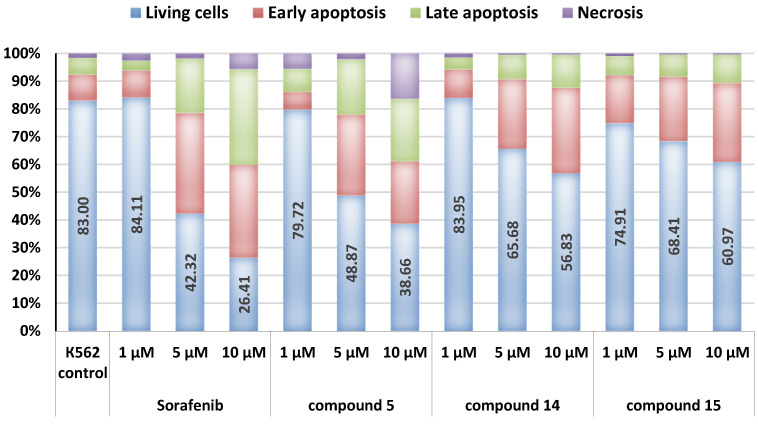
Percentage of apoptosis and necrosis induced by compounds **5**, **14**, **15** and sorafenib in the K562 cell line.

The results of this assay revealed that isophthalic derivative **5**, as well as the reference drug, mostly induced both early and late apoptosis in the K562 cell line (Figure 4). The treatment of K562 cells with compound **5** and sorafenib at 5 µM resulted in an increase in the apoptotic cell percentage for the early stage of apoptosis, rising from 9.33% for the untreated control cells to 29.07% and 36.2%, respectively. The percentage of apoptotic cells in the late stage for these two compounds at 5 µM was 19.93% and 19.57% (6.02% for the control).

In addition, compound **5** was found to promote dynamic cellular activities in a dose-dependent manner. When the concentration was increased from 5 to 10 µM, the percentage of normal K562 cells decreased to 38.66%, while necrosis reached 16.38%, which was three times more than that of sorafenib under similar conditions (5.73%). Thus, the overall cell population shift indirectly indicated that there are differences in the mechanism of cell death.

The isomeric derivatives of isophthalic acid **14** and **15** did not have necrotic effects on the K562 cells, while the percentage of living cells decreased slightly depending on the concentration and was 56.83% and 60.97%, respectively, versus 26.41% for sorafenib at 10 µM. 

#### 3.3.5. Reactive Oxygen Species (ROS)

The intracellular level of ROS was assessed using the K562 cell line with the help of a fluorescent probe, 2ʹ,7ʹ-dichlorodihydrofluorescein diacetate and flow cytometry [42].

**Figure 5:**
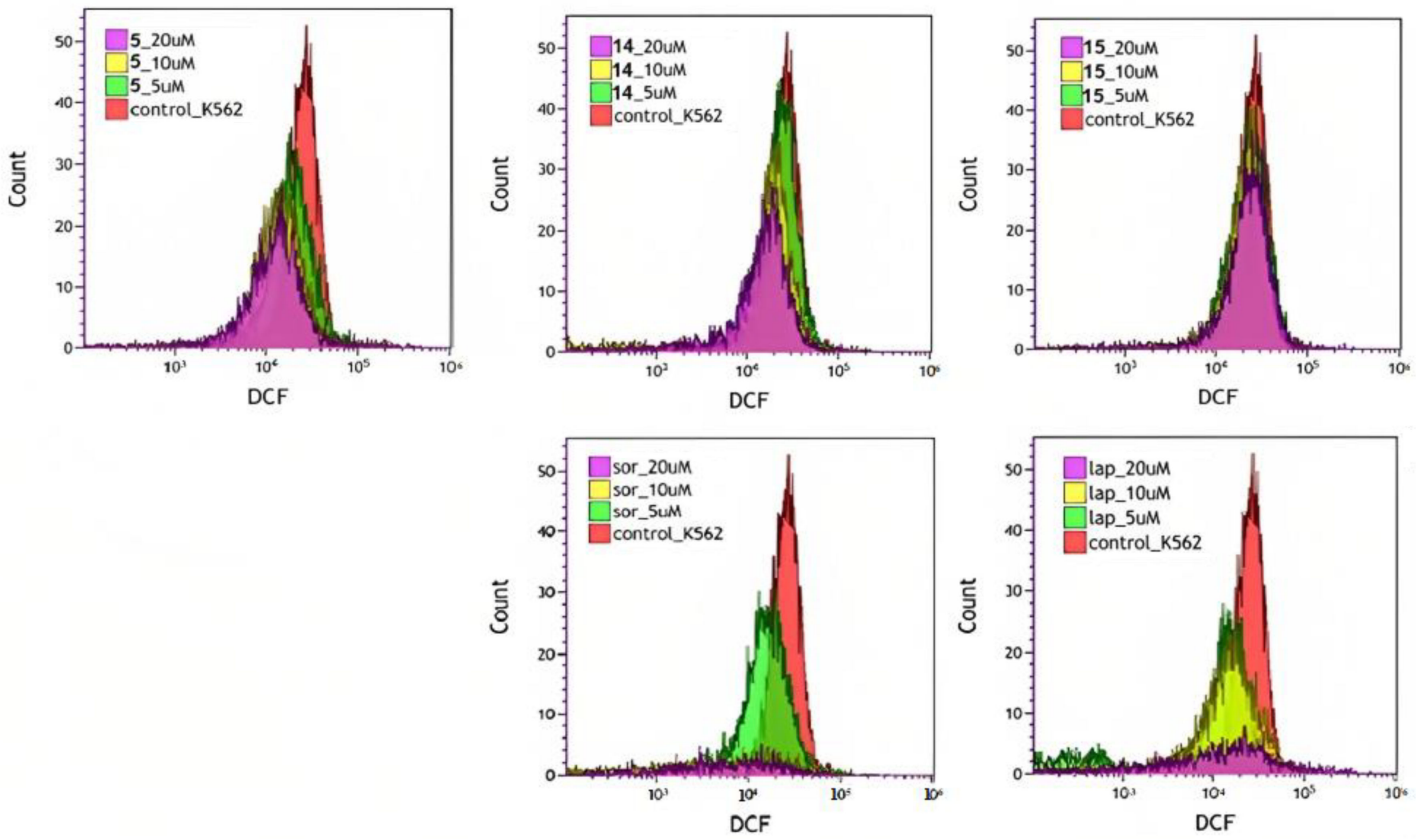
ROS level histogram under the influences of compounds **5**, **14** and **15**.

The data obtained for compound **5** showed a decrease in ROS of up to 62.9% at 20 μM (Table 5, Figure 5) and, at the same time, a decrease in the number of cells that is probably indirectly associated with a rather high level of necrosis caused by the action of this compound on the K562 cells. 

In the case of isomeric derivatives **14** and **15**, a dose-dependent decrease in ROS was observed for the *ortho-*analogue **14**, while the *meta-*analogue **15** showed only a basic level of ROS accumulation in the presence of similar concentrations in this cell line. To date, a number of studies have shown that ROS can play a decisive role in the death of tumor cells through various mechanisms that affect oxidative stress [43,44]. A high level of oxidative stress contributes to the death of tumor cells by necrosis or apoptosis [45,46].

As 2ʹ,7ʹ-dichlorodihydrofluorescein diacetate is only converted to its reduced form in living cells with an intact membrane, the decrease in ROS is probably due to a rather high level of necrosis, in the case of compound **5**, and the intensive formation of apoptotic bodies, in the case of compounds **14**, with an increase in the effective concentrations of these compounds.

Treatment with lapatinib and sorafenib caused a dose-dependent increase in ROS and decrease in the cell number that can be explained by the fact that only the remaining, living cells fluoresce at the basic ROS cell level.

### 3.4. Docking and Molecular Dynamics

We studied the inhibitory activity of the synthesized compounds in silico using a two-step protocol. Firstly, molecular docking was performed against various cancer-related protein targets. The obtained docking poses were energetically refined by molecular dynamics.

Thirty-three receptor structures were obtained from PDB. These structures included the following kinases: ABL (PDB ids: 3cs9, 2hyy, 3qrj), Trk (4at3, 5kvt, 6kzd), EGFR (4g5p), VEGFR (3hng, 4ag8, 3wze, 4asd), BRAF (3og7, 5hi2), ERBB (3pp0, 3bbt), LCK (2pl0), Aurora (2bfy, 2vrz, 4bm8, 4c2w, 5ew9), ROS (3zbf), ALK (4dce), MEK (4lmn), MET (1r0p), BTK (5kup), JAK (5toz), SYK (5y5u), Ret (6nec), Wee (2in6) and MAP (3gcs). Two receptors were poly(ADP-ribose)-polymerases (PDB ids: 4tvj, 7kk4). A total of 926 highest-ranked docking poses were obtained for each ligand–receptor pair.

The obtained docking poses were filtered using a −11.5 kcal/mol threshold to yield only high-affinity poses. The majority of these high-affinity poses, i.e., more than 75%, were the poses of isophthalic derivatives. Furthermore, type-2 protein kinases were the most common receptors in these poses, including Trkc kinase (PDB: 6kzd), the ABL family (PDB: 3cs9, 2hyy) and the VEGFR family (PDB: 3hng, 3wze, 4asd). The list of receptors is given in frequency-descending order. The analysis of amide bond substituents in the studied structures revealed that the docking-estimated binding affinities correlated with the presence or absence of 3-(trifluoromethyl)aniline in the ligand structure. Altogether, these observations strongly support the approach to the design of target phthalic acid derivatives as type-2 protein kinase inhibitors.

Poses with the highest docking scores, as well as some poses of particular interest to us regarding a certain structure, were subjected to binding affinity refinement using molecular dynamics. For 24 docking-generated complexes (18 with isophthalic linkers and 6 with terephthalic linkers), we obtained the binding energy using MM-PBSA (g_mmpbsa [47] tool) and MM-GBSA (gmx_mmpbsa [48]). We also used the machine learning rescoring model RF-Score-VS [49]. All the calculations were performed using snapshots of the MD trajectory. Known inhibitors were used as reference structures (Table 6). 

In our case, all three methods showed similar results. The MM-PBSA and MM-GBPSA binding energies were in close correlation with the linear correlation coefficient of (R^2^) 0.7. The RF-Score-VS scores were highly correlated with the van der Waals component of the MM-PBSA/GPBSA energies (R^2^ = 0.7) and not correlated with the electrostatic component (R^2^ = 0.1).

In general, the known inhibitors showed better binding energies than the studied phthalic acid derivatives. For ABL kinase, the second-generation inhibitor (nilotinib) was more potent in silico than the first-generation imatinib.

The results of the molecular modeling studies suggest that, for the studied structures, type-2 (imatinib-like) binding, rather than type-1 (lapatinib), is preferable. Furthermore, the obtained docking scores and MM-PBSA/MM-GBSA binding energies were, in general, higher for the isophthalic acid derivatives than their terephthalic counterparts. These observations correspond with the results of the in vitro testing, in which the phthalic derivatives showed better activities.

The structures of isophthalic derivatives **11** and **14** showed the best affinities. High binding energies and RF-Score-VS scores were observed for these complexes with ABL (3cs9) and VEGFR (3wze, 4ag8, 4asd), which are type-2 receptors. On the contrary, no activity of **14** was obtained against the type-2 receptor (3bbt).

**Figure 6:**
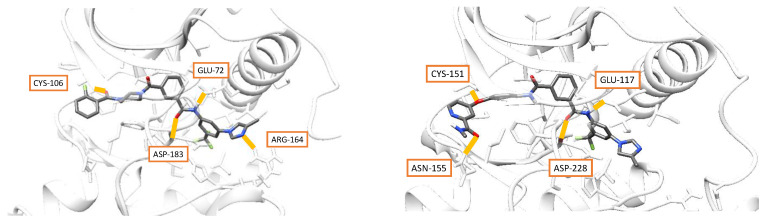
Binding models of **14** (left, PDB id: 3wze) and **11** (right, PDB id: 4asd) to VEGFR, predicted using molecular modeling. Hydrogen bonds are shown as orange lines [50].

The binding models of compounds **11** and **14** to the type-2 receptors, obtained by docking and MD simulations, are shown in Figure 6. These structures show a placement that is typical for type-2 inhibitors. Two hydrogen bonds form with asparagine and glutamine residues of the allosteric binding pocket. For structure **14**, the carbonyl group of phenyl(piperazin-1-yl)methanone may be involved in h-bond formation in the ATP site, while the 4-(4-aminophenoxy)-N-methylpicolinamide fragment of **11** may form two hydrogen bonds.

## 4. Conclusions

A series of novel compounds containing isophthalic and terephthalic diamides as linkers were designed as type-2 kinase inhibitors and synthesized via their corresponding phthalic dihalides. The screening of their cytotoxic actions against a panel of cell lines obtained from various types of tumors allowed us to establish that, in general, tumor cell cultures are more sensitive to isophthalic derivatives than to the terephthalic analogues. Compound **5** showed the greatest inhibitory activity against four cancer cell lines, K562, HL-60, MCF-7 and HepG2 (IC_50_ = 3.42, 7.04, 4.91 and 8.84 µM, respectively), in contrast to the terephthalic analogue **17**, which practically did not affect the cell lines’ viability. Isophthalic analogues **5** and **9** demonstrated high inhibitory activity against the PDGFRα and EGFR receptor protein kinases, with values of 73% and 90%, respectively. Possibly, the peculiarities of their structural geometry can explain the fact that the isophthalic derivatives showed higher activity compared to the terephthalic analogues. 

Compound **5** induced both early and late apoptosis. In addition, it showed a decrease in ROS and, at the same time, a decrease in the number of living cells, which was probably indirectly related to the rather high level of necrosis caused by the action of this compound on K562 cells.

Molecular docking and molecular dynamics studies showed that the obtained isophthalic derivatives could be type-2 inhibitors of ABL kinase and VEGFR, showing good docking scores and high MM-PBSA/GBSA binding energies compared to the known ligands. This research may offer a promising model for new anticancer drug development in the future.

## Figures and Tables

**Table 1:** Structures and yields of final compounds **5**–**28**.

R_1_	R_2_	Isophthalic Derivatives			Terephthalic Derivatives		
		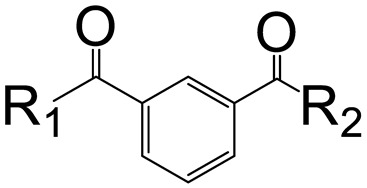			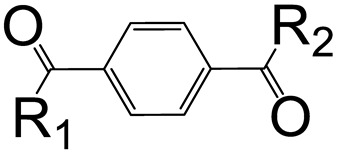		
		No.	Structure	Yield, %	No.	Structure	Yield, %
a	b	**5**	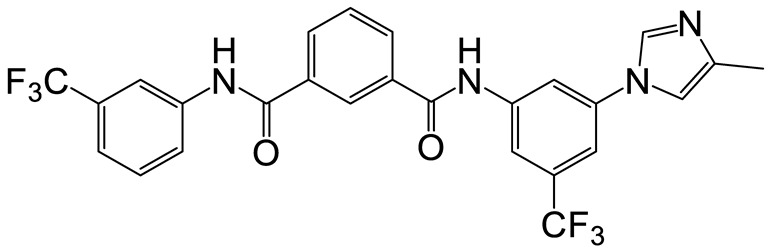	15	**17**	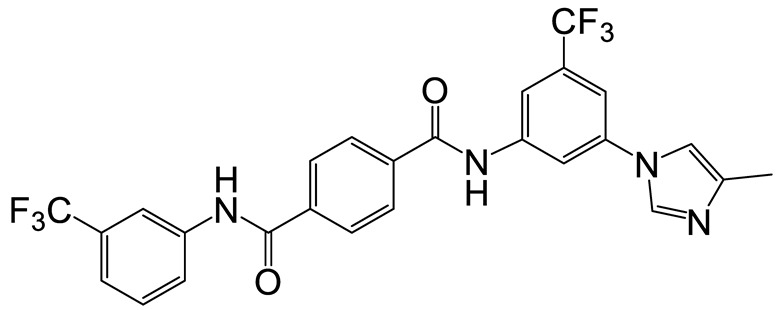	20
a	c	**6**	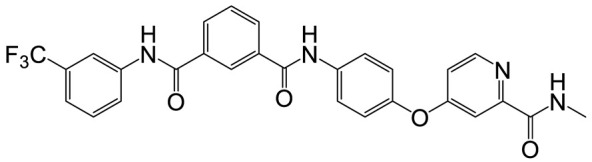	23	**18**	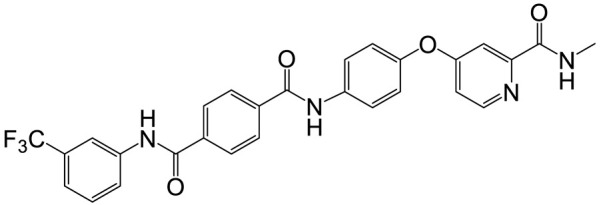	10
a	e	**7**	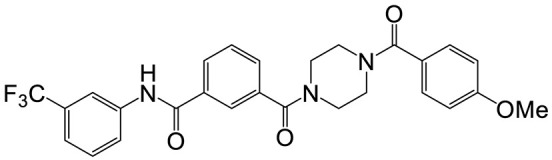	15	**19**	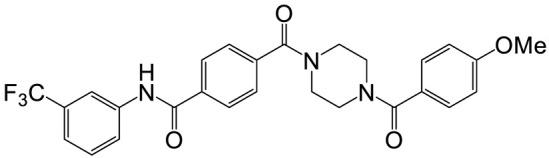	16
a	f	**8**	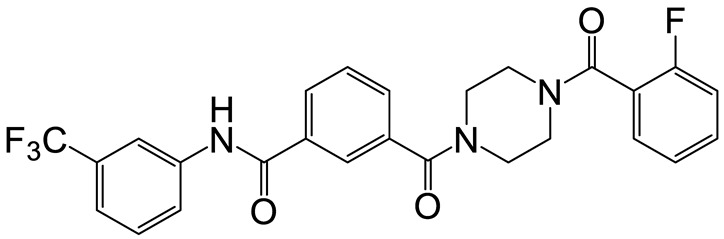	57	**20**	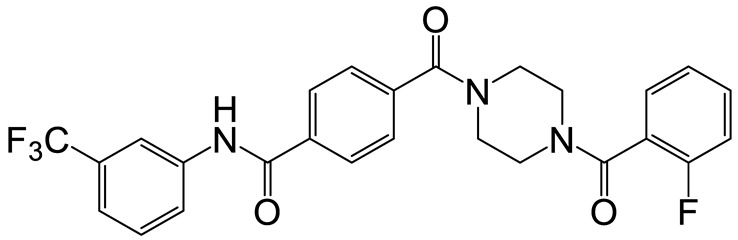	35
a	g	**9**	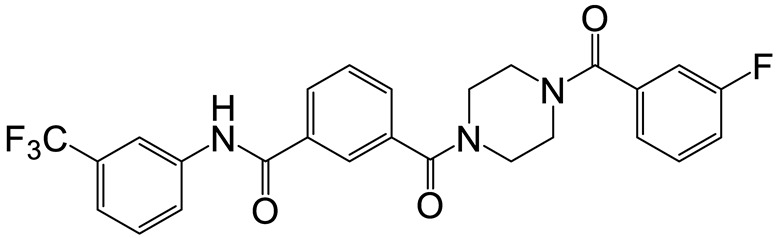	44	**21**	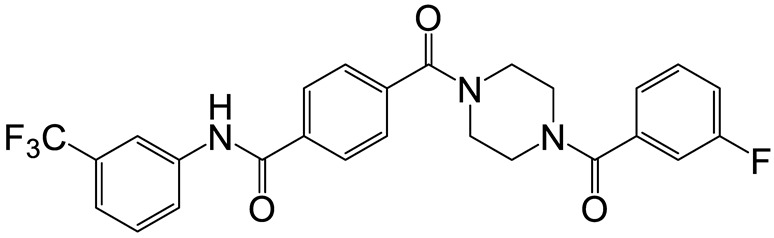	50
b	b	**10**	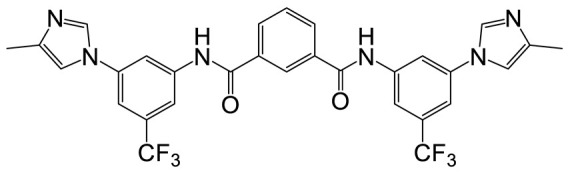	14	**22**	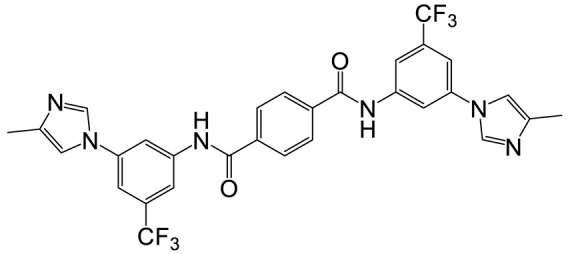	27
b	c	**11**	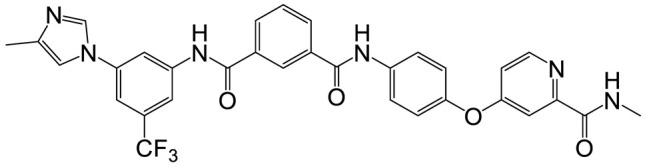	11	**23**	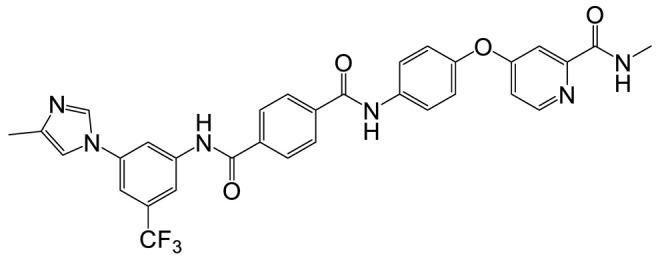	12
b	d	**12**	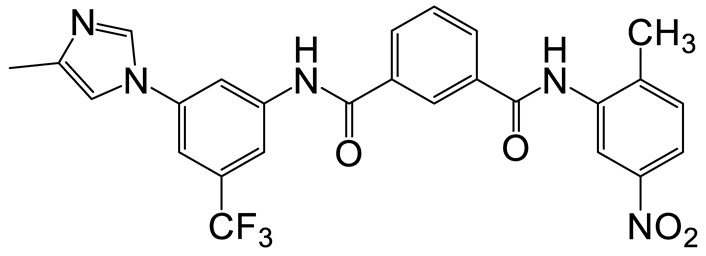	13	**24**	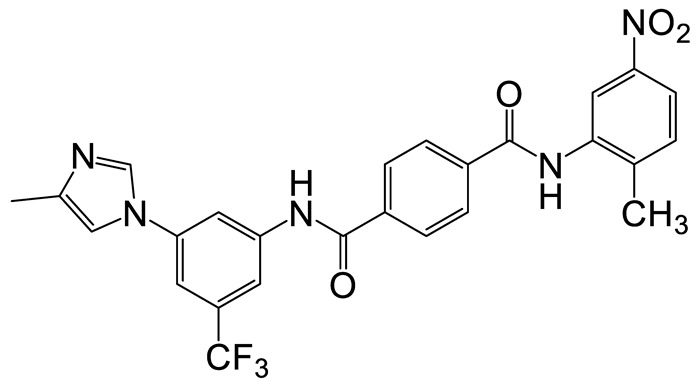	20
b	e	**13**	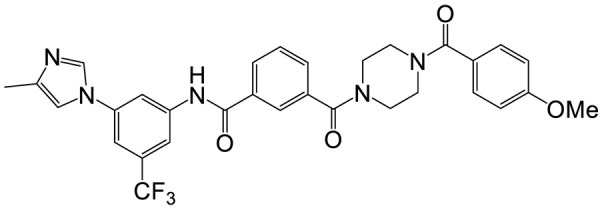	10	**25**	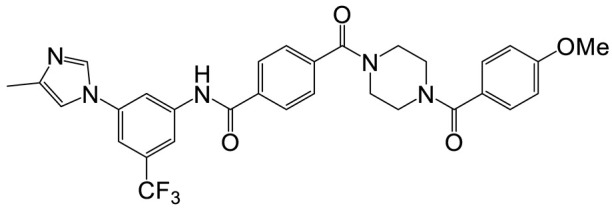	14
b	f	**14**	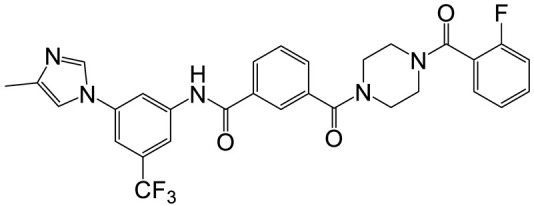	25	**26**	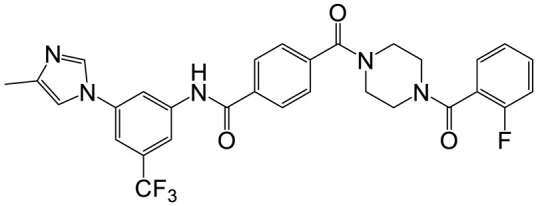	18
b	g	**15**	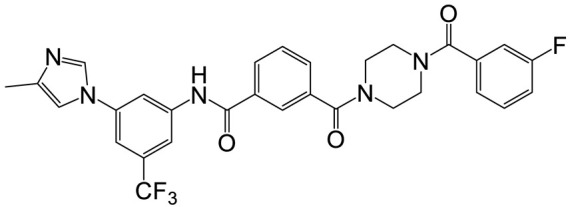	17	**27**	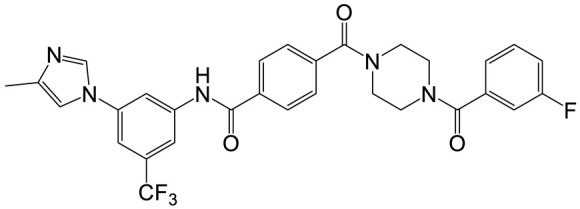	12
c	d	**16**	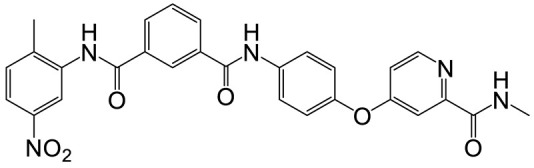	9	**28**	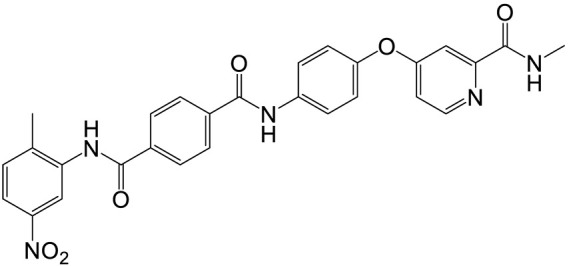	10

**Table 2:** In vitro antiproliferative activities of target compounds **5**, **6**, **8**–**12**, **14**, **15**, **17**–**22** and **24**–**26** against cancer cell lines (K562, HL-60, MCF-7, HepG2, A-549, OKP-GS) and normal human lymphoblastoid cells (RPMI 1788).

Comp.	Acid	R_1_	R_2_	Anti-Proliferative Activities in Deferent Cell Lines (IC_50_ µM) ^1^						
				K562	HL-60	MCF-7	HepG2	A549	OKP-GS	RPMI 1788
**5**	Iso	a	b	3.42 ± 0.78	7.04 ± 0.29	4.91 ± 1.27	7.84 ± 2.61	>50	17.76 ± 2.13	2.86 ± 0.09
**6**	Iso	a	c	>50	>50	41.15 ± 5.06	>50	>50	>50	12.98 ± 2.99
**8**	Iso	a	f	18.33 ± 1.15	18.19 ± 1.02	24.71 ± 5.95	35.49 ± 0.34	>50	>50	24.91 ± 1.17
**9**	Iso	a	g	20.99 ± 0.59	21.90 ± 1.59	26.29 ± 3.71	33.29 ± 3.45	>50	>50	26.26 ± 1.88
**10**	Iso	b	b	25.96 ± 2.38	35.75 ± 3.19	38.47 ± 1.95	23.21 ± 1.81	>50	13.23 ± 1.30	13.00 ± 0.30
**11**	Iso	b	c	>50	>50	>50	>50	>50	26.51 ± 3.65	21.89 ± 0.44
**12**	Iso	b	d	>50	>50	>50	>50	>50	27.47 ± 2.58	2.36 ± 1.13
**14**	Iso	b	f	7.71 ± 2.84	32.50 ± 1.50	27.30 ± 0.16	44.09 ± 4.19	>50	>50	26.63 ± 2.31
**15**	Iso	b	g	10.51 ± 2.54	34.76 ± 4.70	27.38 ± 0.26	45.81 ± 1.76	>50	>50	29.40 ± 1.79
**17**	Tere	a	b	15.62 ± 0.00	>50	>50	>50	>50	>50	>50
**18**	Tere	a	c	26.93 ± 0.00	>50	>50	45.85 ± 1.83	>50	34.43 ± 2.52	46.02 ± 1.76
**19**	Tere	a	e	>50	>50	>50	>50	>50	>50	>50
**20**	Tere	a	f	>50	>50	>50	>50	>50	>50	>50
**21**	Tere	a	g	>50	>50	>50	>50	>50	>50	>50
**22**	Tere	b	b	>50	>50	>50	>50	>50	>50	>50
**24**	Tere	b	d	16.98 ± 0.20	26.42 ± 3.35	15.48 ± 1.16	>50	>50	>50	36.49 ± 0.86
**26**	Tere	b	f	18.53 ± 0.73	26.41 ± 1.73	48.83 ± 1.17	44.28 ± 4.79	>50	>50	23.97 ± 0.03
**27**	Tere	b	g	22.25 ± 2.12	26.04 ± 4.15	49.36 ± 0.67	38.63 ± 1.11	>50	>50	24.08 ± 0.07
**Reference Compounds**										
Sorafenib				2.44 ± 0.21	3.37 ± 0.42	8.62 ± 2.24	5.01 ± 0.14	12.52 ± 1.64	7.17 ± 0.50	3.48 ± 0.39
Nilotinib				<0.1	22.67 ± 4.76	4.20 ± 0.14	4.81 ± 0.21	>50	2.76 ± 0.06	19.32 ± 0.09
Lapatinib				9.62 ± 1.56	10.58 ± 1.02	10.93 ± 0.34	27.10 ± 0.08	18.55 ± 2.65	7.32 ± 2.15	9.31 ± 0.90

^1^ IC_50_—the concentration that causes 50% cell growth inhibition after 72 h of exposition. The background color indicates the lowest IC_50_ values for compounds **5**, **14** and **15**, which were selected as candidates for additional studies.

**Table 3:** Inhibition of receptor tyrosine kinases by target compounds.

Kinase	% Inhibition at 10 µM										
	Lapat.	Soraf.	Sunit.	5	8	9	10	14	17	19	24
Tyrosine Kinases											
EGFR	100	0	-	0	14	90	1	21	0	4	2
HER2	76	0	-	33	0	64	19	14	13	39	49
HER4	100	0	-	0	0	37	0	0	0	2	3
IGF1R	0	0	-	0	0	0	0	0	0	0	0
InsR	0	0	-	0	6	0	0	0	0	17	0
KDR	24	100	-	18	34	33	33	26	0	16	0
PDGFRα	2	100	-	73	0	20	17	24	20	19	0
PDGFRβ	10	100	-	18	0	24	0	23	15	20	0
**Serine/Threonine Kinases**											
Aurora A	-	-	68	0	0	0	0	0	0	-	0
Aurora B	-	-	90	0	0	0	0	7	0	-	3

**Table 4:** Cell cycle data analysis of K562 cells treated with compound **5**, **14** and **15**.

Comp.	Concentration, µM	G1, %	S, %	G2/M, %	Apoptosis, %
K562 (control)		31.11	23.27	45.62	14.80
**5**	1	37.92	17.01	45.08	14.56
	5	31.77	18.14	50.09	37.20
	10	29.86	32.05	38.08	53.50
**14**	1	32.65	20.41	46.95	14.28
	5	27.58	17.25	55.16	25.10
	10	31.21	17.71	51.08	38.02
**15**	1	30.15	21.31	48.54	17.90
	5	34.76	11.59	53.65	20.54
	10	28.85	8.16	62.99	32.72
**Lap.**	1	34.47	13.31	52.23	14.74
	5	31.31	13.02	55.68	28.92
**Sor.**	1	36.50	7.72	55.78	22.60
	5	33.87	30.14	35.99	44.20

**Table 5:** Changes in ROS level under the influences of compounds **5**, **14** and **15**.

Concentration, µM	Control	ROS Level, % of Control				
		Compound				
		5	14	15	Sor.	Lap.
5	100%	76.76	92.84	92.89	69.20	64.12
10		64.66	76.96	87.87	79.03	72.23
20		62.90	71.90	94.07	71.59	89.07

**Table 6:** Calculated binding affinities of the studied and reference structures for their receptors.

Protein Family	PDB Code	Linker	N Comp.	Gmx_Mmpbsa, kcal/mol				RF- Score	g_mmpbsa, kJ/mol		
				v.d.w.	Electr.	Entr.	Total		v.d.w.	Electr.	Total
ABL	3cs9	iso	**7**	−68	−21.6	13.8	−46	6.1	−284.4	−43.96	−141.8
ABL	3cs9	iso	**8**	−62.6	−14	13.2	−41	6.2	−262	−29.21	−128.6
ABL	3cs9	iso	**11**	−77.1	−37.1	9.1	−47.9	6.3	−322.3	−77.15	−144.4
ABL	3cs9	iso	**14**	−72.6	−13.5	6	−53.3	6.3	−303.5	−28.01	−144.9
TRKC	6kzd	iso	**8**	−59.3	−2.4	13.3	−34.5	6.0	−248	−5.111	−123.5
TRKC	6kzd	iso	**9**	−48.5	−30.7	14.4	−21.5	6.0	−202.8	−64.39	−76.8
TRKC	6kzd	iso	**13**	−65.8	−40.7	17.8	−31.4	6.3	−275.3	−85.14	−95.59
TRKC	6kzd	iso	**14**	−62.7	−4.4	16.2	−31.4	6.1	−262.3	−9.205	−111.5
TRKC	6kzd	iso	**15**	−70.1	−20	9.9	−45.6	6.2	−293.4	−41.89	−137.9
TRKC	6kzd	tere	**21**	−58.1	−29.8	8.6	−39.3	6.0	−243.1	−62.39	−128.4
TRKC	6kzd	tere	**25**	−69.8	−22.4	14.2	−37	6.2	−291.8	−46.92	−149
TRKC	6kzd	tere	**26**	−69.6	−19	18.7	−38.8	6.2	−291.3	−39.37	−141.1
TRKC	6kzd	tere	**27**	−63.8	−5.5	15.2	−27.4	6.2	−266.7	−11.69	−125.9
VEGFR1	3hng	iso	**5**	−64.9	−22.6	16	−36.8	6.3	−271.3	−47.31	−119.6
VEGFR2	4ag8	iso	**5**	−65.4	−23.2	11.2	−44	6.2	−273.6	−49.12	−138.8
VEGFR2	4ag8	iso	**7**	−65.2	−33.1	7.7	−58	6.2	−272.9	−69.09	−148.4
VEGFR2	4ag8	iso	**11**	−76.4	−34.4	9.3	−53.5	6.5	−319.5	−71.87	−150.8
VEGFR2	3wze	iso	**11**	−71.1	−26.2	9.9	−48.8	6.5	−297.4	−54.7	−150.5
VEGFR2	4asd	iso	**11**	−79.9	−33.8	10.1	−53.6	6.5	−334.2	−70.75	−155.3
VEGFR2	3wze	iso	**14**	−74	−30.8	7.4	−64	6.5	−309.6	−64.57	−157.0
VEGFR2	3wze	tere	**21**	−51.9	−30.3	8	−34.7	6.0	−217	−63.19	−85.65
BRAF	5hi2	iso	**8**	−57.4	−27.4	7	−46.6	6.1	−240.2	−57.45	−118.9
ERBB2	3pp0	iso	**9**	−55.6	−9.1	7.7	−34.1	6.0	−232.6	−19.04	−96.72
ERBB2	3pp0	tere	**20**	−50.8	−15.6	11.5	−26.7	6.0	−212.6	−32.59	−86.55
ERBB4	3bbt	iso	**14**	−50.4	−22.4	8.6	−30.7	6.0	−211.0	−46.8	−79.0
	Reference ligands										
ABL	2hyy	imatinib		−67.4	−20.1	5.3	−50.5	6.2	−282.1	−42.4	−154.7
ABL	3cs9	nilotinib		−72.8	−28.1	6.1	−56.1	6.6	−304.6	−57.8	−183.1
VEGFR2	3wze	sorafenib		−60.8	−35.3	7.4	−58.5	6.2	−254.3	−73.9	−170.0
VEGFR2	4asd	sorafenib		−60.3	−37.9	5.9	−59.8	6.2	−252.2	−79.3	−153.0
ERBB4	3bbt	lapatinib		−58.7	−26.5	6.2	−54.4	6.1	−245.6	−55.2	−179.8
TRKC	6kzd	not approved		−82.6	−31.7	8.2	−61.0	6.4	−345.5	−66.2	−206.9

## Data Availability

Not applicable.

## Supplementary Materials

The following are available online at https://www.mdpi.com/article/10.3390/cimb45030117/s1.

## References

[1] Moen M.D., McKeage K., Plosker G.L., Siddiqui M.A.A. (2007). Imatinib: A review of its use in chronic myeloid leukaemia. Drugs.

[2] Riegel K., Vijayarangakannan P., Kechagioglou P., Bogucka K., Rajalingam K. (2022). Recent advances in targeting protein kinases and pseudokinases in cancer biology. Front. Cell Dev. Biol..

[3] Theivendren P., Kunjiappan S., Hegde Y.M., Vellaichamy S., Gopal M., Dhramalingam S.R., Kumar S., Singh R.K. (2021). Importance of Protein Kinase and Its Inhibitor: A Review. Protein Kinases.

[4] Bhatia R., Singh R.K., Singh R.K. (2021). Introductory chapter: Protein kinases as promising targets for drug design against cancer. Protein Kinases.

[5] Roskoski R. (2021). Properties of FDA-approved small molecule protein kinase inhibitors: A 2021 update. Pharmacol Res..

[6] Bajinting A., Ng H.L., Donev R. (2021). Chapter Nine—Structural studies of full-length receptor tyrosine kinases and their implications for drug design. Advances in Protein Chemistry and Structural Biology.

[7] Barouch-Bentov R., Sauer K. (2011). Mechanisms of drug resistance in kinases. Expert. Opin. Investig. Drugs.

[8] Soverini S., Rosti G., Iacobucci I., Baccarani M., Martinelli G. (2011). Choosing the best second-line tyrosine kinase inhibitor in imatinib-resistant chronic myeloid leukemia patients harboring Bcr-Abl kinase domain mutations: How reliable is the IC_50_?. Oncologist.

[9] Arter C., Trask L., Ward S., Yeoh S., Bayliss R. (2022). Structural features of the protein kinase domain and targeted binding by small-molecule inhibitors. J. Biol. Chem..

[10] Tuveson D.A., Willis N.A., Jacks T., Griffin J.D., Singer S., Fletcher C.D., Fletcher J.A., Demetri G.D. (2001). STI571 inactivation of the gastrointestinal stromal tumor c-KIT oncoprotein: Biological and clinical implications. Oncogene.

[11] Kitagawa D., Yokota K., Gouda M., Narumi Y., Ohmoto H., Nishiwaki E., Akita K., Kirii Y. (2013). Activity-based kinase profiling of approved tyrosine kinase inhibitors. Genes Cells.

[12] Voigtlaender M., Schneider-Merck T., Trepel M. (2018). Lapatinib. Recent Results Cancer Res..

[13] Papaetis G.S., Syrigos K.N. (2009). Sunitinib: A multitargeted receptor tyrosine kinase inhibitor in the era of molecular cancer therapies. BioDrugs.

[14] Escudier B., Worden F., Kudo M. (2019). Sorafenib: Key lessons from over 10 years of experience. Expert Rev. Anticancer Ther..

[15] Takeda T., Tsubaki M., Kato N., Genno S., Ichimura E., Enomoto A., Imano M., Satou T., Nishida S. (2021). Sorafenib treatment of metastatic melanoma with c Kit aberration reduces tumor growth and promotes survival. Oncol. Lett..

[16] Aparicio-Gallego G., Blanco M., Figueroa A., García-Campelo R., Valladares-Ayerbes M., Grande-Pulido E., Antón-Aparicio L. (2011). New insights into molecular mechanisms of sunitinib-associated side effects. Mol. Cancer Ther..

[17] Schult C., Dahlhaus M., Ruck S., Sawitzky M., Amoroso F., Lange S., Etro D., Glass A., Fuellen G., Boldt S. (2010). The multikinase inhibitor Sorafenib displays significant antiproliferative effects and induces apoptosis via caspase 3, 7 and PARP in B- and T-lymphoblastic cells. BMC Cancer.

[18] Kharaziha P., Chioureas D., Baltatzis G., Fonseca P., Rodriguez P., Gogvadze V., Lennartsson L., Björklund A.C., Zhivotovsky B., Grandér D. (2015). Sorafenib-induced defective autophagy promotes cell death by necroptosis. Oncotarget.

[19] Yang Q., Gao L., Huang X., Weng J., Chen Y., Lin S., Yin Q. (2021). Sorafenib prevents the proliferation and induces the apoptosis of liver cancer cells by regulating autophagy and hypoxia-inducible factor-1. Exp. Ther. Med..

[20] Blackhall F., Cappuzzo F. (2016). Crizotinib: From discovery to accelerated development to front-line treatment. Ann Oncol..

[21] Elnair R., Galal A. (2018). Finding the right BCR-ABL1 tyrosine kinase inhibitor: A case report of successful treatment of a patient with chronic myeloid leukemia and a V299L mutation using nilotinib. BMC Cancer.

[22] Tadesse F., Asres G., Abubeker A., Gebremedhin A., Radich J. (2021). Spectrum of BCR-ABL Mutations and Treatment Outcomes in Ethiopian Imatinib-Resistant Patients with Chronic Myeloid Leukemia. JCO Glob. Oncol..

[23] Li W., Feng C., Di W., Hong S., Chen H., Ejaz M., Yang Y., Xu T.R. (2020). Clinical use of vascular endothelial growth factor receptor inhibitors for the treatment of renal cell carcinoma. Eur. J. Med. Chem..

[24] Epaillard N., Simonaggio A., Elaidi R., Azzouz F., Braychenko E., Thibault C., Sun C.M., Moreira M., Oudard S., Vano Y.A. (2020). BIONIKK: A phase 2 biomarker driven trial with nivolumab and ipilimumab or VEGFR tyrosine kinase inhibitor (TKI) in naïve metastatic kidney cancer. Bull. Cancer.

[25] Killock D. (2020). Biomarkers 101—Personalizing therapy for RCC. Nat. Rev. Clin. Oncol..

[26] D’Avella C., Abbosh P., Pal S.K., Geynisman D.M. (2020). Mutations in renal cell carcinoma. Urol. Oncol..

[27] Davis M.I., Hunt J.P., Herrgard S., Ciceri P., Wodicka L.M., Pallares G., Hocker M., Treiber D.K., Zarrinkar P.P. (2011). Comprehensive analysis of kinase inhibitor selectivity. Nat. Biotechnol..

[28] Wood E.R., Truesdale A.T., McDonald O.B., Yuan D., Hassell A., Dickerson S.H., Ellis B., Pennisi C., Horne E., Lackey K. (2004). A unique structure for epidermal growth factor receptor bound to GW572016 (Lapatinib): Relationships among protein conformation, inhibitor off-rate, and receptor activity in tumor cells. Cancer Res..

[29] Xie Z., Yang X., Duan Y., Han J., Liao C. (2021). Small-Molecule Kinase Inhibitors for the Treatment of Nononcologic Diseases. J. Med. Chem..

[30] Faryna A.V., Kalinichenko E.N. (2019). Computer-aided molecular design of new potential inhibitors of protein kinases using of 4-methyl-benzoic acid as a linker. J. Comput. Chem. Mol. Model..

[31] Kalinichenko E., Faryna A., Kondrateva V., Vlasova A., Shevchenko V., Melnik A., Avdoshko O., Belko A. (2019). Synthesis, biological activities and docking studies of novel 4-(arylaminomethyl)benzamide derivatives as potential tyrosine kinase inhibitors. Molecules.

[32] Kalinichenko E., Faryna A., Bozhok T., Panibrat A. (2021). Synthesis, In Vitro and In Silico Anticancer Activity of New 4-Methylbenzamide Derivatives Containing 2,6-Substituted Purines as Potential Protein Kinases Inhibitors. Int. J. Mol. Sci..

[33] Alhossary A., Handoko S.D., Mu Y., Kwoh C.K. (2015). Fast, accurate, and reliable molecular docking with QuickVina 2. Bioinformatics.

[34] NCI Chemical Identifier Resolver. http://cactus.nci.nih.gov/chemical/structure.

[35] Huang W., Shakespeare W.C. (2007). An Efficient Synthesis of Nilotinib (AMN107). Synthesis.

[36] Wei Y., To K.K.W., Au-Yeng S.C.F. (2015). Synergistic cytotoxicity from combination of imatinib and platinum-based anticancer drugs specifically in Bcr-Abl positive leukemia cells. J. Pharmacol. Sci..

[37] Fabarius A., Giehl M., Rebacz B., Krämer A., Frank O., Haferlach C., Duesberg P., Hehlmann R., Seifarth W., Hochhaus A. (2008). Centrosome aberrations and G1 phase arrest after in vitro and in vivo treatment with the SRC/ABL inhibitor dasatinib. Haematologica.

[38] Sauzay C., Louandre C., Bodeau S., Anglade F., Godin C., Saidak Z., Fontaine L.X., Usureau C., Martin N., Molinie R. (2018). Protein biosynthesis, a target of sorafenib, interferes with the unfolded protein response (UPR) and ferroptosis in hepatocellular carcinoma cells. Oncotarget.

[39] Riccardi C., Nicoletti I. (2006). Analysis of apoptosis by propidium iodide staining and flow cytometry. Nat. Protoc..

[40] Yao M., Shang Y.Y., Zhou Z.W., Yang Y.X., Wu Y.S., Guan L.F., Wang X.Y., Zhou S.F., Wei X. (2017). The research on lapatinib in autophagy, cell cycle arrest and epithelial to mesenchymal transition via Wnt/ErK/PI3K-AKT signaling pathway in human cutaneous squamous cell carcinoma. J. Cancer.

[41] Rieger A.M., Nelson K.L., Konowalchuk J.D., Barreda D.R. (2011). Modified annexin V/propidium iodide apoptosis assay for accurate assessment of cell death. J. Vis. Exp..

[42] Eruslanov E., Kusmartsev S. (2010). Identification of ROS using oxidized DCFDA and flow-cytometry. Methods Mol. Biol..

[43] Azad M.B., Chen Y., Gibson S.B. (2009). Regulation of autophagy by reactive oxygen species (ROS): Implications for cancer progression and treatment. Antioxid. Redox Signal.

[44] Circu M.L., Aw T.Y. (2010). Reactive oxygen species, cellular redox systems, and apoptosis. Free Radic. Biol. Med..

[45] Matés J.M., Sánchez-Jiménez F.M. (2000). Role of reactive oxygen species in apoptosis: Implications for cancer therapy. Int. J. Biochem. Cell Biol..

[46] Castaldo S.A., Freitas J.R., Conchinha N.V., Madureira P.A. (2016). The Tumorigenic Roles of the Cellular REDOX Regulatory Systems. Oxid. Med. Cell Longev..

[47] Kumari R., Kumar R., Lynn A., Open Source Drug Discovery Consortium (2014). g_mmpbsa—A GROMACS Tool for High-Throughput MM-PBSA Calculations. J. Chem. Inf. Model..

[48] Valdés-Tresanco M.S., Valdés-Tresanco M.E., Valiente P.A., Moreno E. (2021). Gmx_mmpbsa: A new tool to perform end-state free energy calculations with gromacs. J. Chem. Theory Comput..

[49] Wójcikowski M., Ballester P.J., Siedlecki P. (2017). Performance of machine-learning scoring functions in structure-based virtual screening. Sci. Rep..

[50] Faryna A., Kalinichenko E., Erman Salih I. (2022). N1-(3-(Trifluoromethyl)Phenyl) Isophthalamide Derivatives as Promising Inhibitors of Vascular Endothelial Growth Factor Receptor: Pharmacophore-Based Design, Docking, and MM-PBSA/MM-GBSA Binding Energy Estimation. Molecular Docking-Recent Advances.

