# Supplementary material for: Novel Phthalic-Based Anticancer Tyrosine Kinase Inhibitors: Design, Synthesis and Biological Activity
Source: Curr Issues Mol Biol. 2023 Feb 22;45(3):1820–42. doi: 10.3390/cimb45030117 (PMC10046946; doi:10.3390/cimb45030117)

^{1}H, ^{13}C and ^{19}F NMR spectra of compound **5** (DMSO-d6),

N1-(3-(4-methyl-1H-imidazol-1-yl)-5-(trifluoromethyl)phenyl)-N3-(3-(trifluoromethyl)phenyl)-isophthalamide

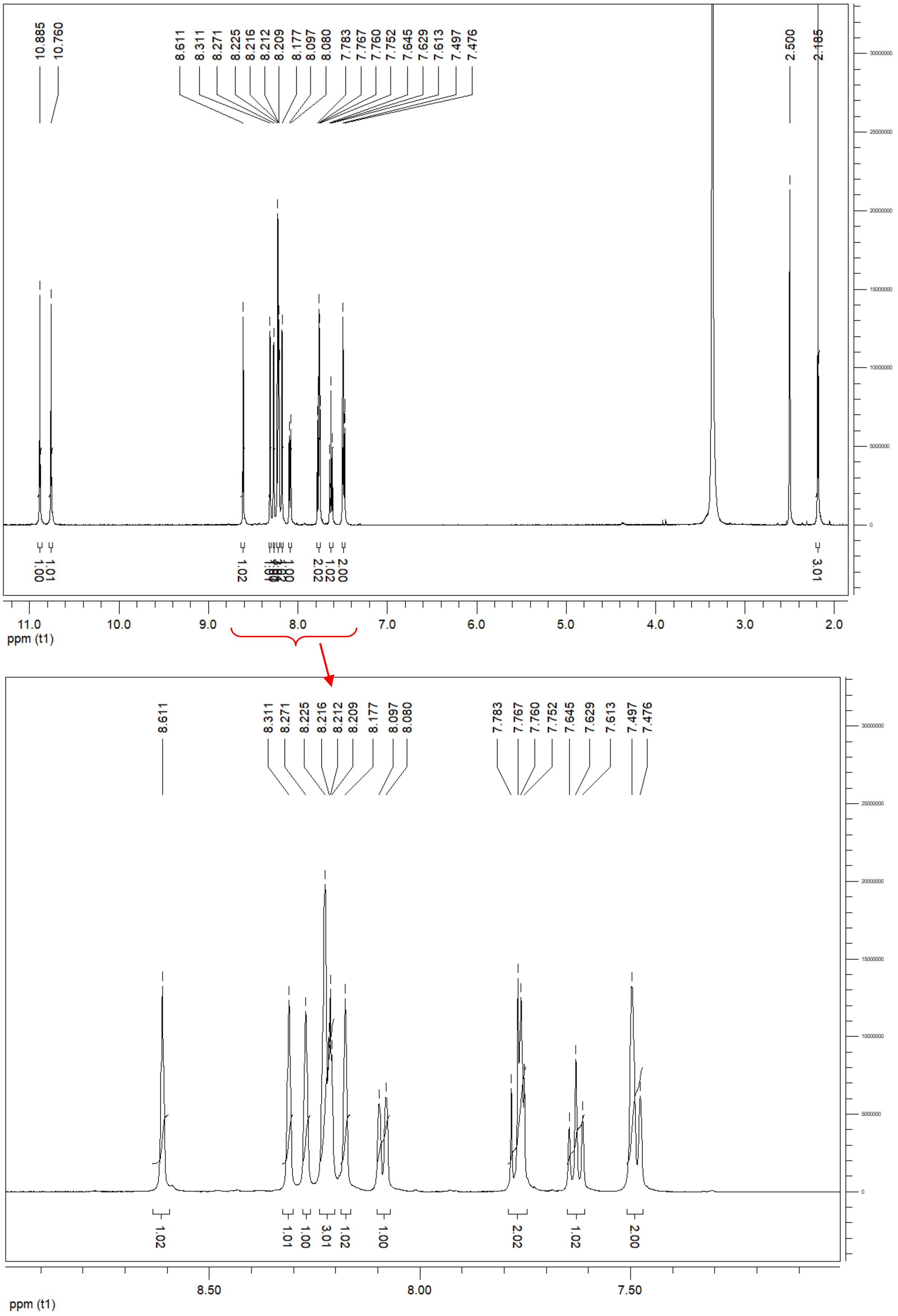

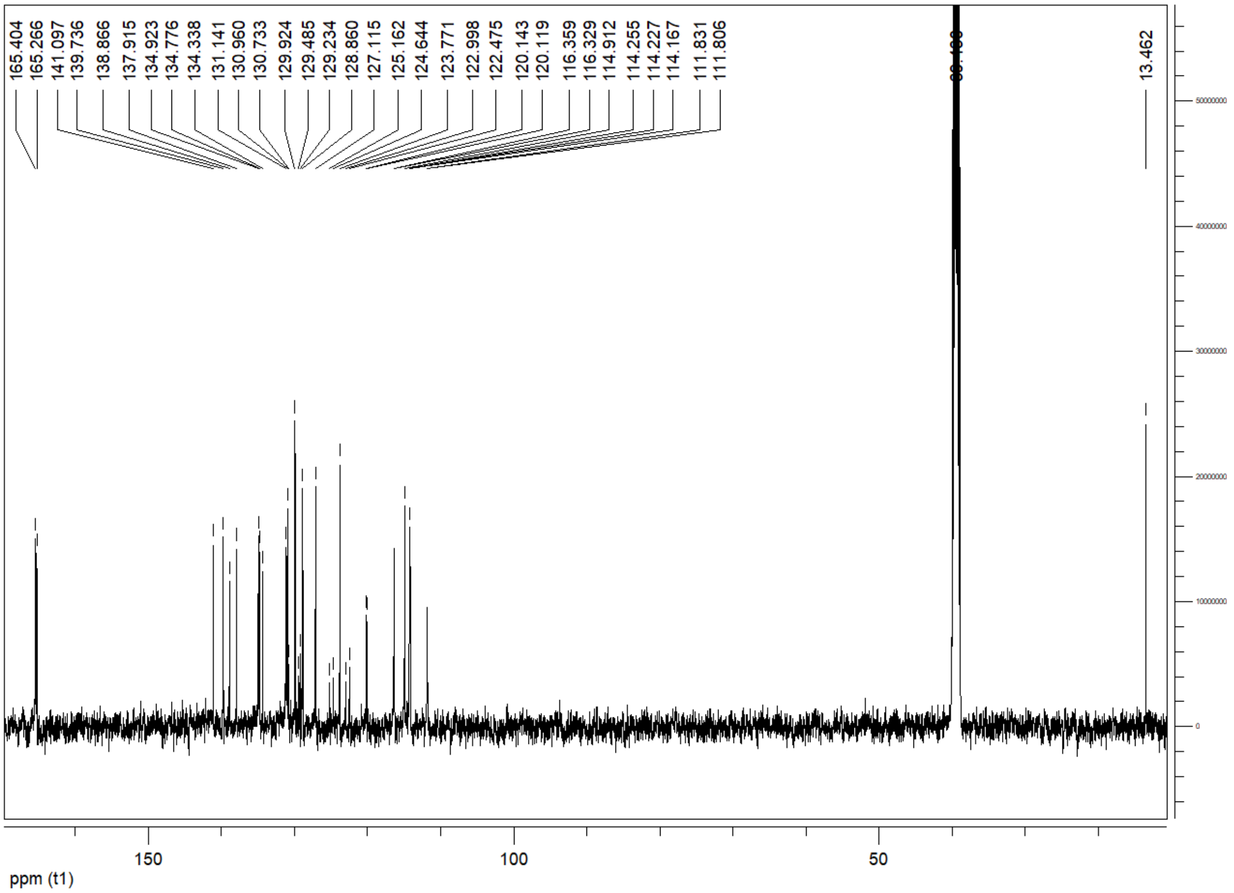
165.404
165.266
141.097
139.736
138.866
137.915
134.923
134.776
134.338
131.141
130.960
130.733
129.924
129.485
129.234
128.860
127.115
125.162
124.644
123.771
122.998
122.475
120.143
120.119
116.359
116.329
114.912
114.255
114.227
114.167
111.831
111.806
13.462
150
100
50
ppm (t1)

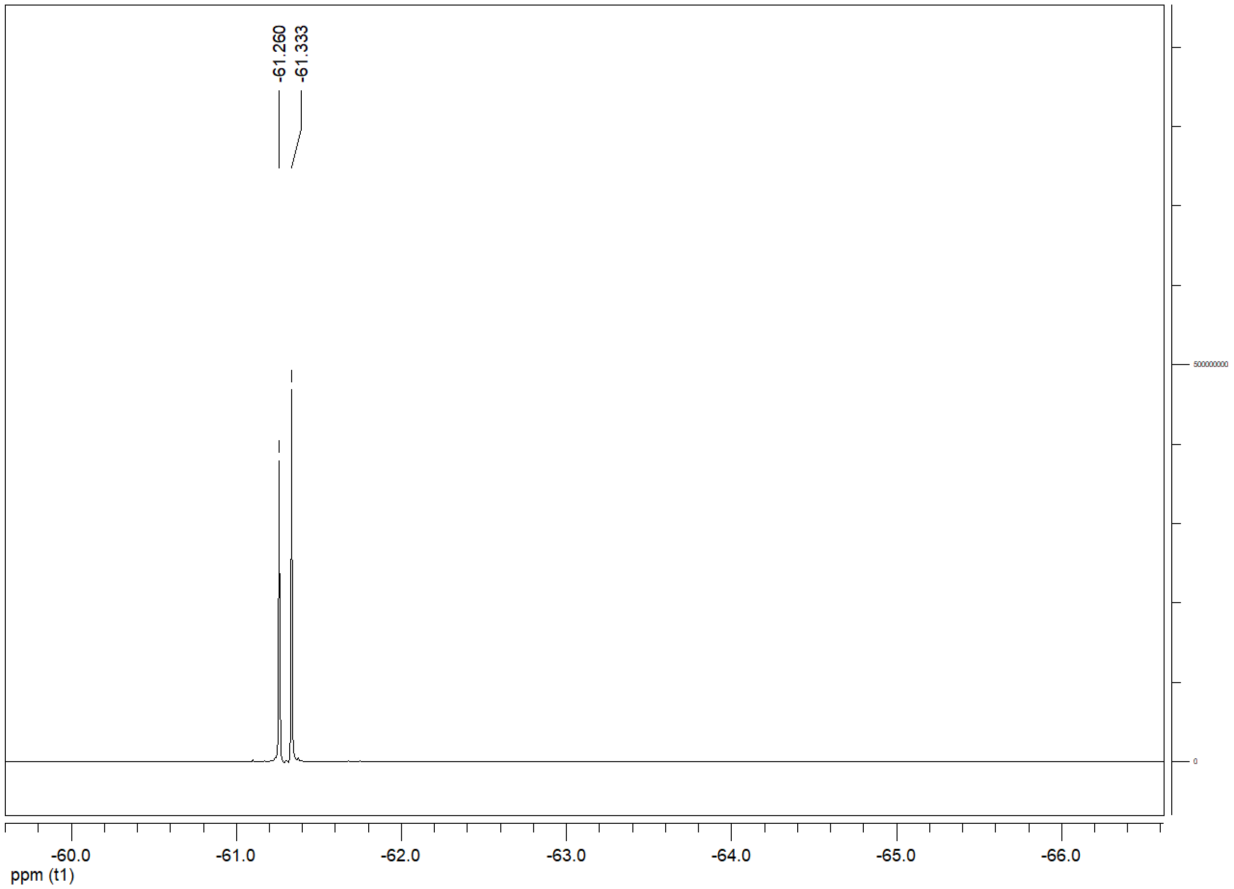
-61.260
-61.333
-60.0
-61.0
-62.0
-63.0
-64.0
-65.0
-66.0
ppm (t1)

^{1}H, ^{13}C and ^{19}F NMR spectra of compound **6** (DMSO-d6),

N1-(4-((2-(methylcarbamoyl)pyridin-4-yl)oxy)phenyl)-N3-(3-(trifluoromethyl)phenyl)isophthalamide

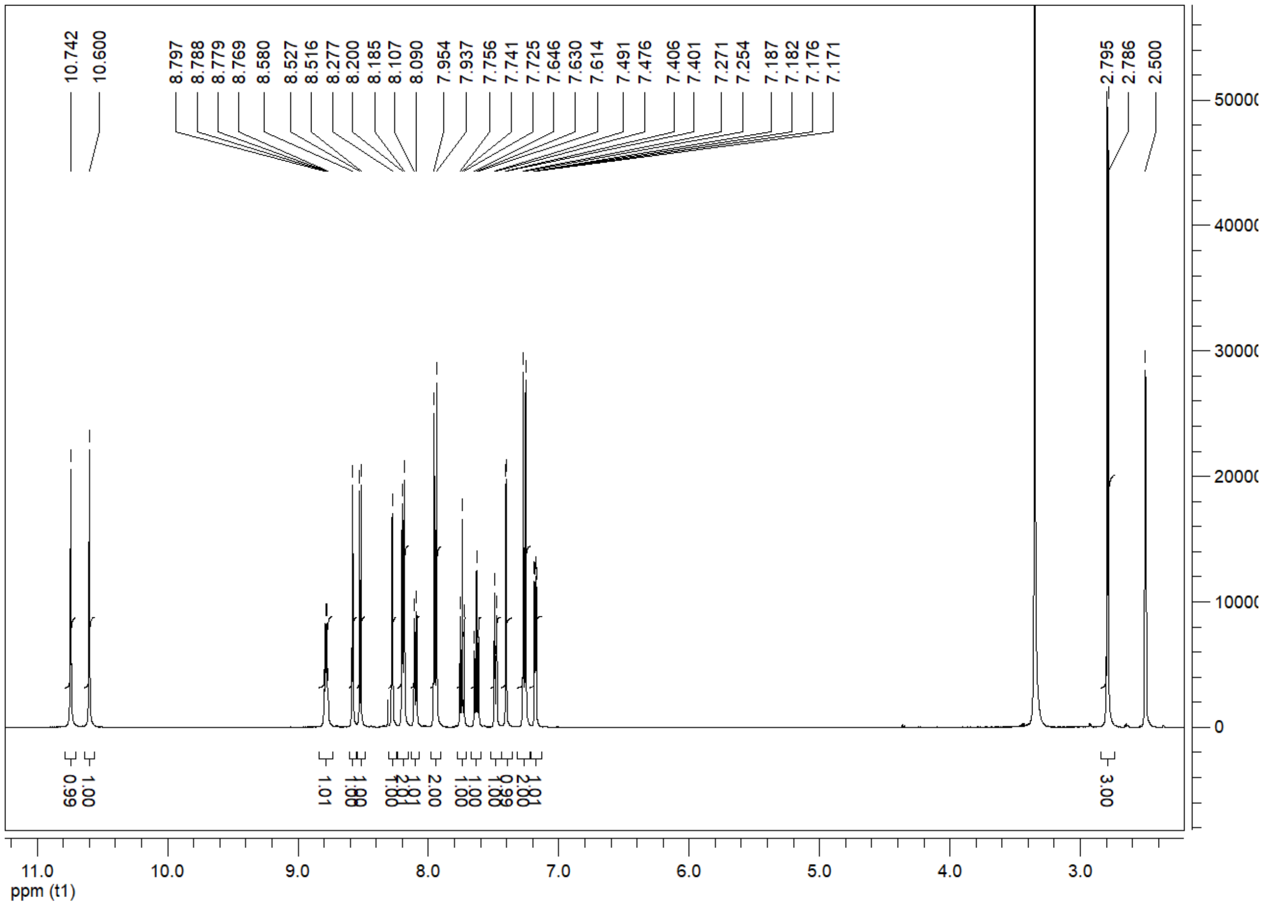

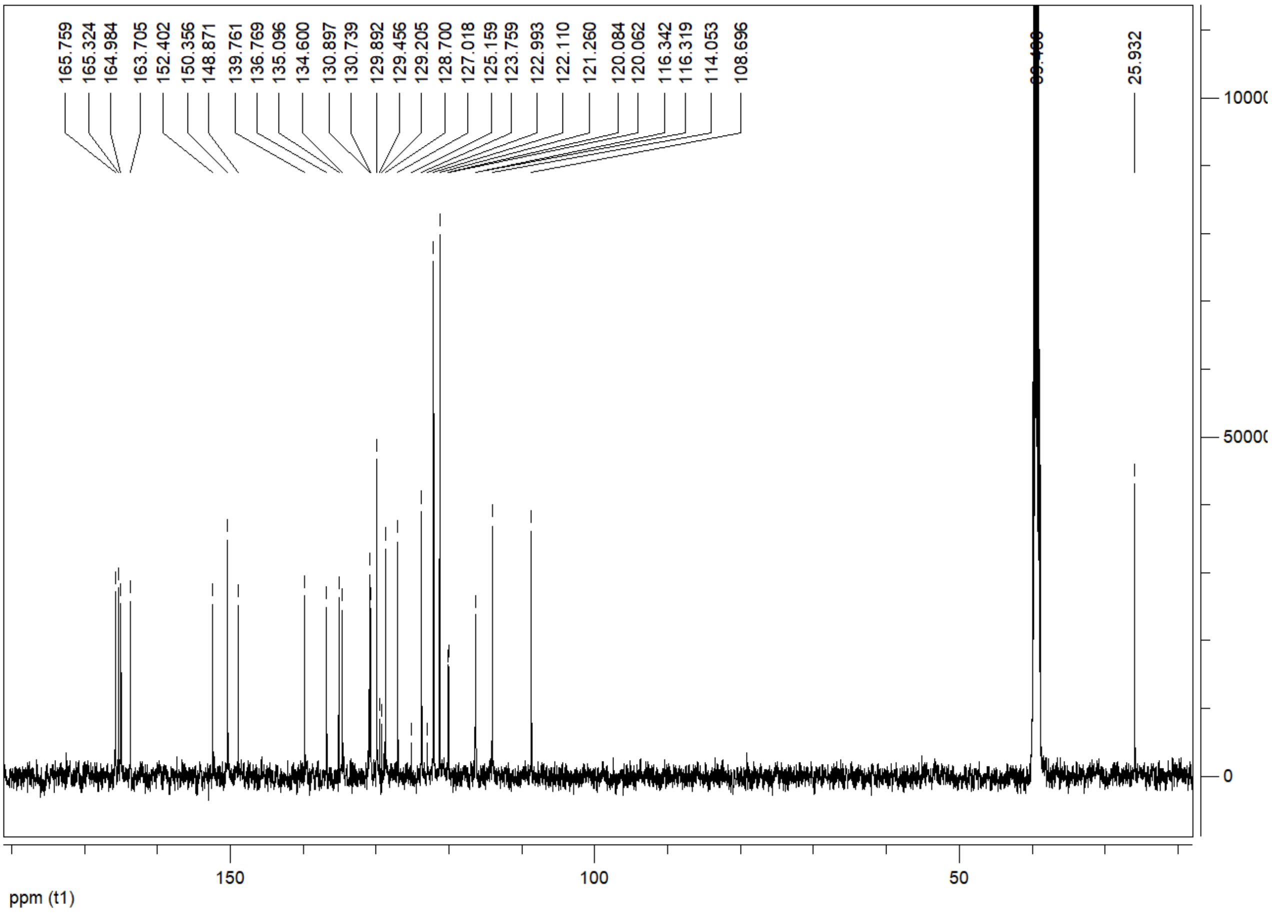

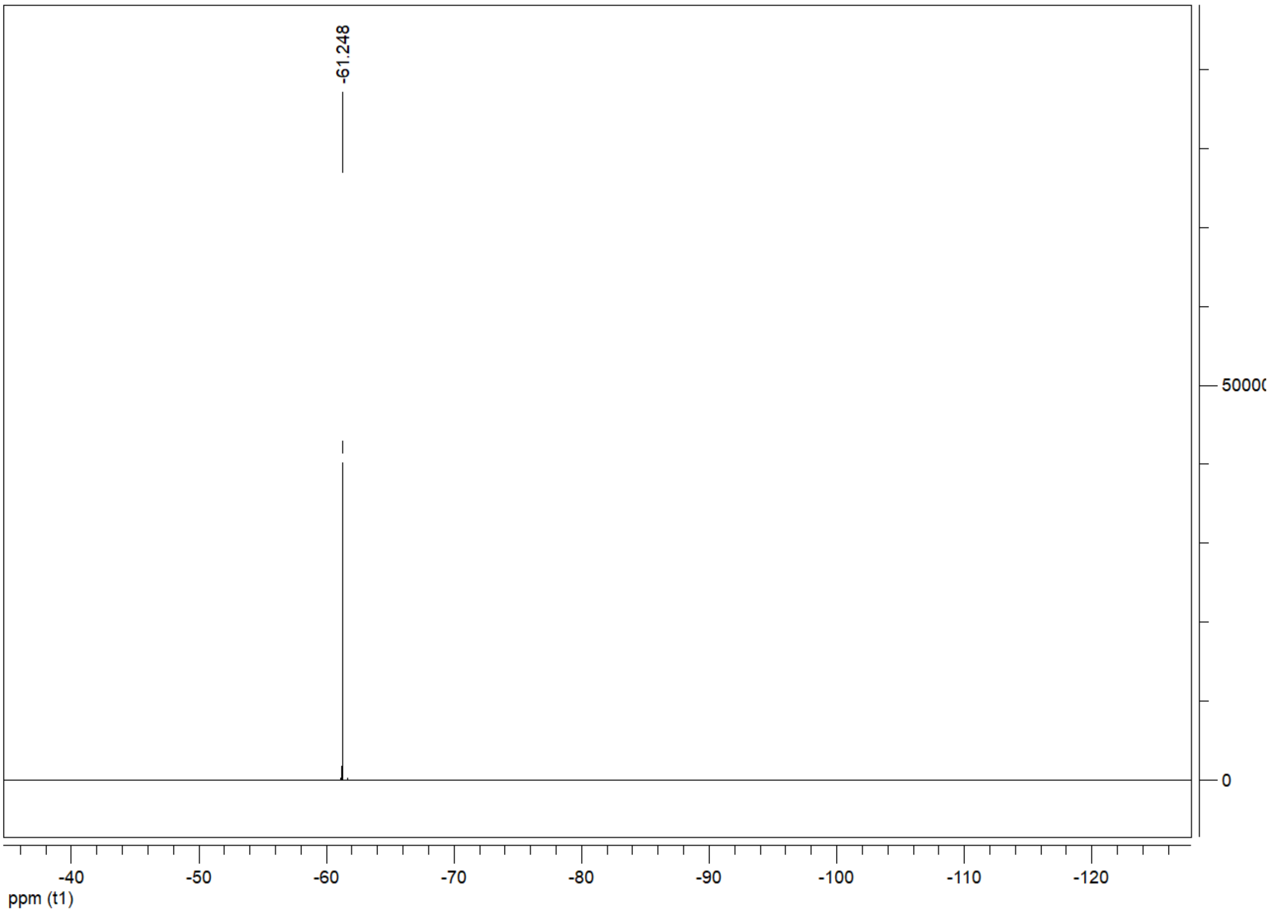

^{1}H, ^{13}C and ^{19}F NMR spectra of compound **7** (DMSO-d6),

3-(4-(4-Methoxybenzoyl)piperazine-1-carbonyl)-N-(3-(trifluoromethyl)phenyl)benzamide

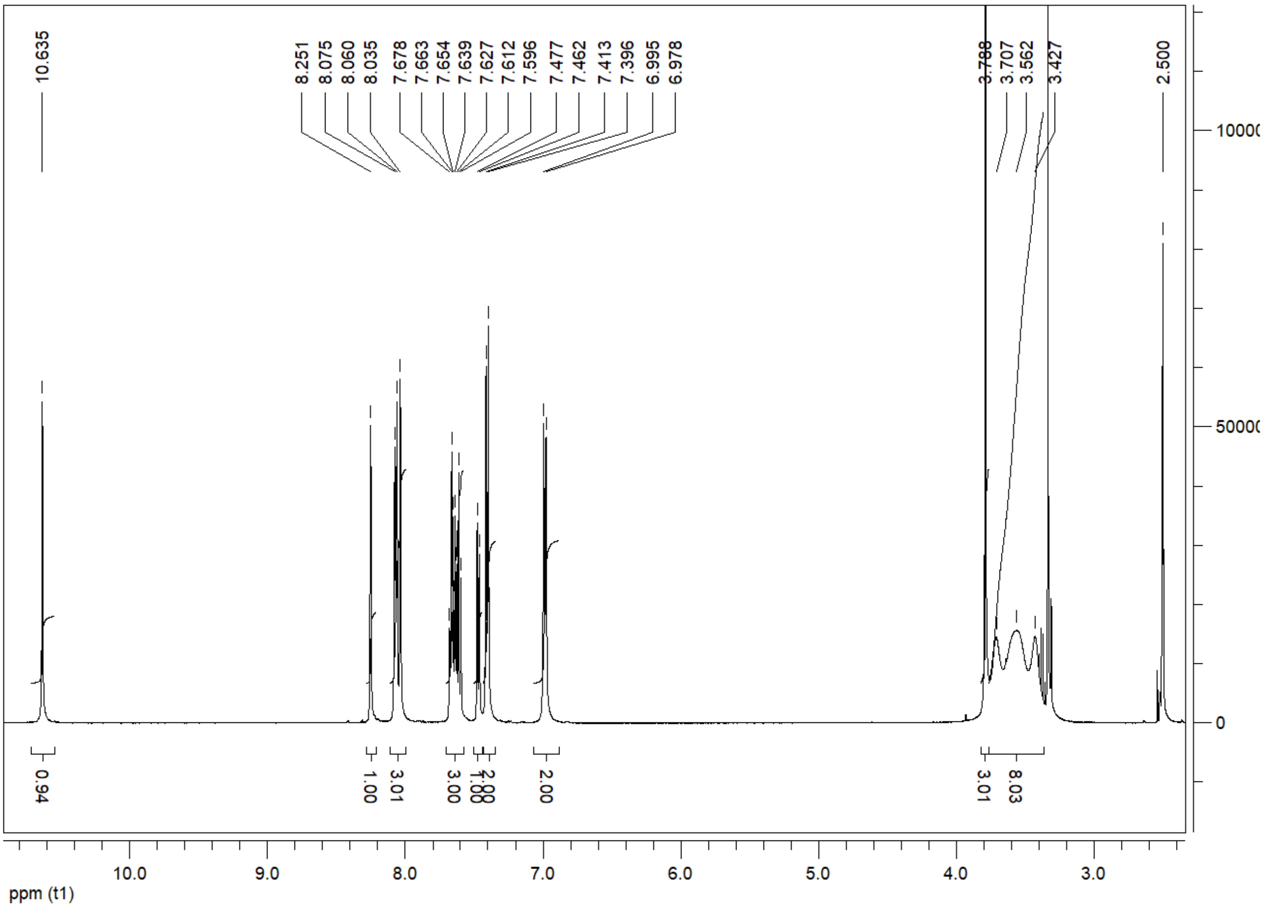

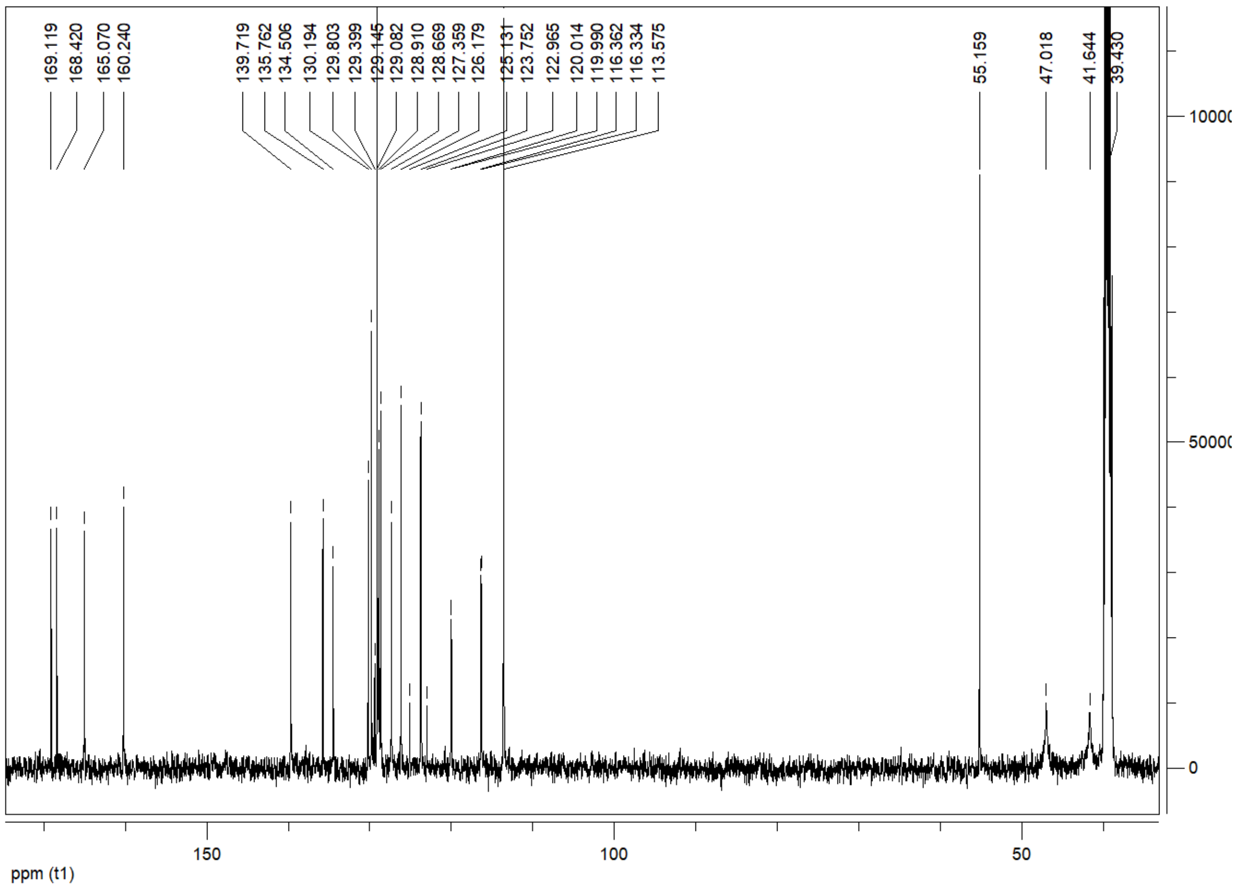
169.119
168.420
165.070
160.240
139.719
135.762
134.506
130.194
129.803
129.399
129.145
129.082
128.910
128.669
127.359
126.179
125.131
123.752
122.965
120.014
119.990
116.362
116.334
113.575
55.159
47.018
41.644
39.430
ppm (t1)

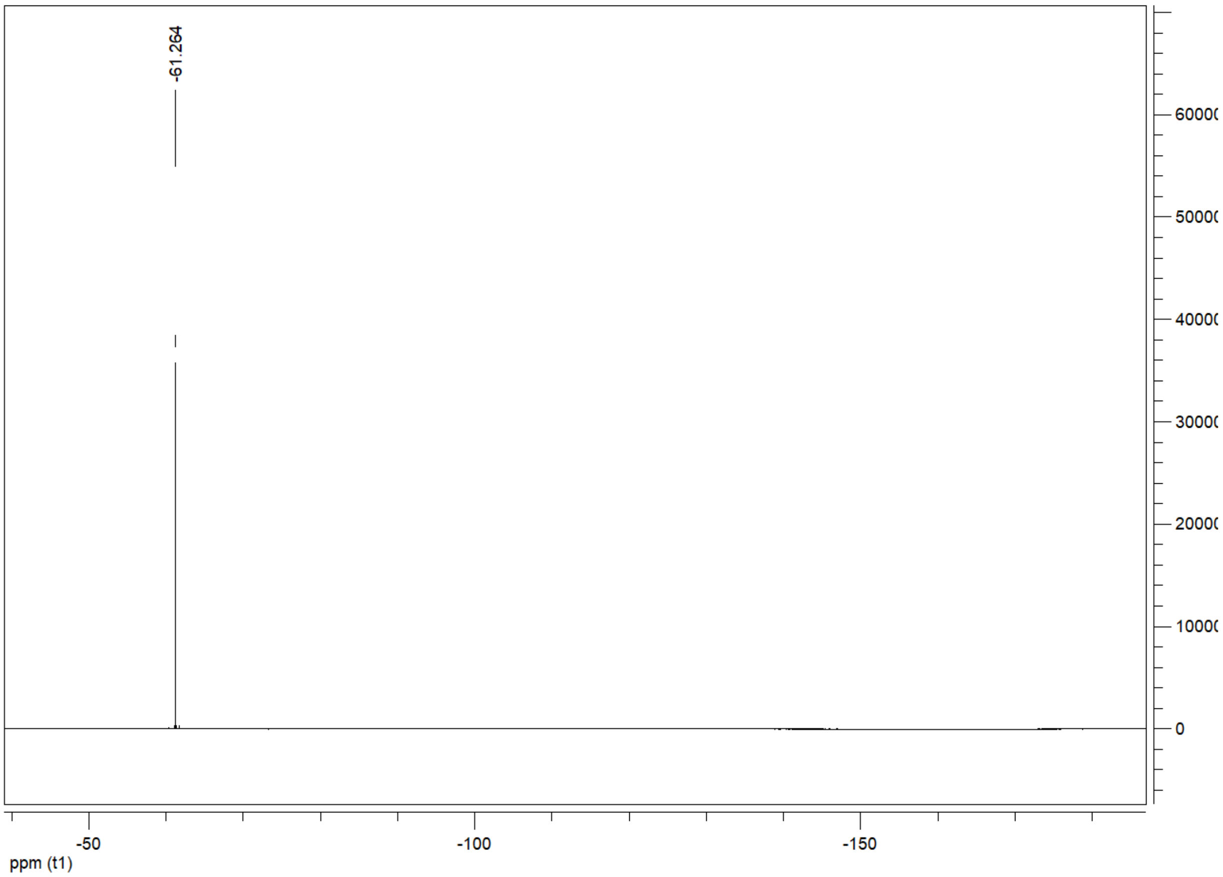
-61.264
ppm (t1)

^{1}H, ^{13}C and ^{19}F NMR spectra of compound **8** (DMSO-d6),

3-(4-(2-Fluorobenzoyl)piperazine-1-carbonyl)-N-(3-(trifluoromethyl)phenyl)benzamide

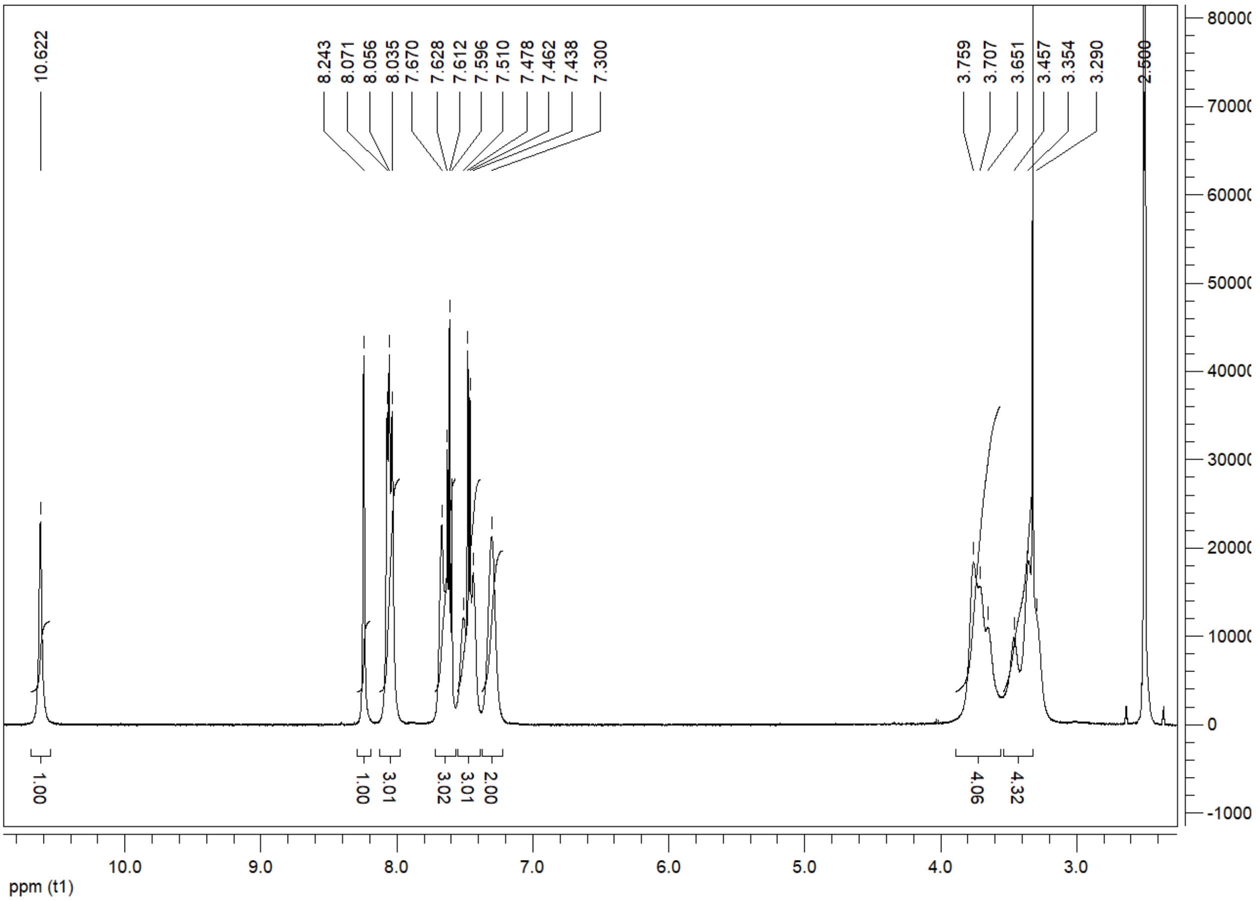

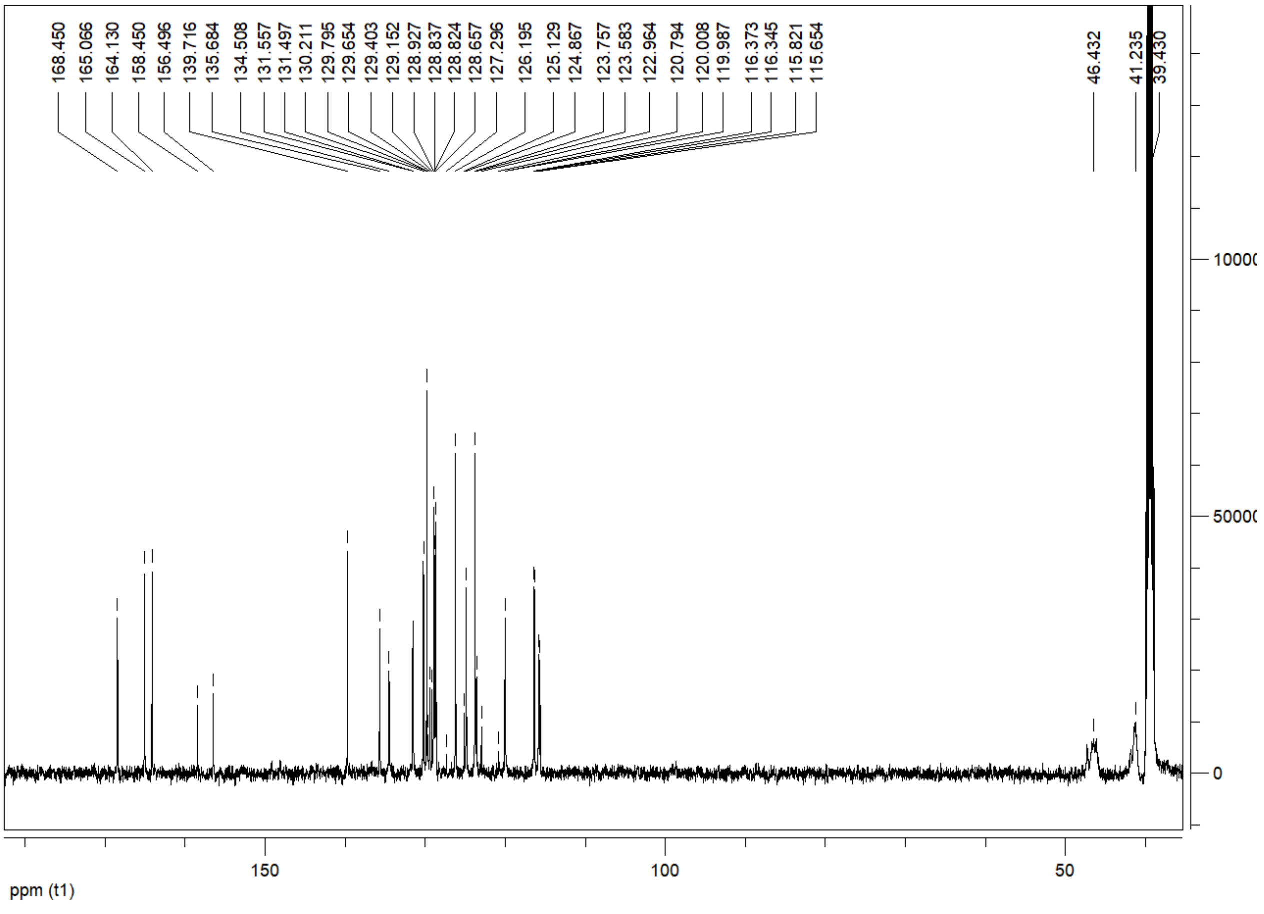

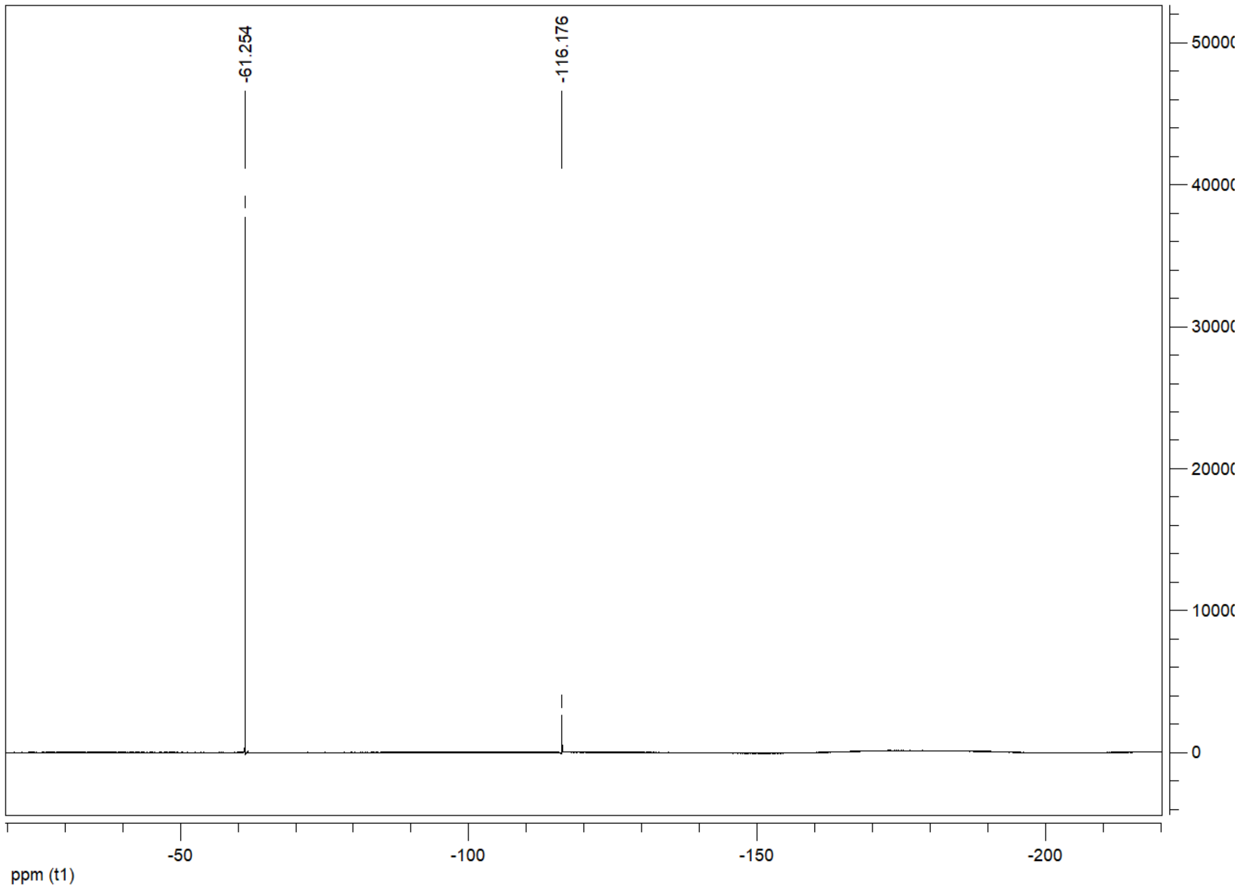

^{1}H, ^{13}C and ^{19}F NMR spectra of compound **9** (DMSO-d6),

3-(4-(3-Fluorobenzoyl)piperazine-1-carbonyl)-N-(3-(trifluoromethyl)phenyl)benzamide

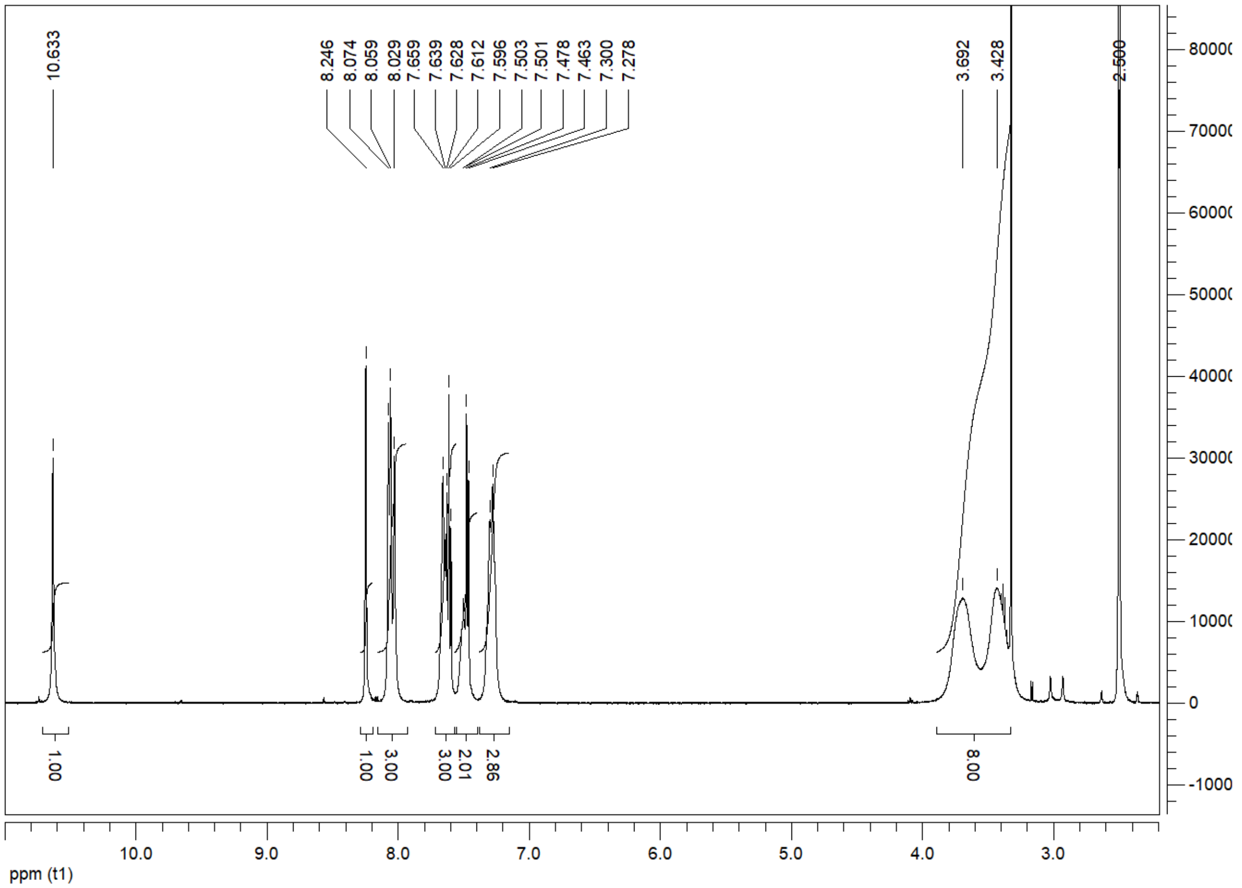

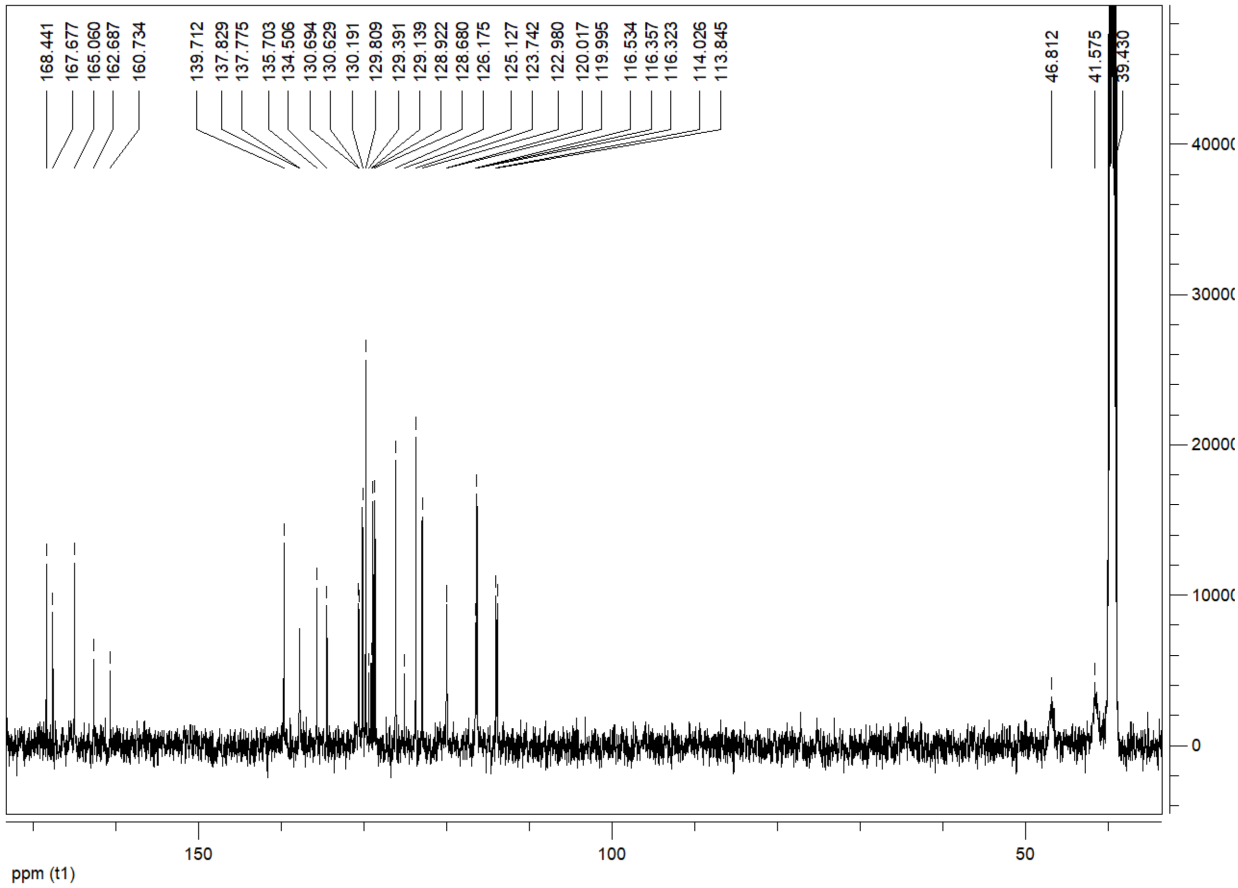
168.441
167.677
165.060
162.687
160.734
139.712
137.829
137.775
135.703
134.506
130.694
130.629
130.191
129.809
129.391
129.139
128.922
128.680
126.175
125.127
123.742
122.980
120.017
119.995
116.534
116.357
116.323
114.026
113.845
46.812
41.575
39.430
150
100
50
ppm (t1)
0
10000
20000
30000
40000

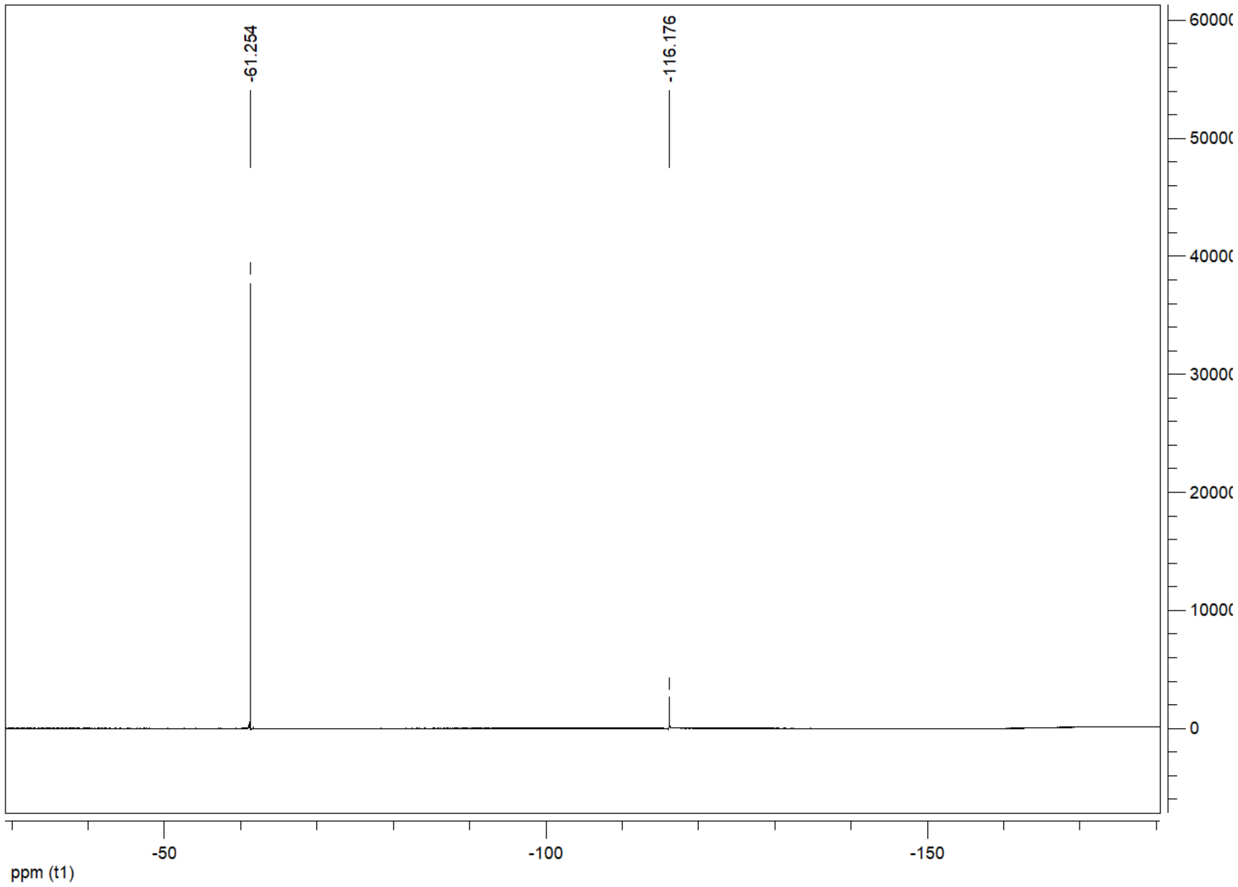
-61.254
-116.176
-50
-100
-150
ppm (t1)
0
10000
20000
30000
40000
50000
60000

^{1}H and ^{13}C NMR spectra of compound **10** (DMSO-d6),

N1,N3-bis(3-(4-methyl-1H-imidazol-1-yl)-5-(trifluoromethyl)phenyl)isophthalamide

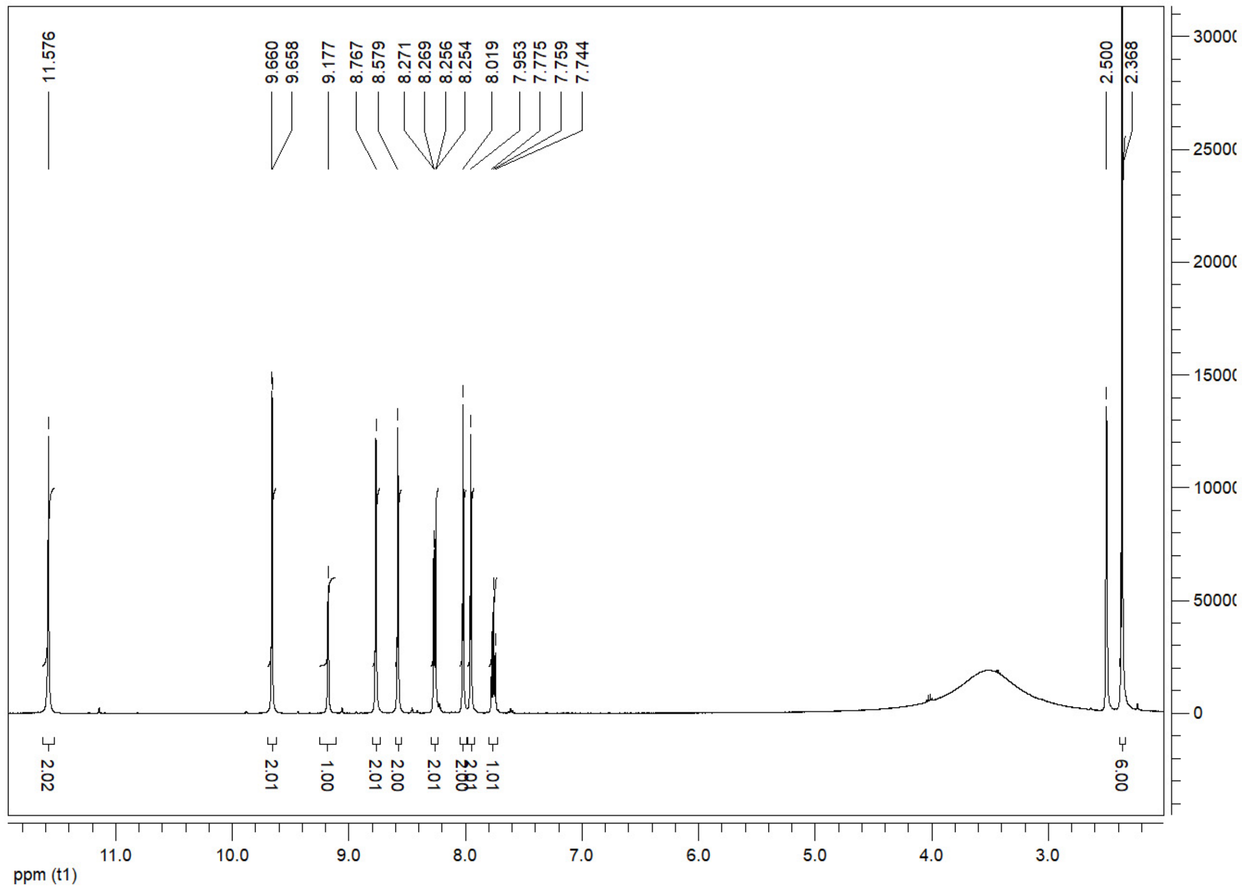

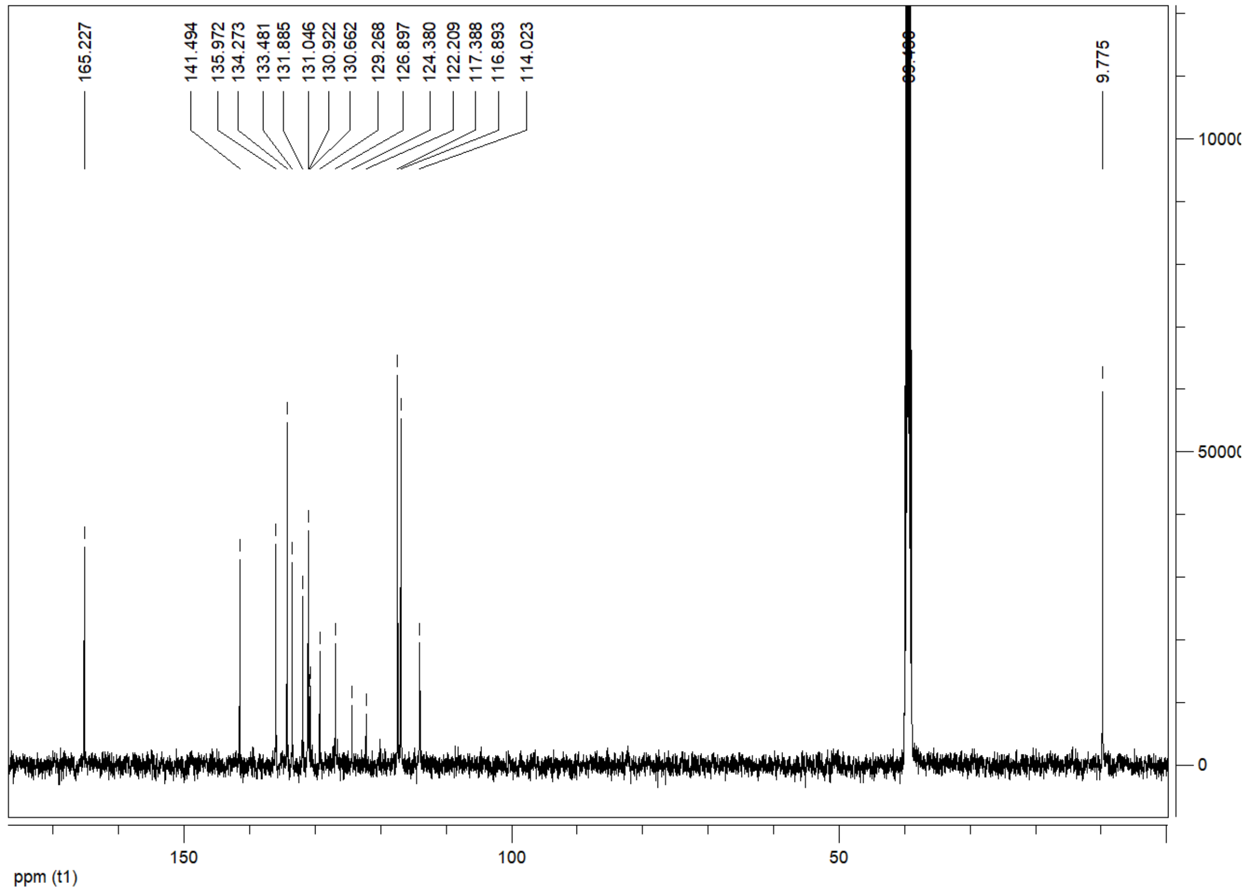

^{1}H, ^{13}C and ^{19}F NMR spectra of compound **11** (DMSO-d6),

N1-(3-(4-methyl-1H-imidazol-1-yl)-5-(trifluoromethyl)phenyl)-N3-(4-((2-(methylcarbamoyl)pyridin-4-yl)oxy)phenyl)isophthalamide

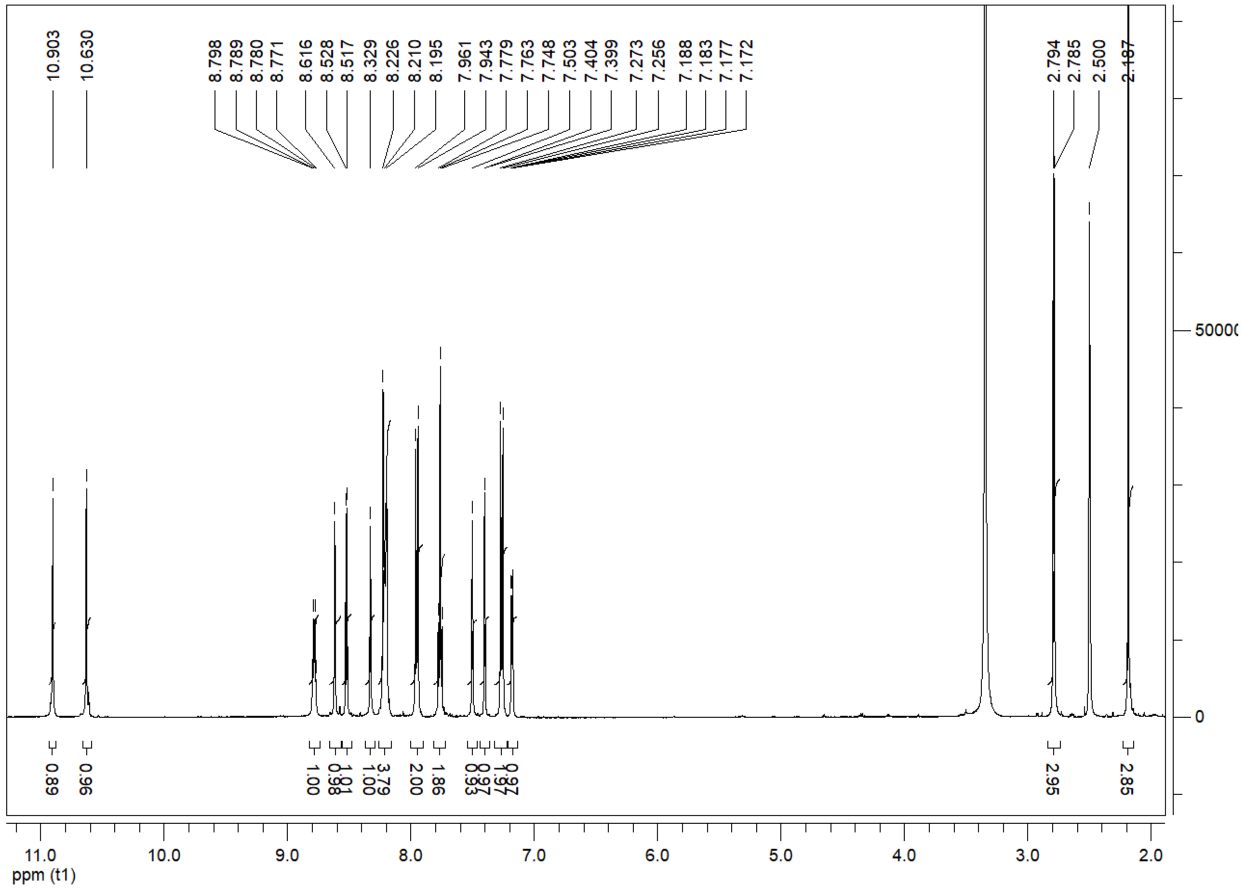

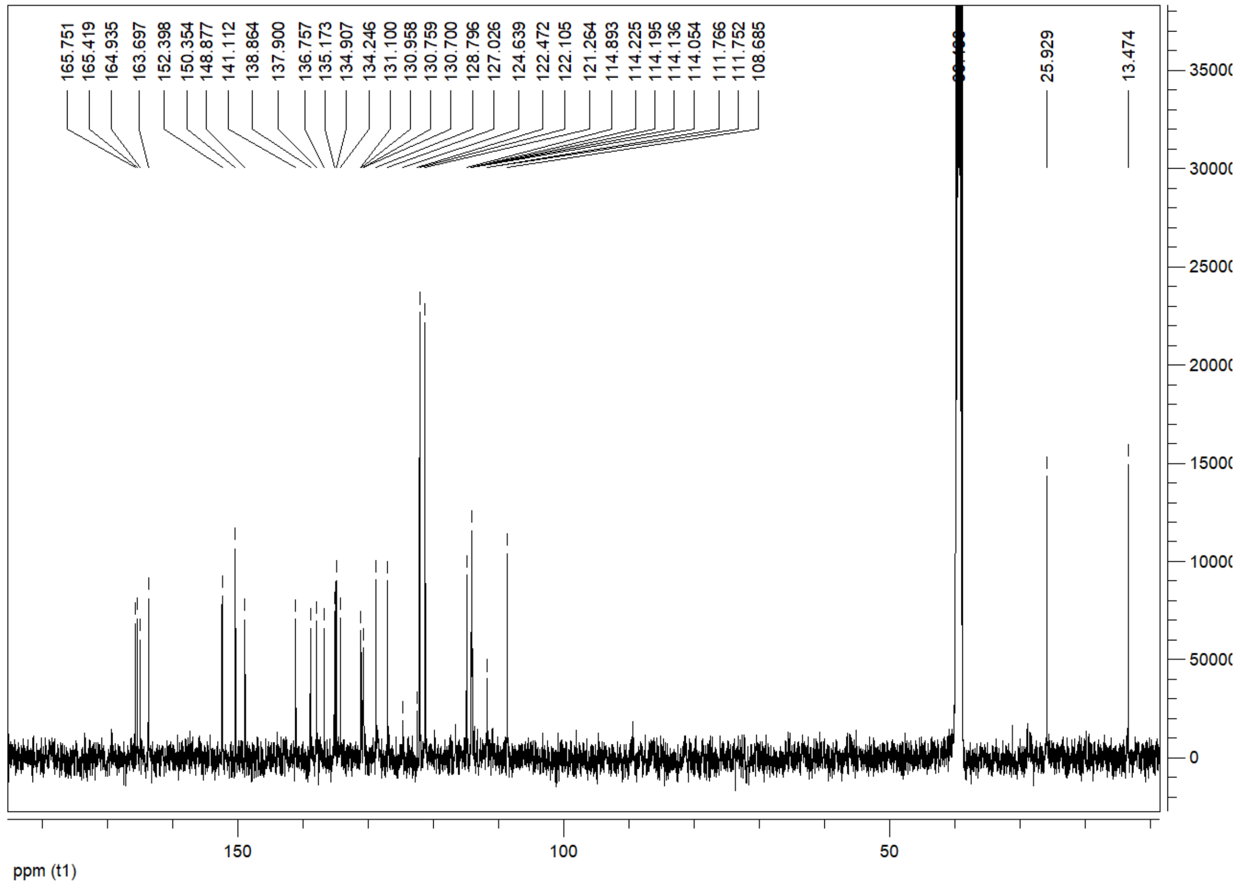

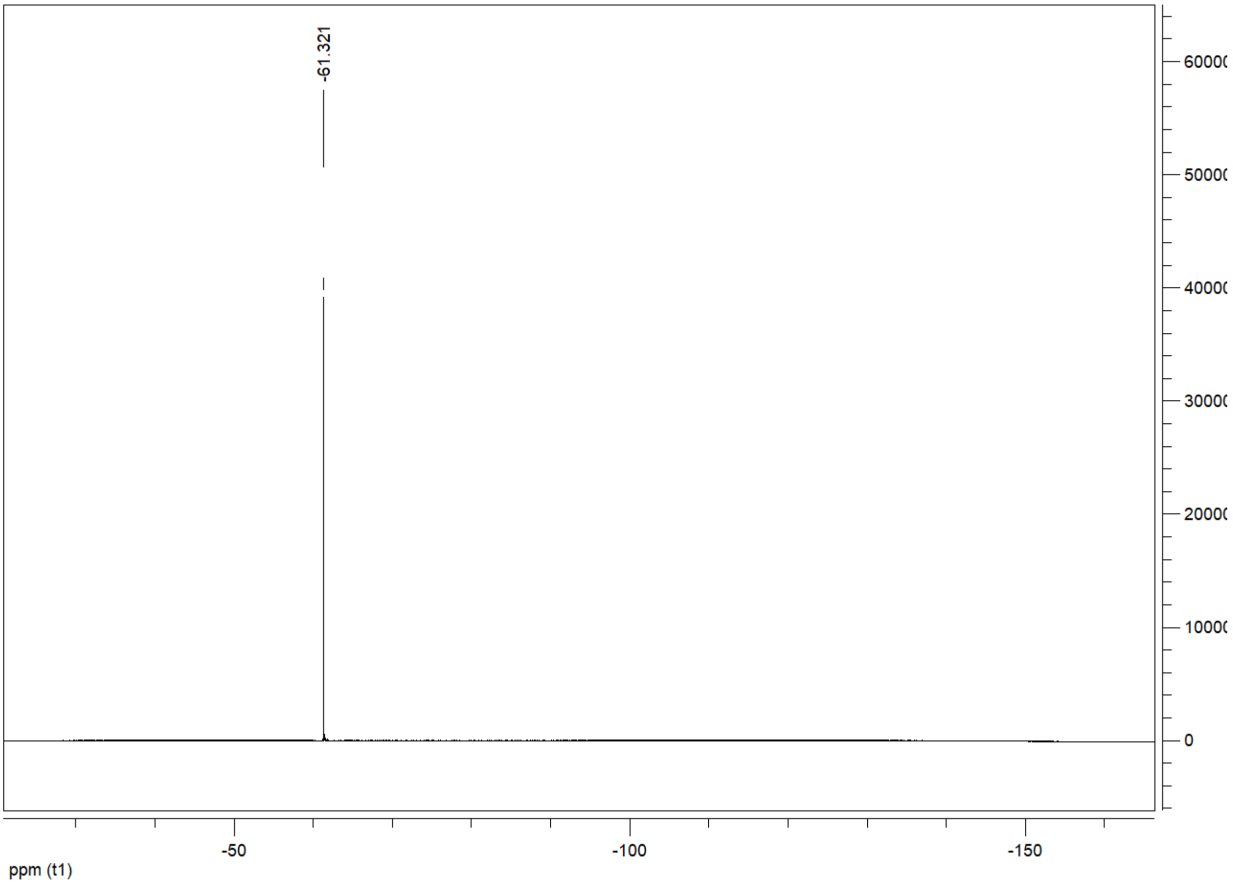

^{1}H, ^{13}C and ^{19}F NMR spectra of compound **12** (DMSO-d6),

N1-(3-(4-methyl-1H-imidazol-1-yl)-5-(trifluoromethyl)phenyl)-N3-(2-methyl-5-nitrophenyl)-isophthalamide

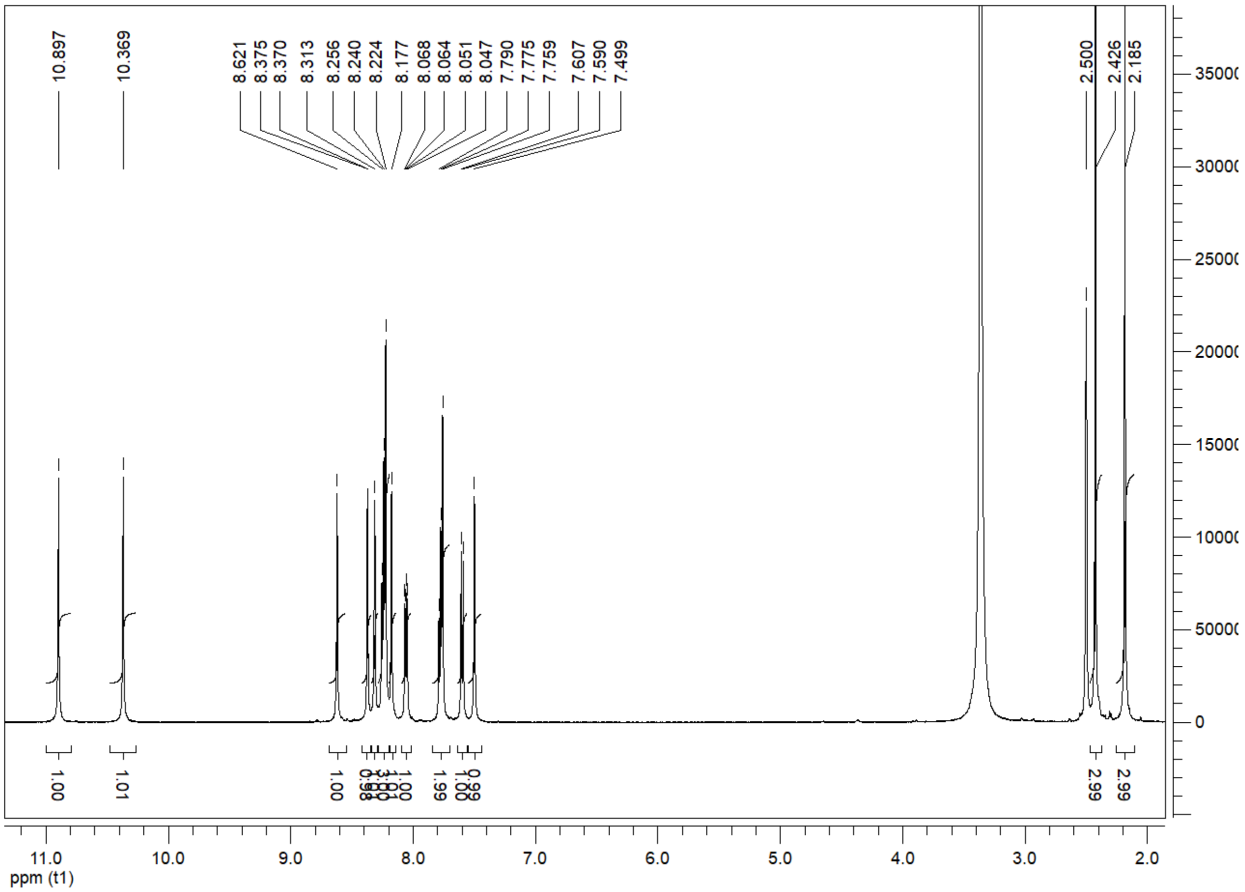

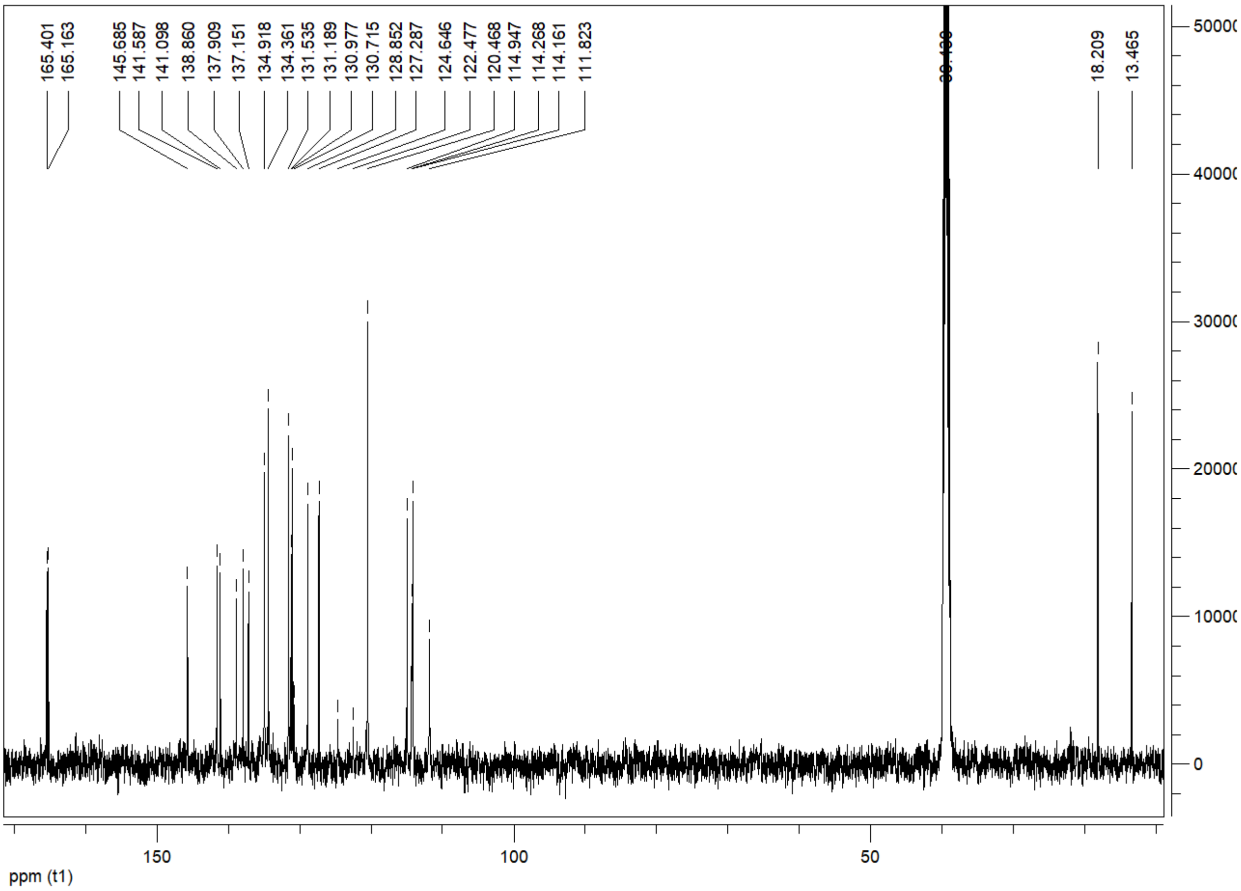
165.401
165.163
145.685
141.587
141.098
138.860
137.909
137.151
134.918
134.361
131.535
131.189
130.977
130.715
128.852
127.287
124.646
122.477
120.468
114.947
114.268
114.161
111.823
18.209
13.465
150
100
50
ppm (t1)
0
10000
20000
30000
40000
50000

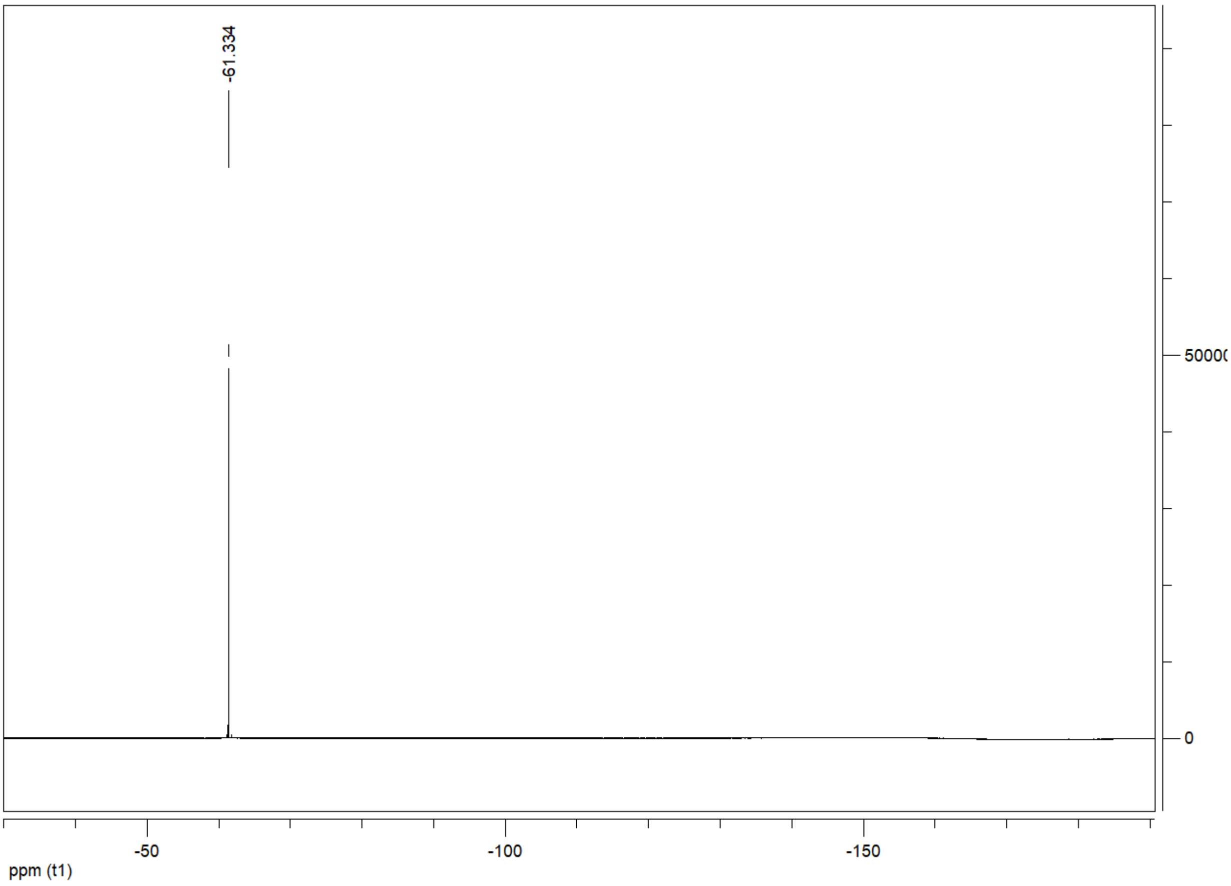
-61.334
-50
-100
-150
ppm (t1)
0
50000

^{1}H and ^{13}C NMR spectra of compound **13** (DMSO-d6),

3-(4-(4-Methoxybenzoyl)piperazine-1-carbonyl)-N-(3-(4-methyl-1H-imidazol-1-yl)-5-(trifluoromethyl)phenyl)benzamide

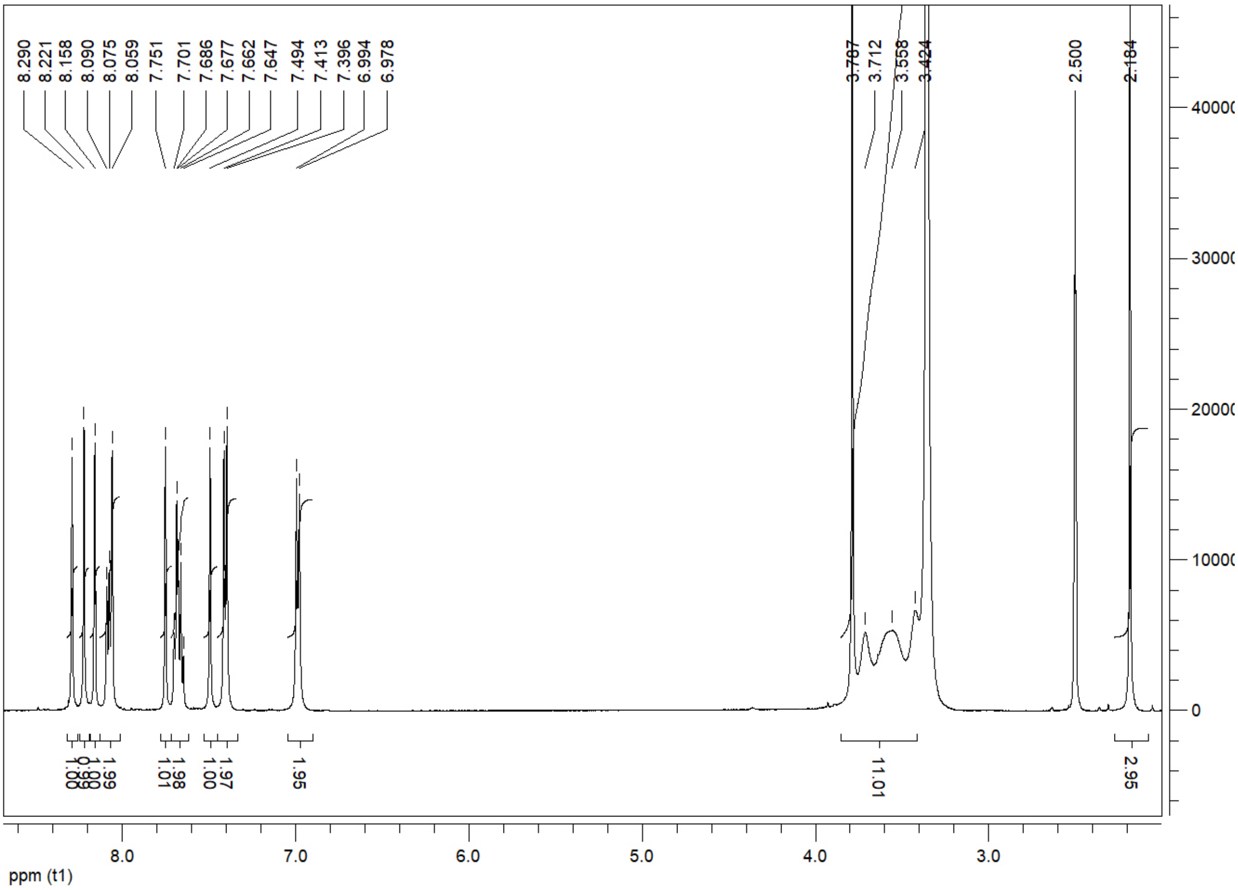

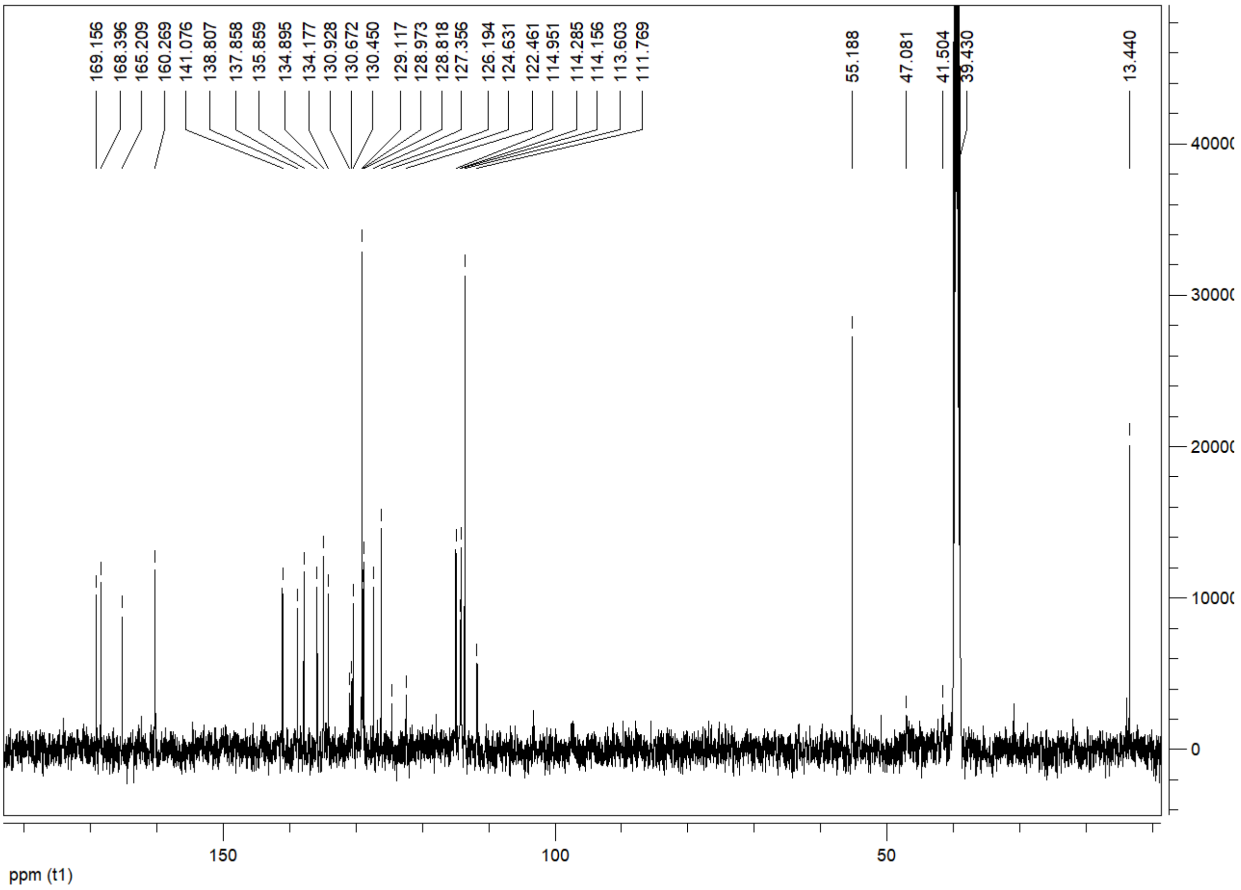

^{1}H, ^{13}C and ^{19}F NMR spectra of compound **14** (DMSO-d6),

3-(4-(2-Fluorobenzoyl)piperazine-1-carbonyl)-N-(3-(4-methyl-1H-imidazol-1-yl)-5-(trifluoromethyl)phenyl)benzamide

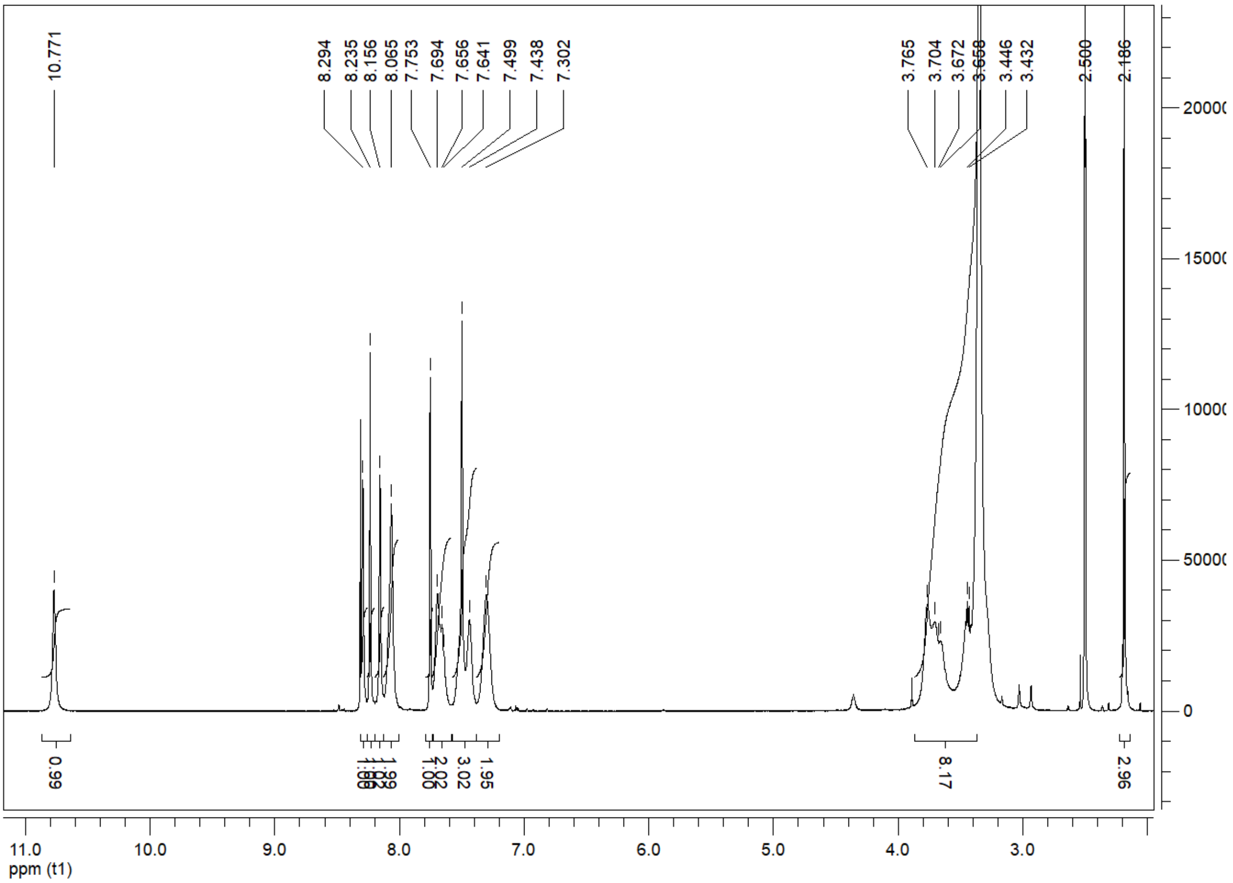

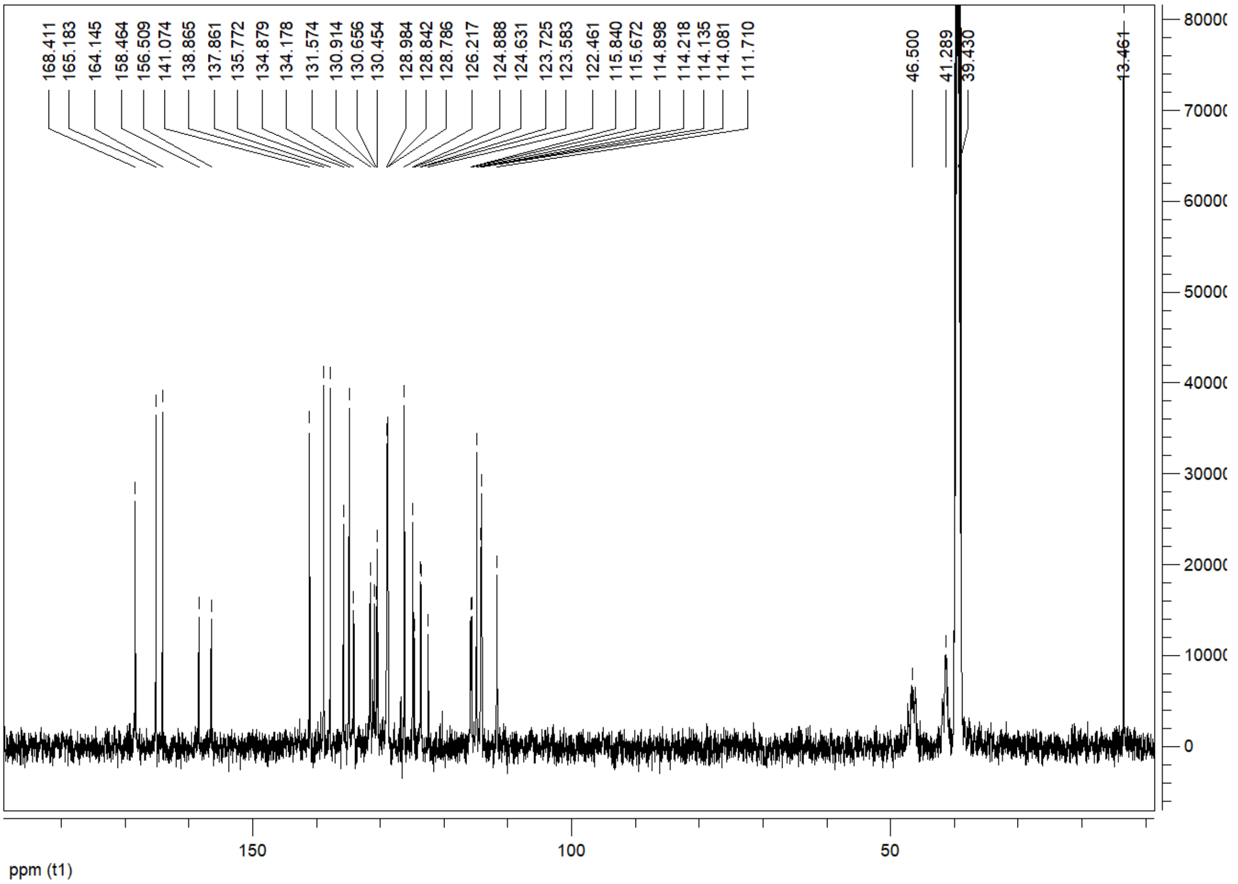

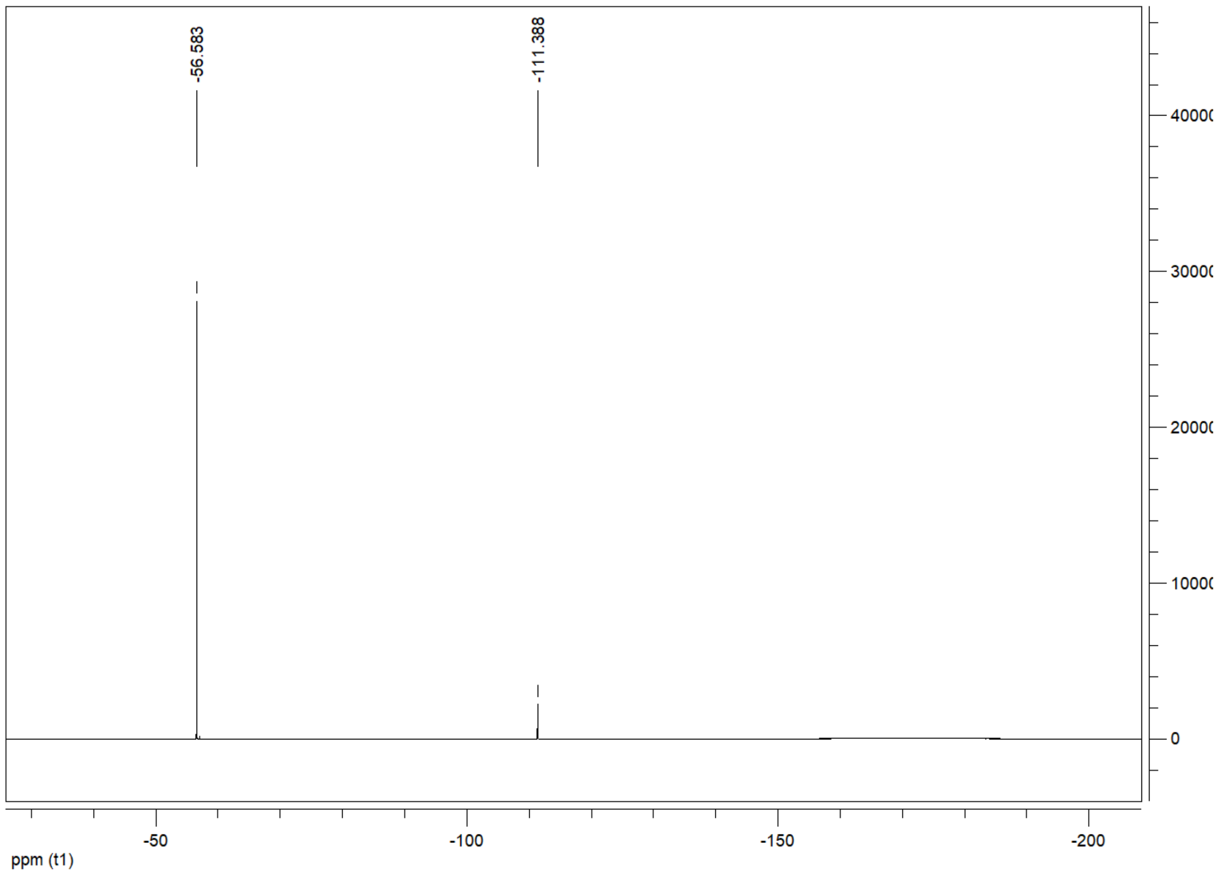

^{1}H, ^{13}C and ^{19}F NMR spectra of compound **15** (DMSO-d6),

3-(4-(3-Fluorobenzoyl)piperazine-1-carbonyl)-N-(3-(4-methyl-1H-imidazol-1-yl)-5-(trifluoromethyl)phenyl)benzamide

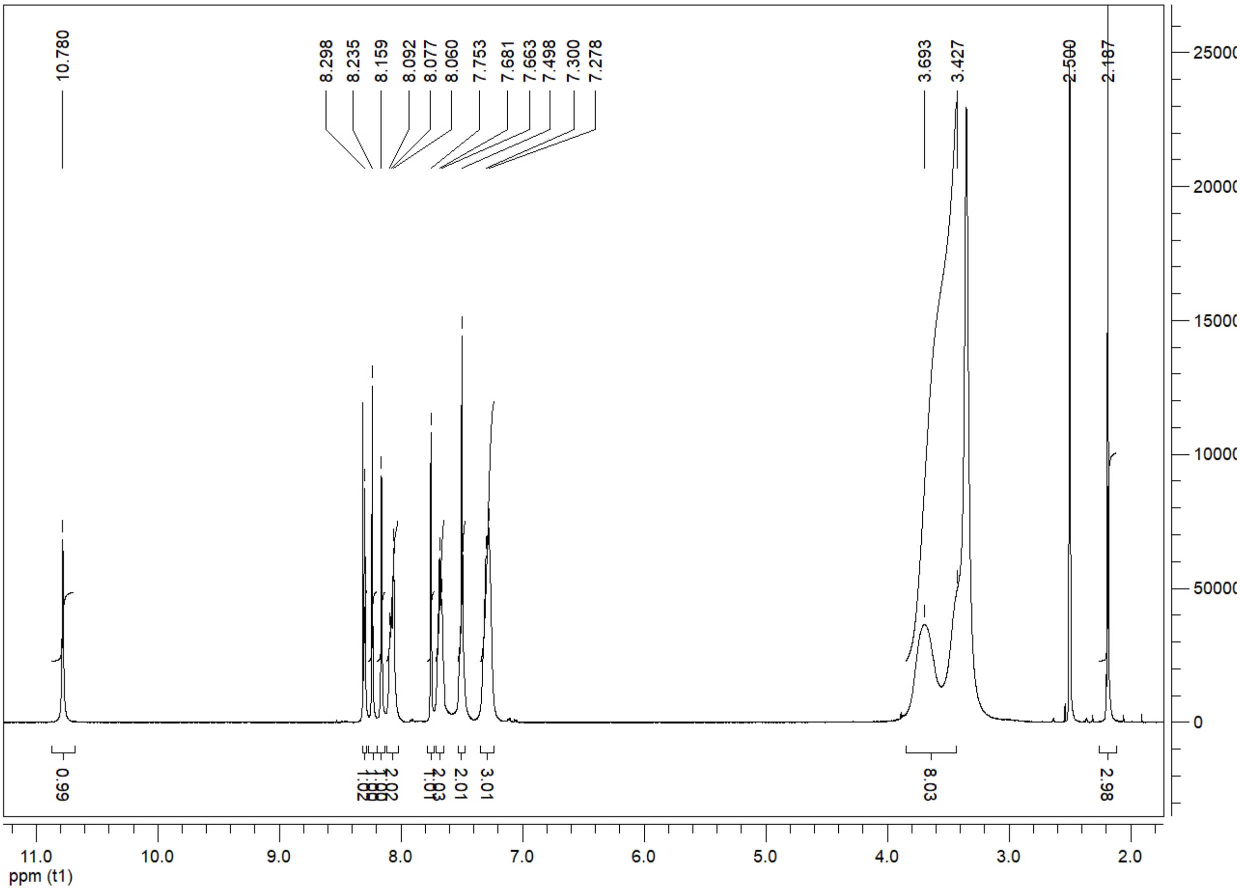

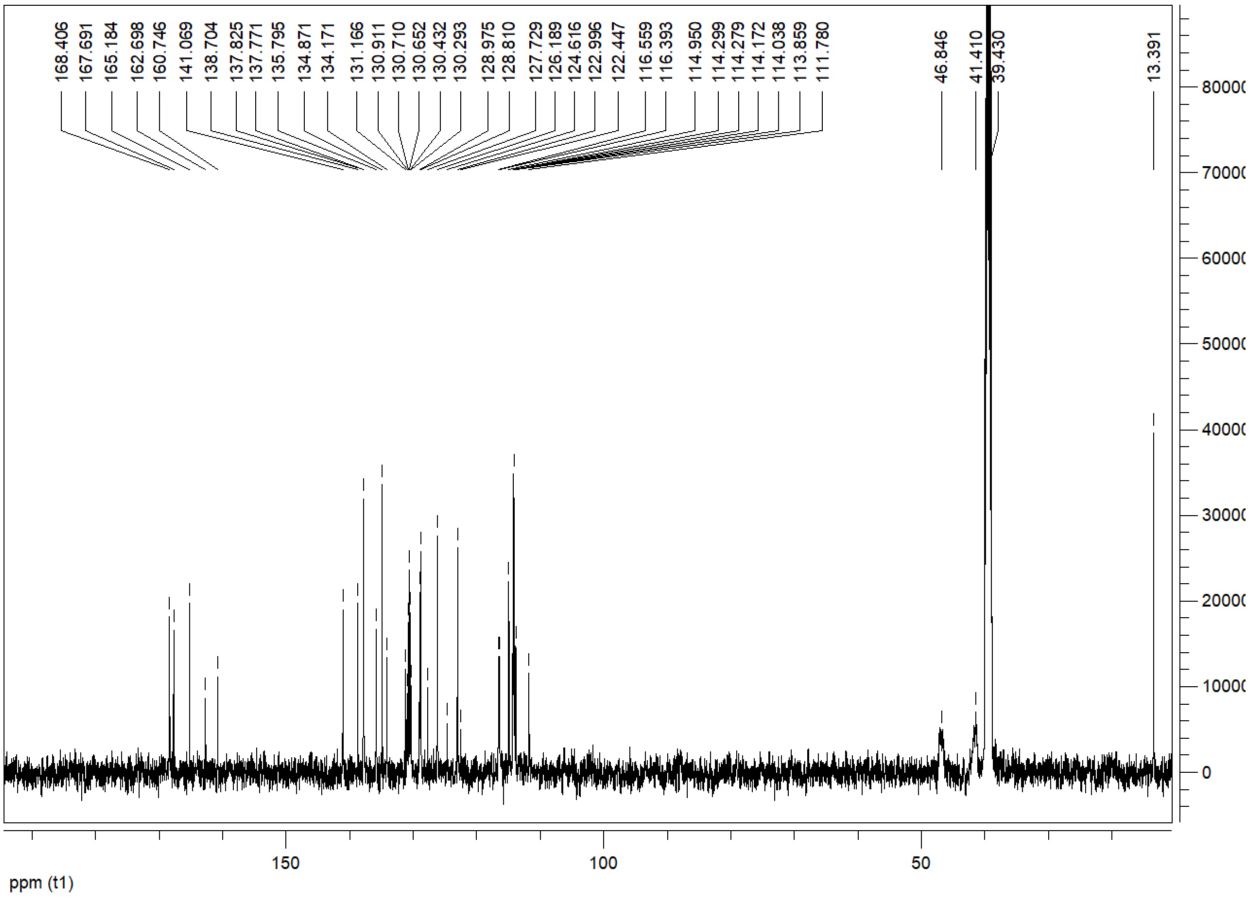
168.406
167.691
165.184
162.698
160.746
141.069
138.704
137.825
137.771
135.795
134.871
134.171
131.166
130.911
130.710
130.652
130.432
130.293
128.975
128.810
127.729
126.189
124.616
122.996
122.447
116.559
116.393
114.950
114.299
114.279
114.172
114.038
113.859
111.780
46.846
41.410
39.430
13.391
150
100
50
ppm (t1)

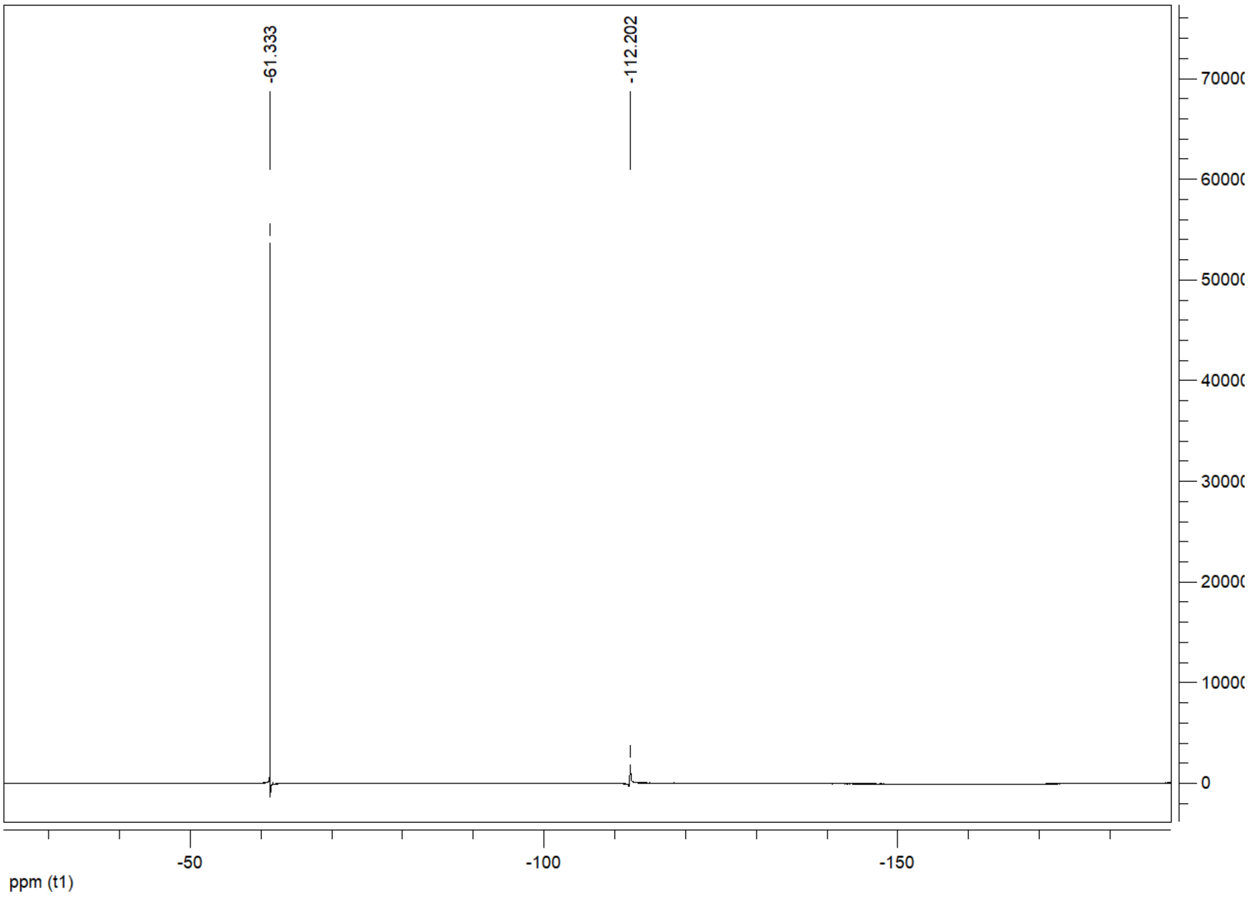
-61.333
-112.202
-50
-100
-150
ppm (t1)

^{1}H and ^{13}C NMR spectra of compound **16** (DMSO-d6),

N1-(2-methyl-5-nitrophenyl)-N3-(4-((2-(methylcarbamoyl)pyridin-4-yl)oxy)phenyl)isophthalamide

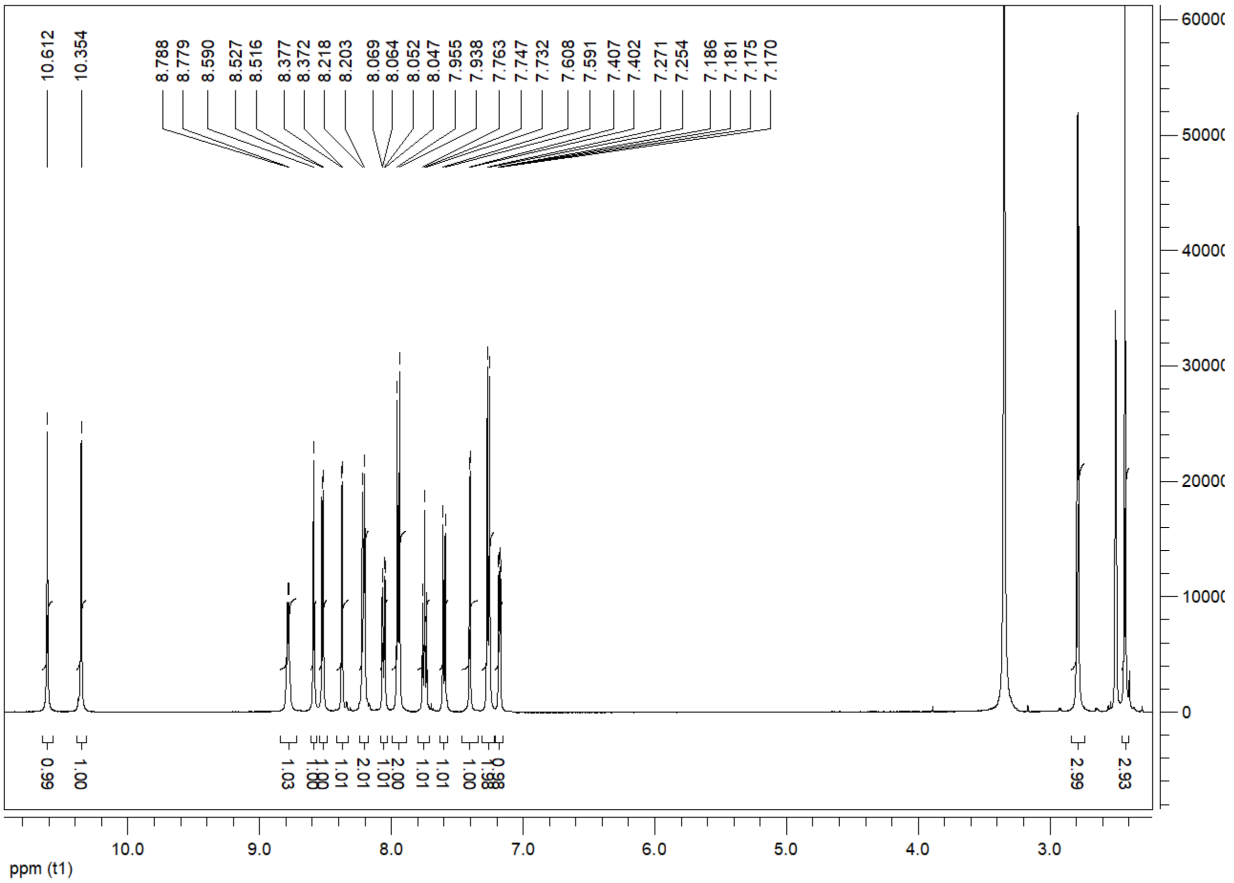

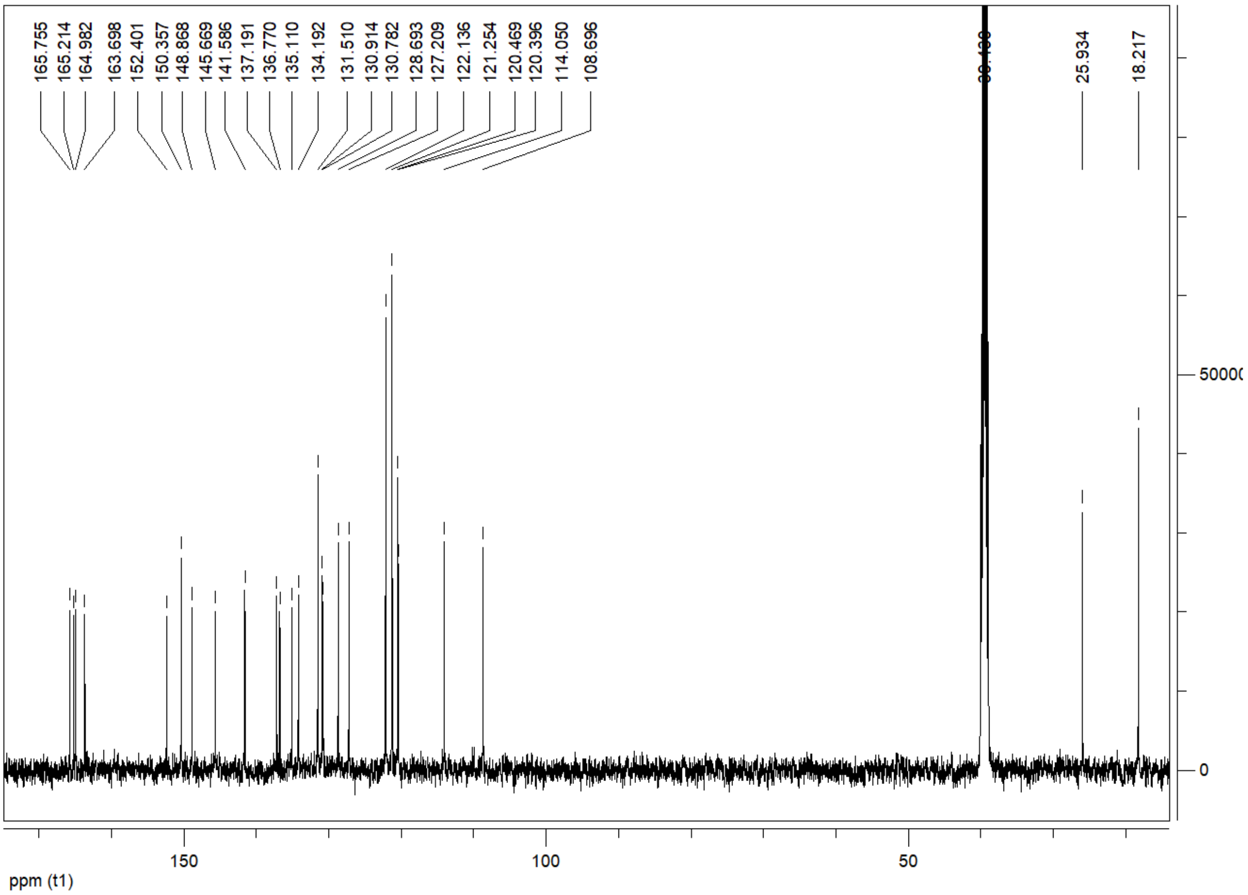

^{1}H, ^{13}C and ^{19}F NMR spectra of compound **17** (DMSO-d6),

N1-(3-(4-methyl-1H-imidazol-1-yl)-5-(trifluoromethyl)phenyl)-N4-(3-(trifluoromethyl)phenyl)-terephthalamide

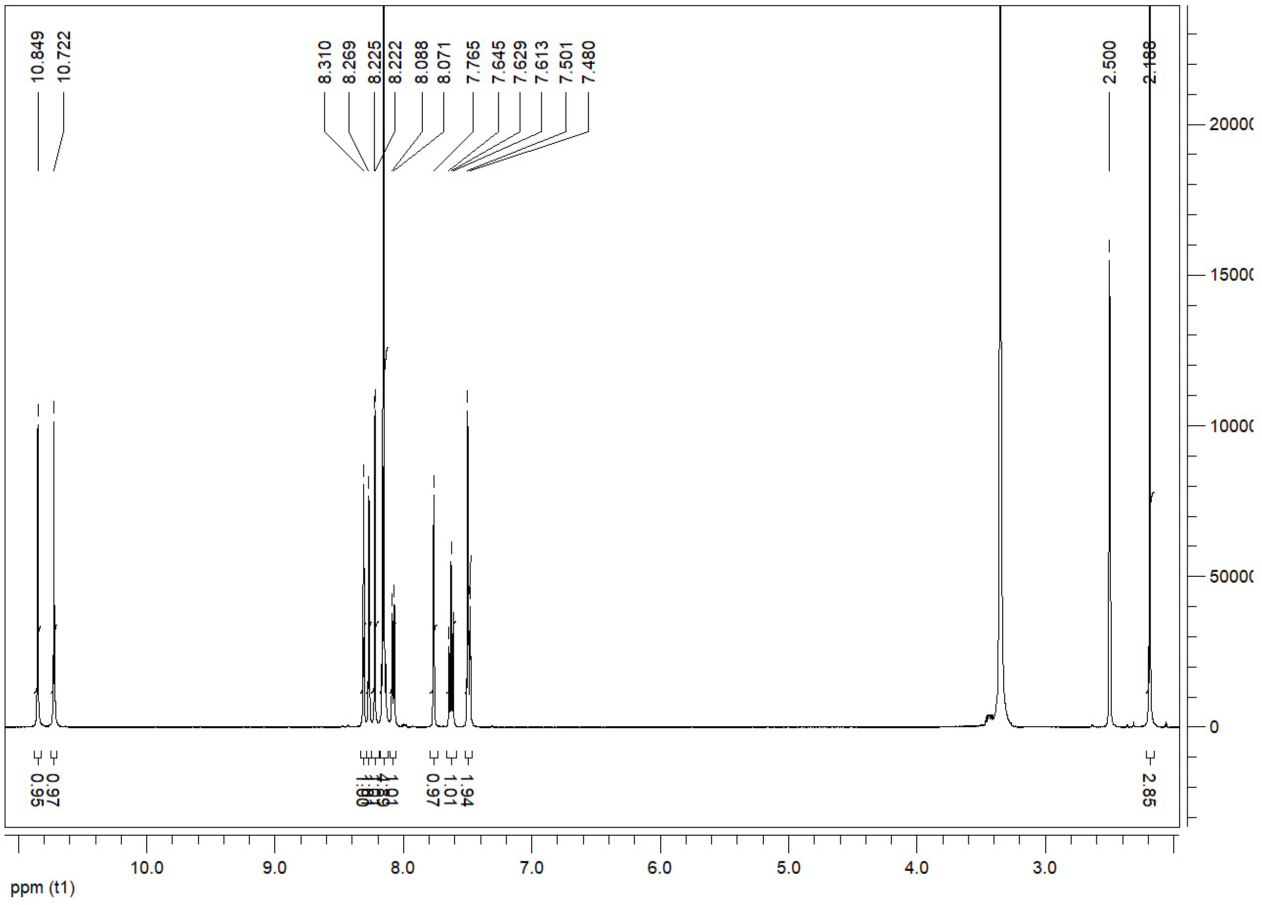

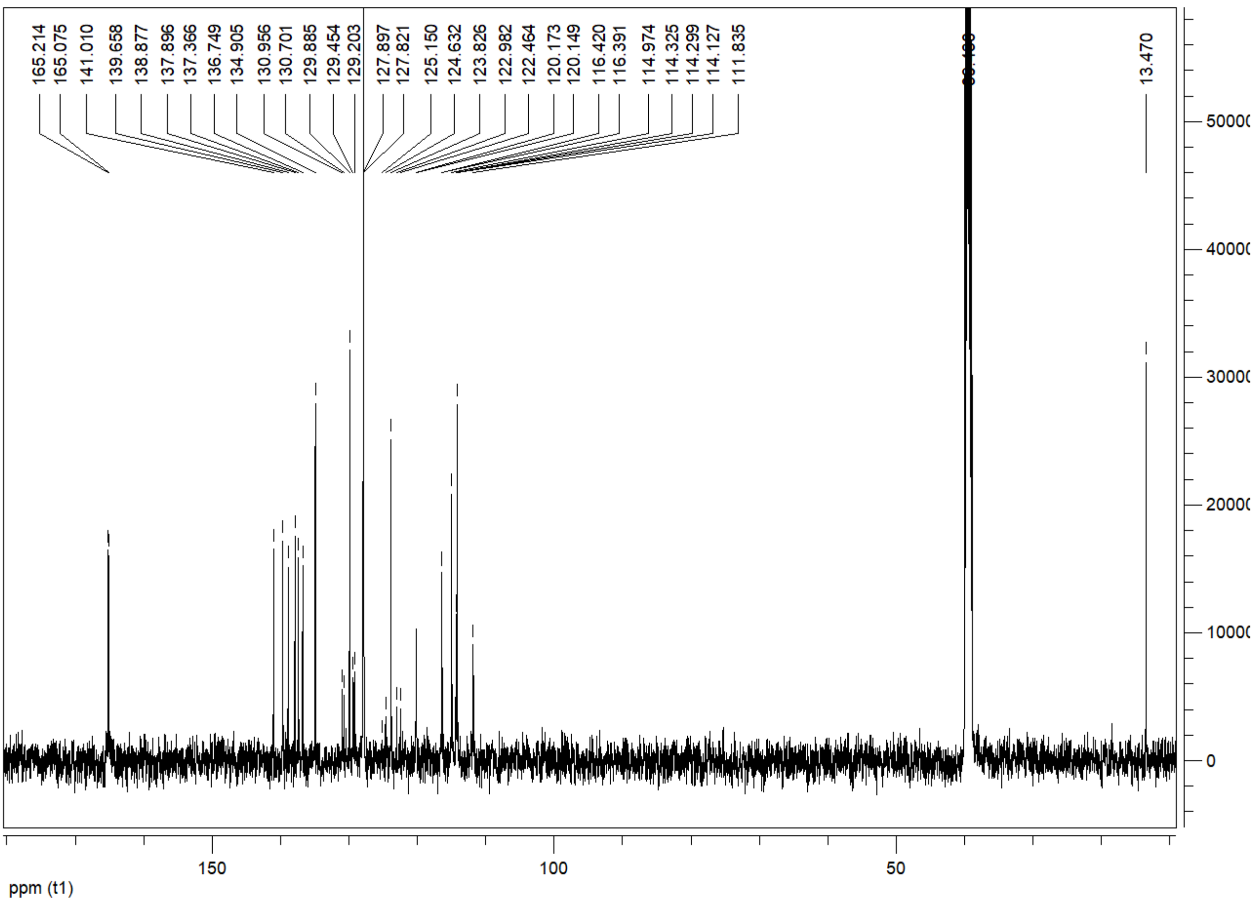

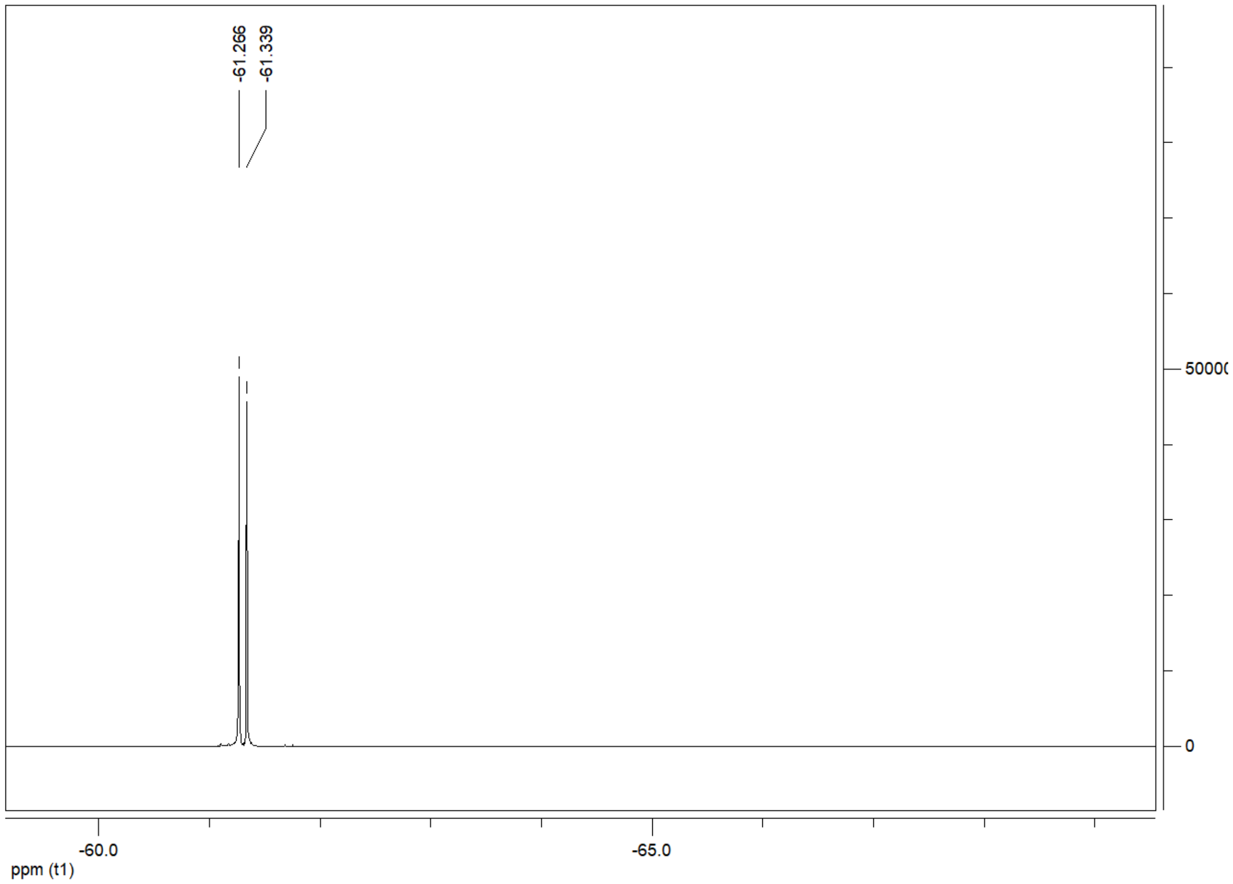

^{1}H, ^{13}C and ^{19}F NMR spectra of compound **18** (DMSO-d6),

N1-(4-((2-(methylcarbamoyl)pyridin-4-yl)oxy)phenyl)-N4-(3-(trifluoromethyl)phenyl)terephthalamide

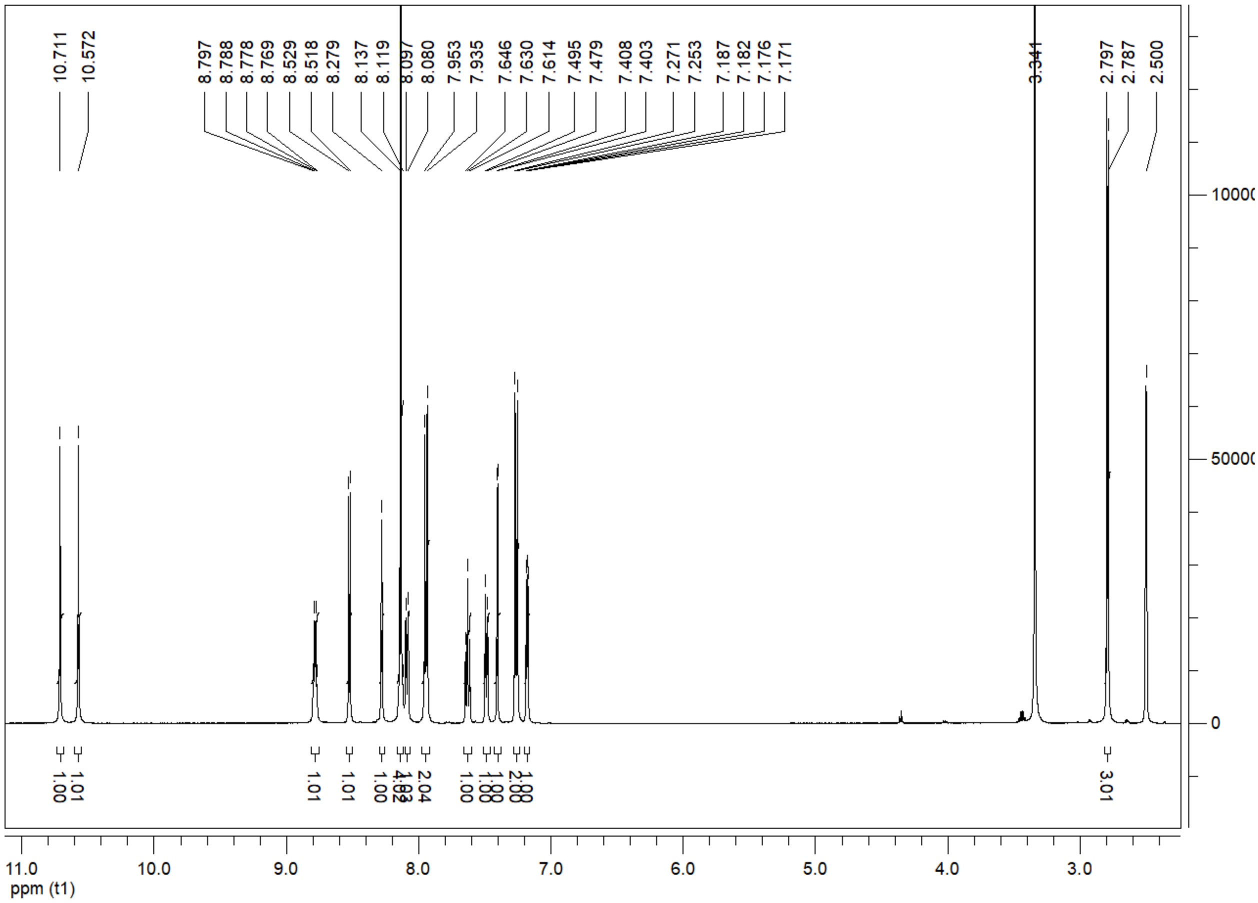

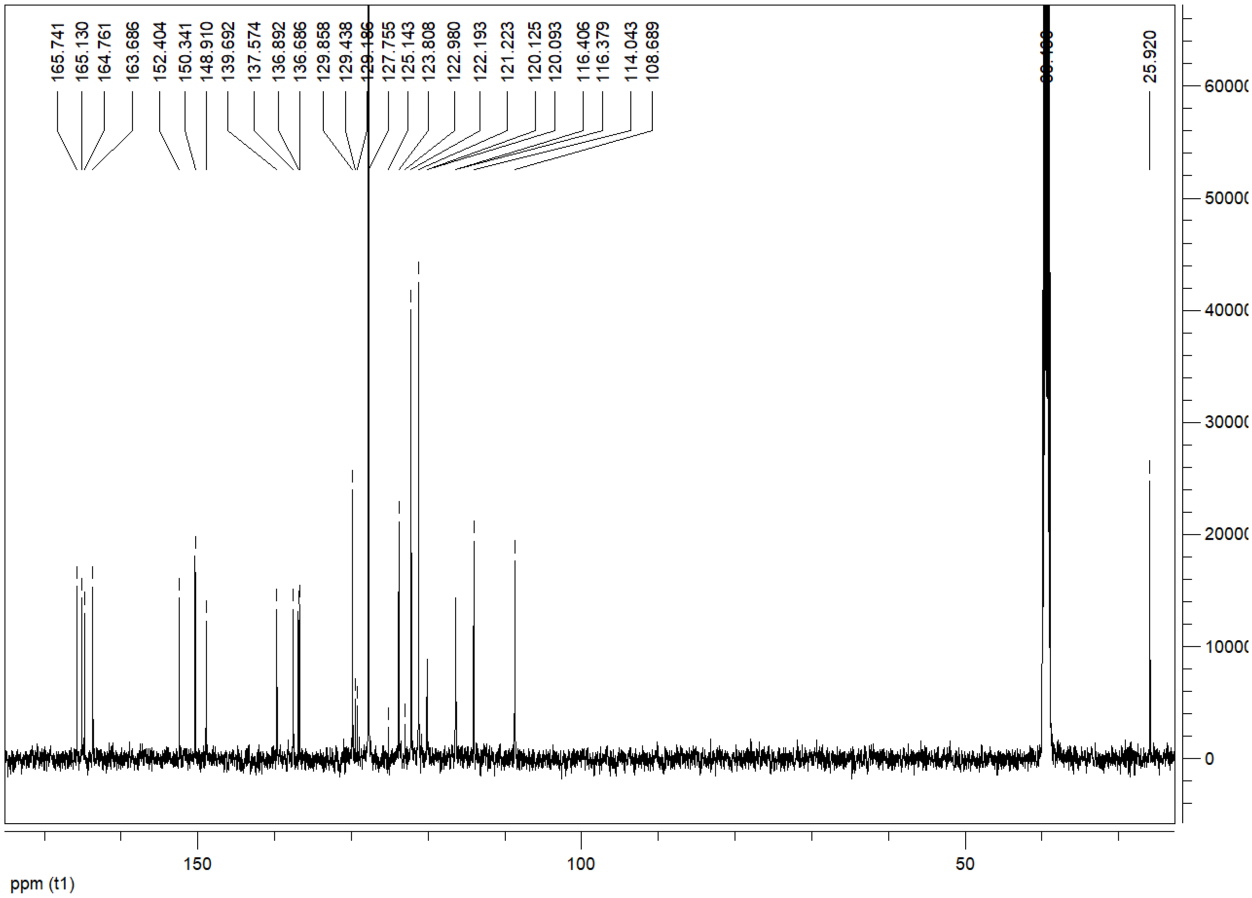

165.741
165.130
164.761
163.686
152.404
150.341
148.910
139.692
137.574
136.892
136.686
129.858
129.438
127.755
125.143
123.808
122.980
122.193
121.223
120.125
120.093
116.406
116.379
114.043
108.689
25.920
60000
50000
40000
30000
20000
10000
0
150
100
50
ppm (t1)

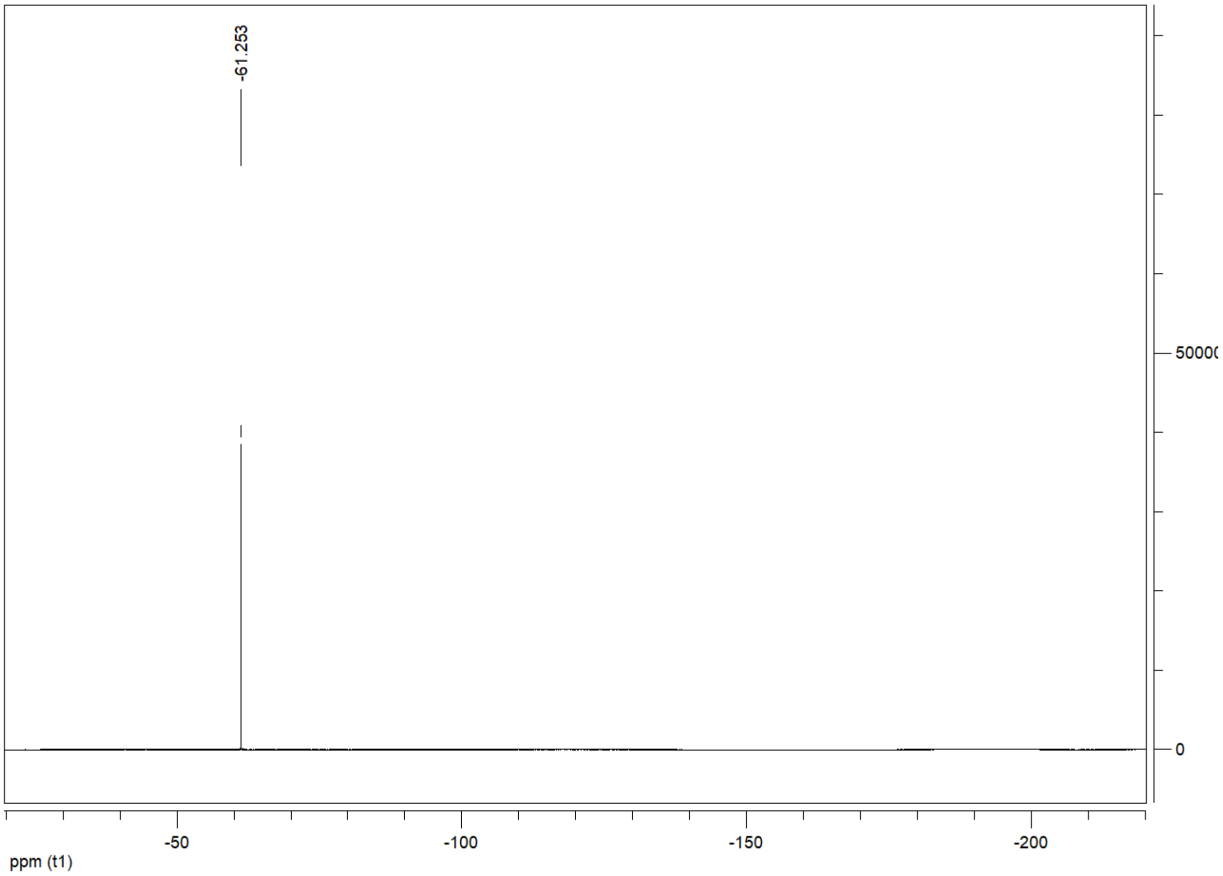

-61.253
50000
0
-50
-100
-150
-200
ppm (t1)

^{1}H, ^{13}C and ^{19}F NMR spectra of compound **19** (DMSO-d6),

4-(4-(4-Methoxybenzoyl)piperazine-1-carbonyl)-N-(3-(trifluoromethyl)phenyl)benzamide

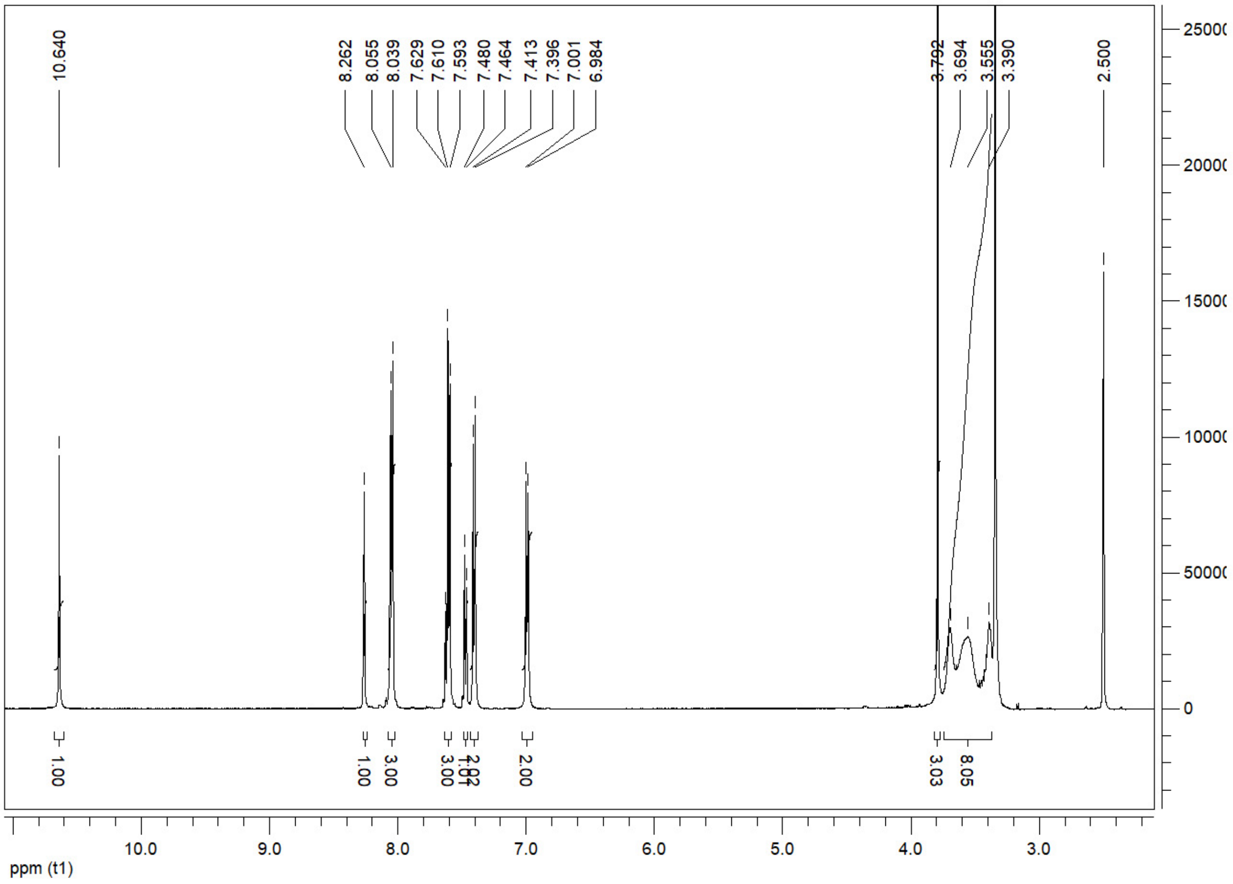

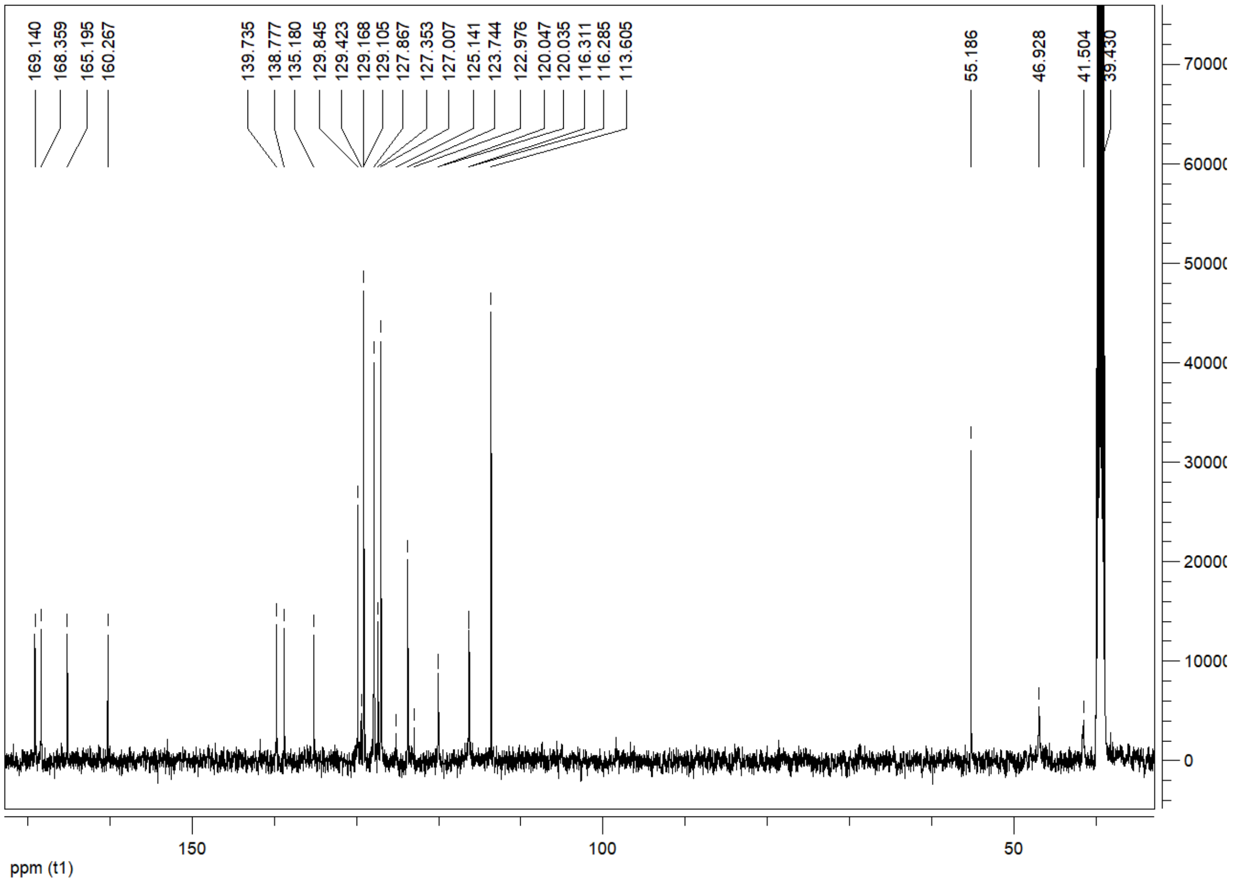

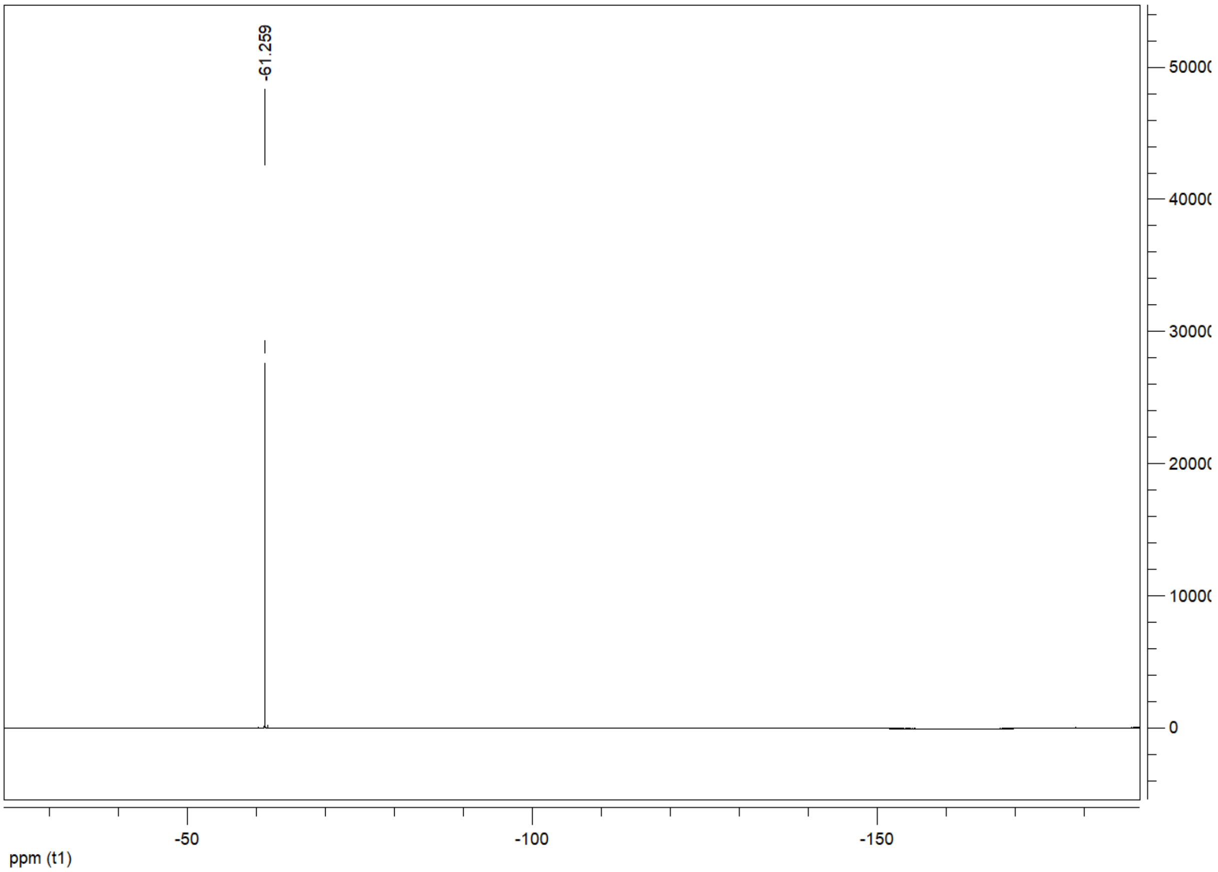

^{1}H, ^{13}C and ^{19}F NMR spectra of compound **20** (DMSO-d6),

4-(4-(2-Fluorobenzoyl)piperazine-1-carbonyl)-N-(3-(trifluoromethyl)phenyl)benzamide

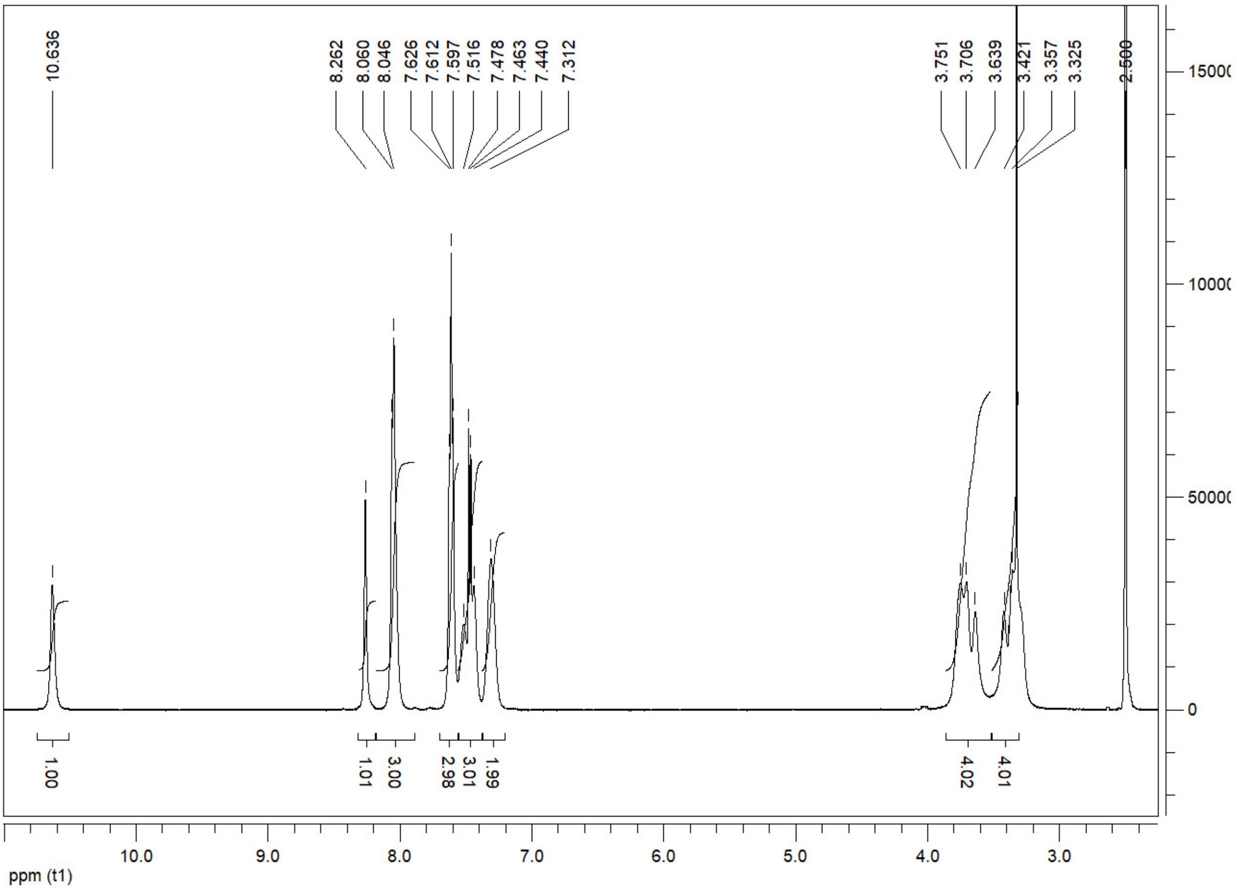

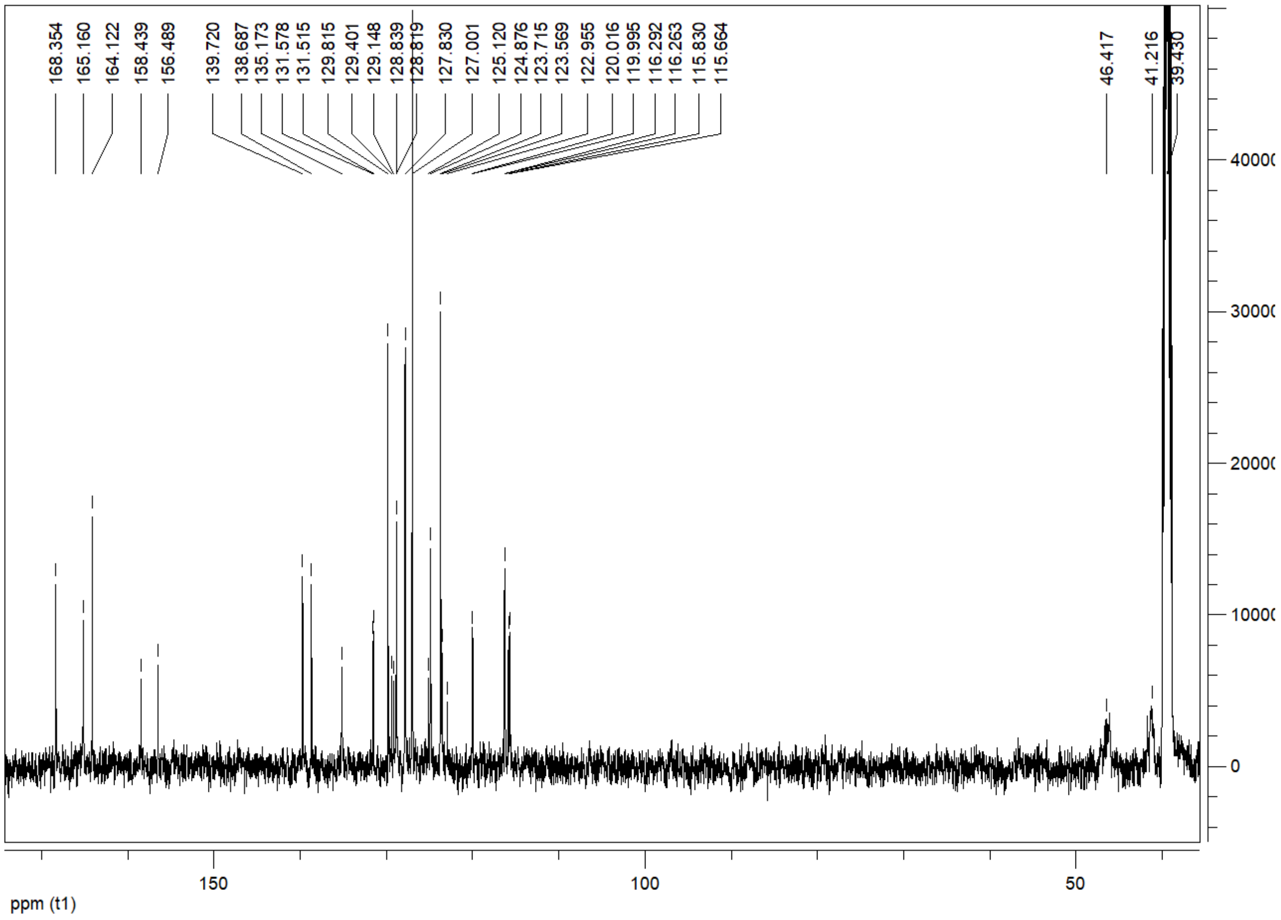
168.354
165.160
164.122
158.439
156.489
139.720
138.687
135.173
131.578
131.515
129.815
129.401
129.148
128.839
128.819
127.830
127.001
125.120
124.876
123.715
123.569
122.955
120.016
119.995
116.292
116.263
115.830
115.664
46.417
41.216
39.430
150
100
50
ppm (t1)

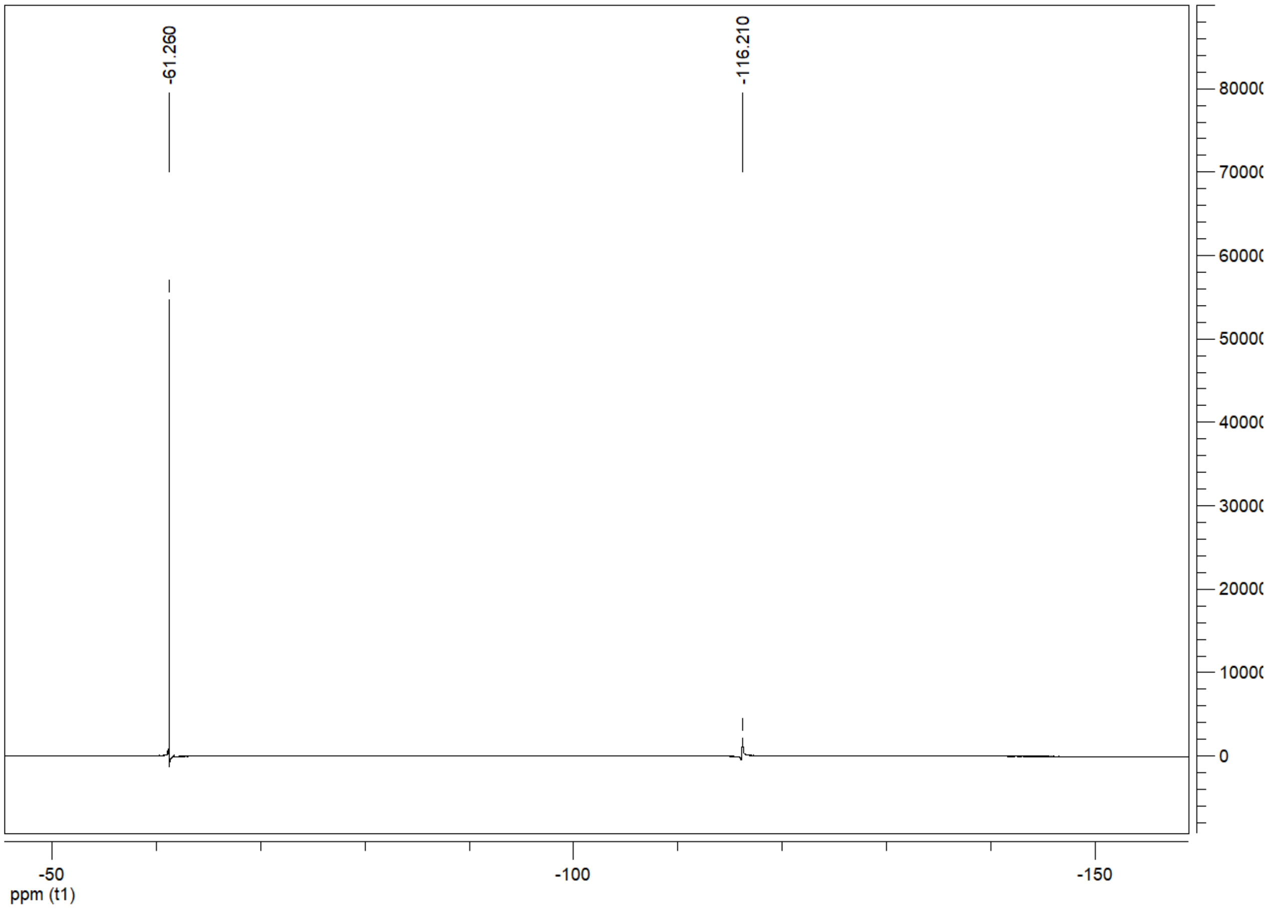
-61.260
-116.210
-50
-100
-150
ppm (t1)

^{1}H, ^{13}C and ^{19}F NMR spectra of compound **21** (DMSO-d6),
4-(4-(3-Fluorobenzoyl)piperazine-1-carbonyl)-N-(3-(trifluoromethyl)phenyl)benzamide

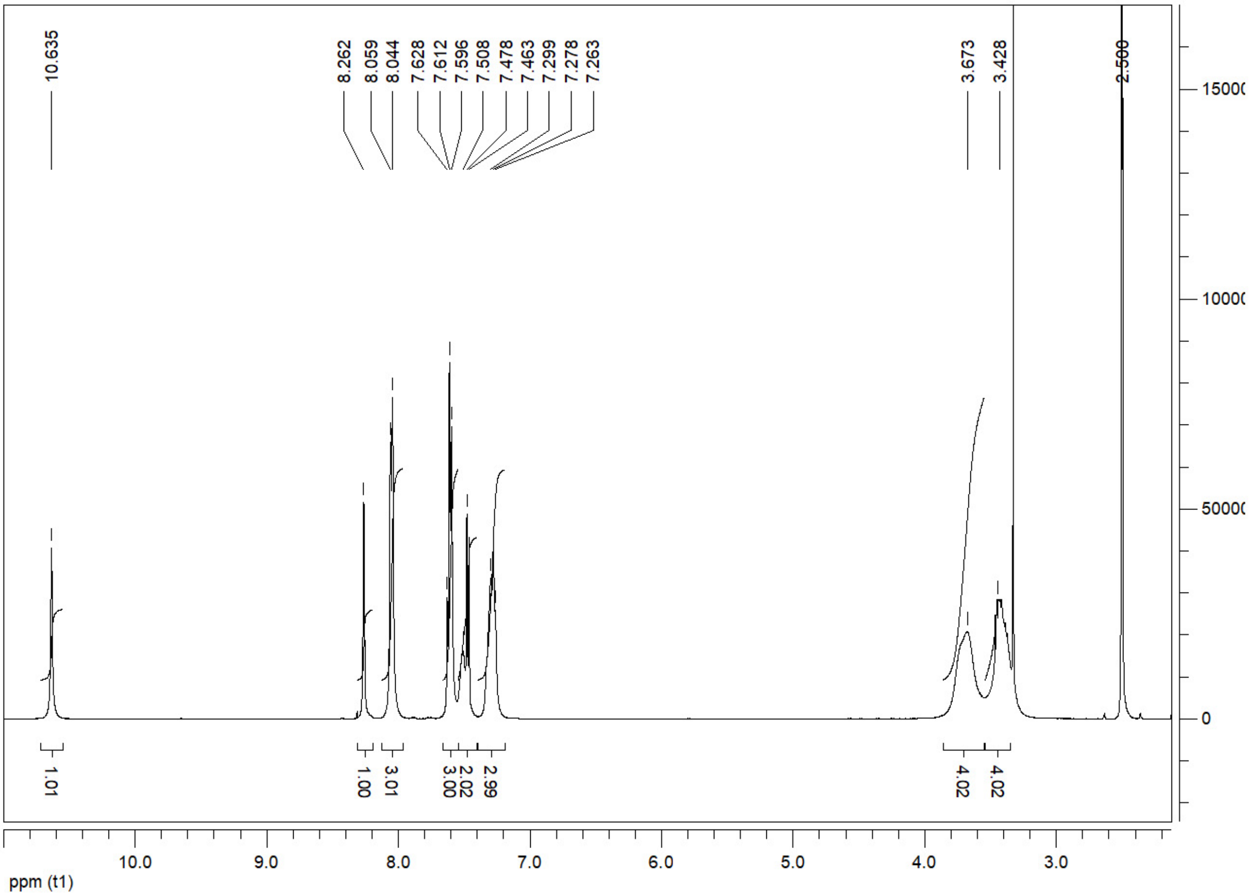

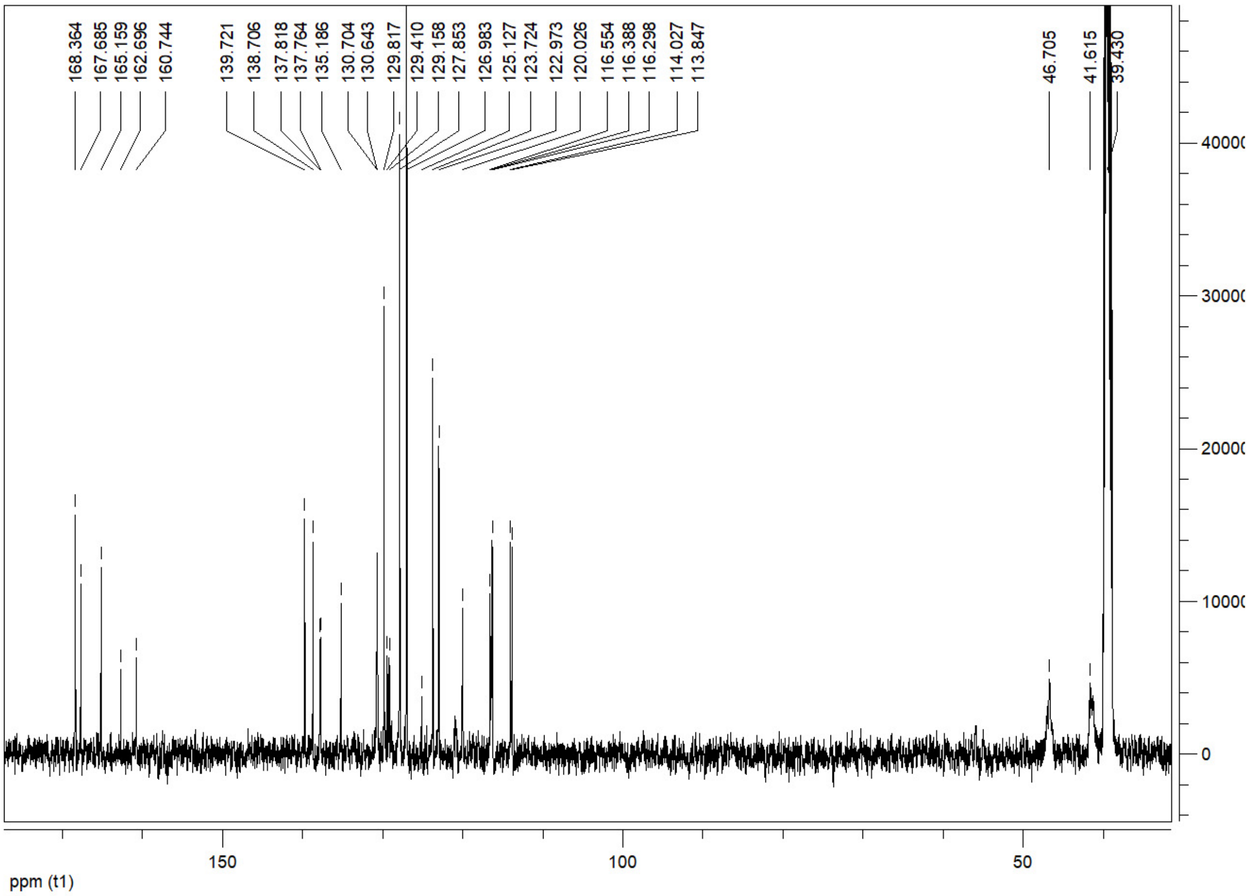

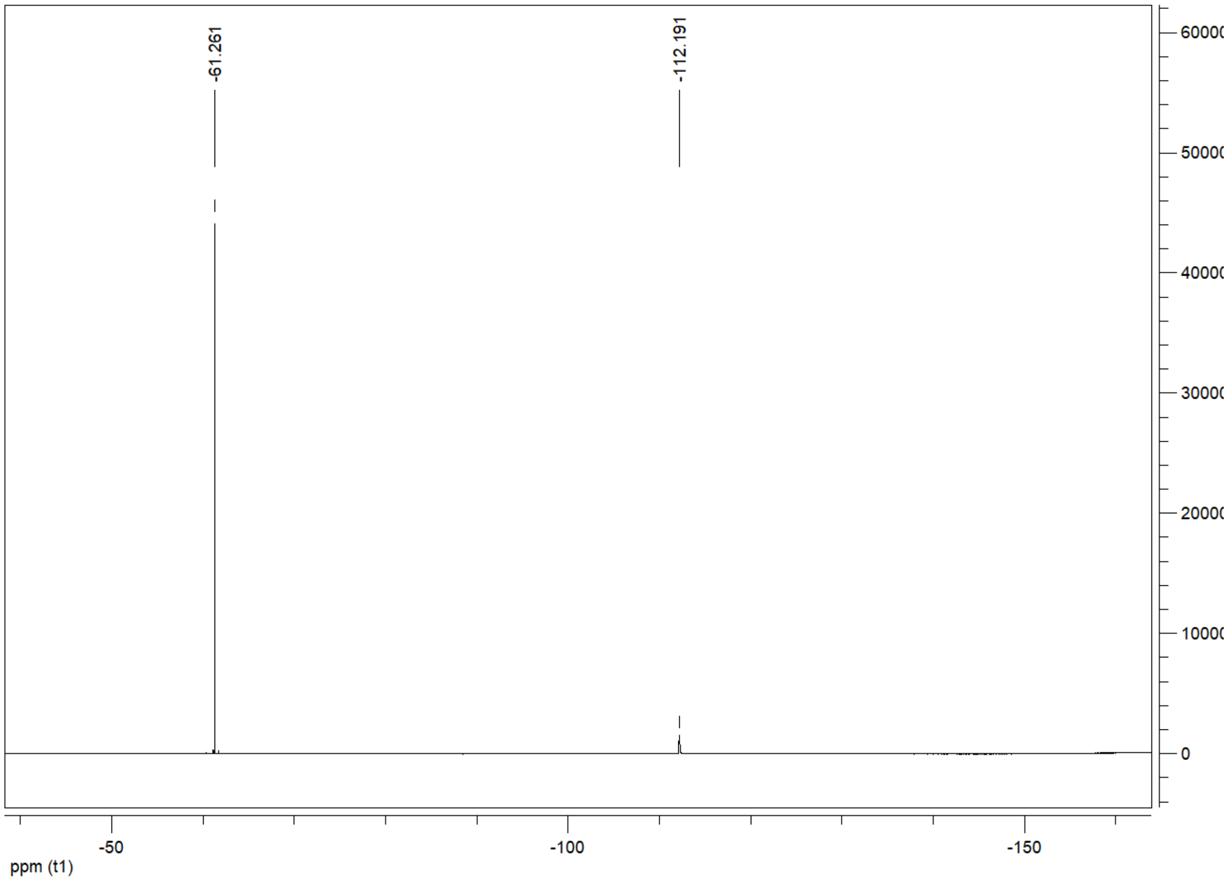

^{1}H and ^{13}C NMR spectra of compound **22** (DMSO-d6),

N1,N4-bis(3-(4-methyl-1H-imidazol-1-yl)-5-(trifluoromethyl)phenyl)terephthalamide

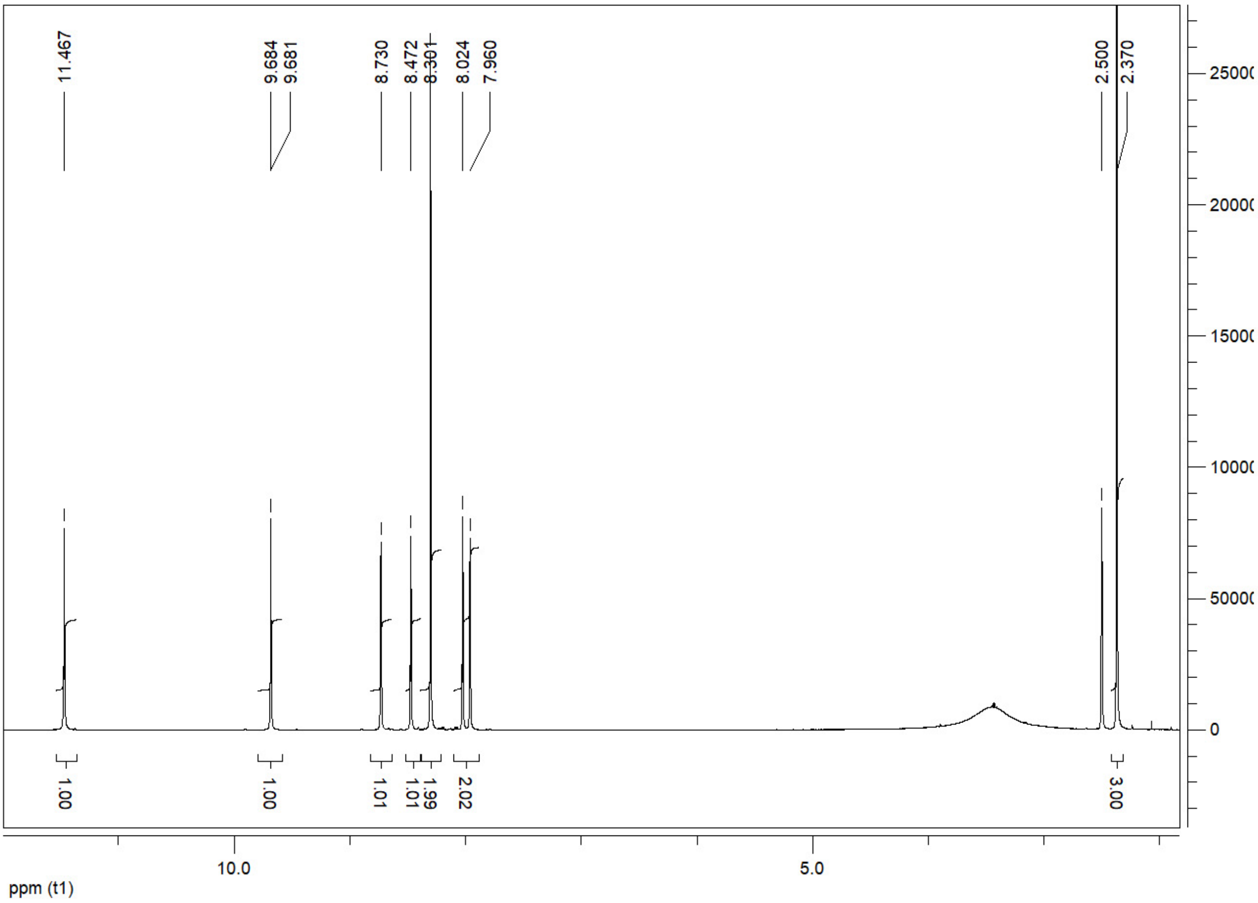

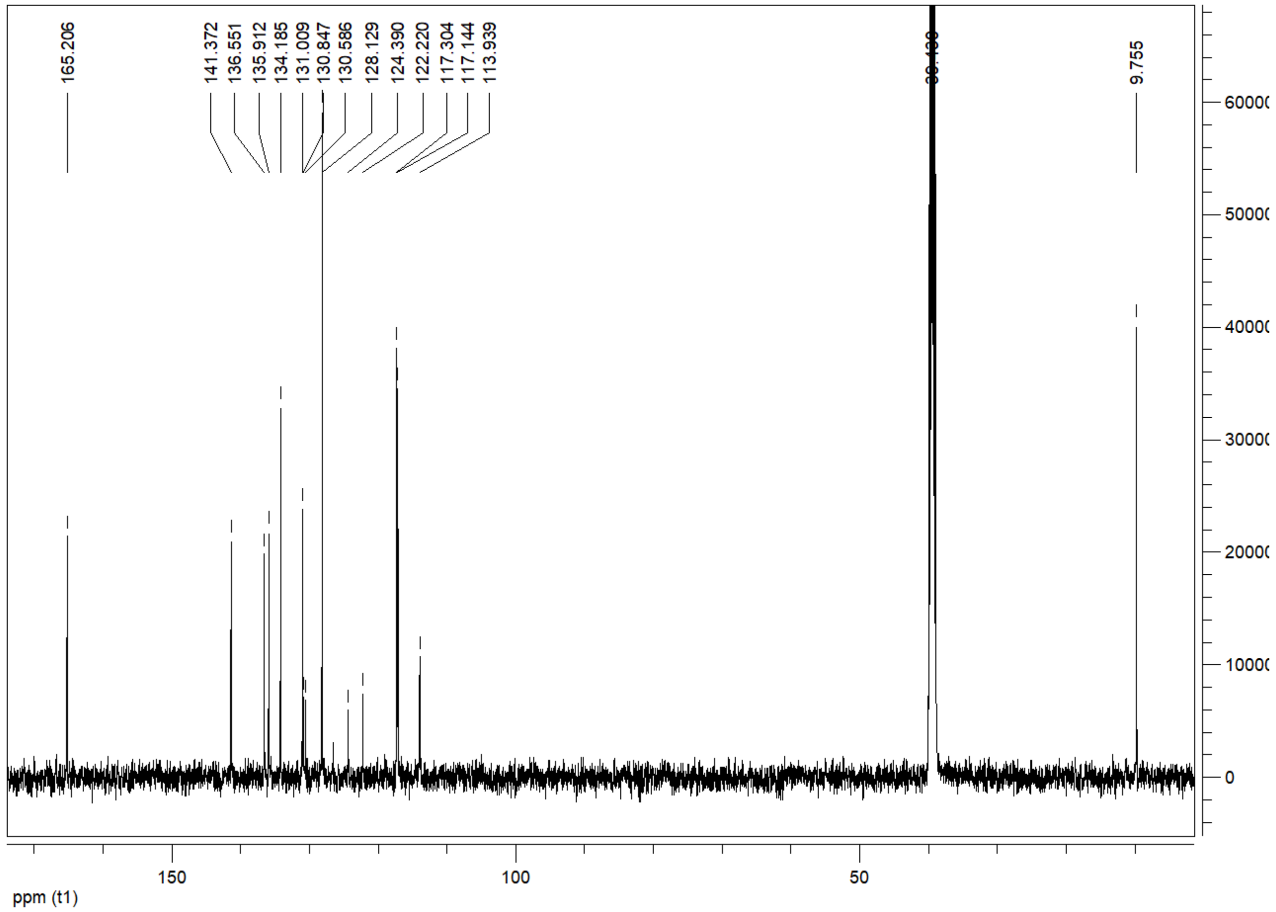

^{1}H, ^{13}C and ^{19}F NMR spectra of compound **23** (DMSO-d6),

N1-(3-(4-methyl-1H-imidazol-1-yl)-5-(trifluoromethyl)phenyl)-N4-(4-((2-(methylcarbamoyl)pyridin-4-yl)oxy)phenyl)terephthalamide

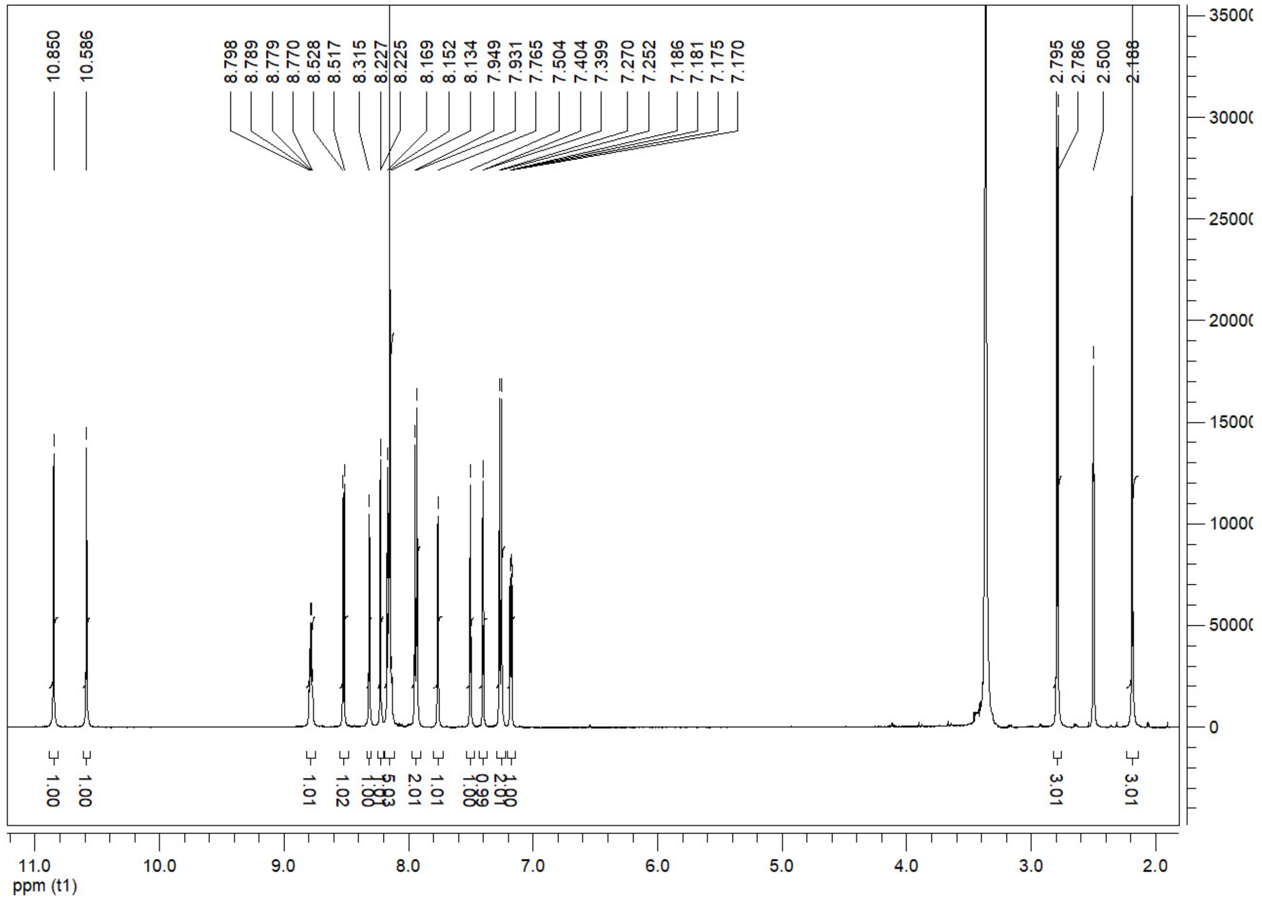

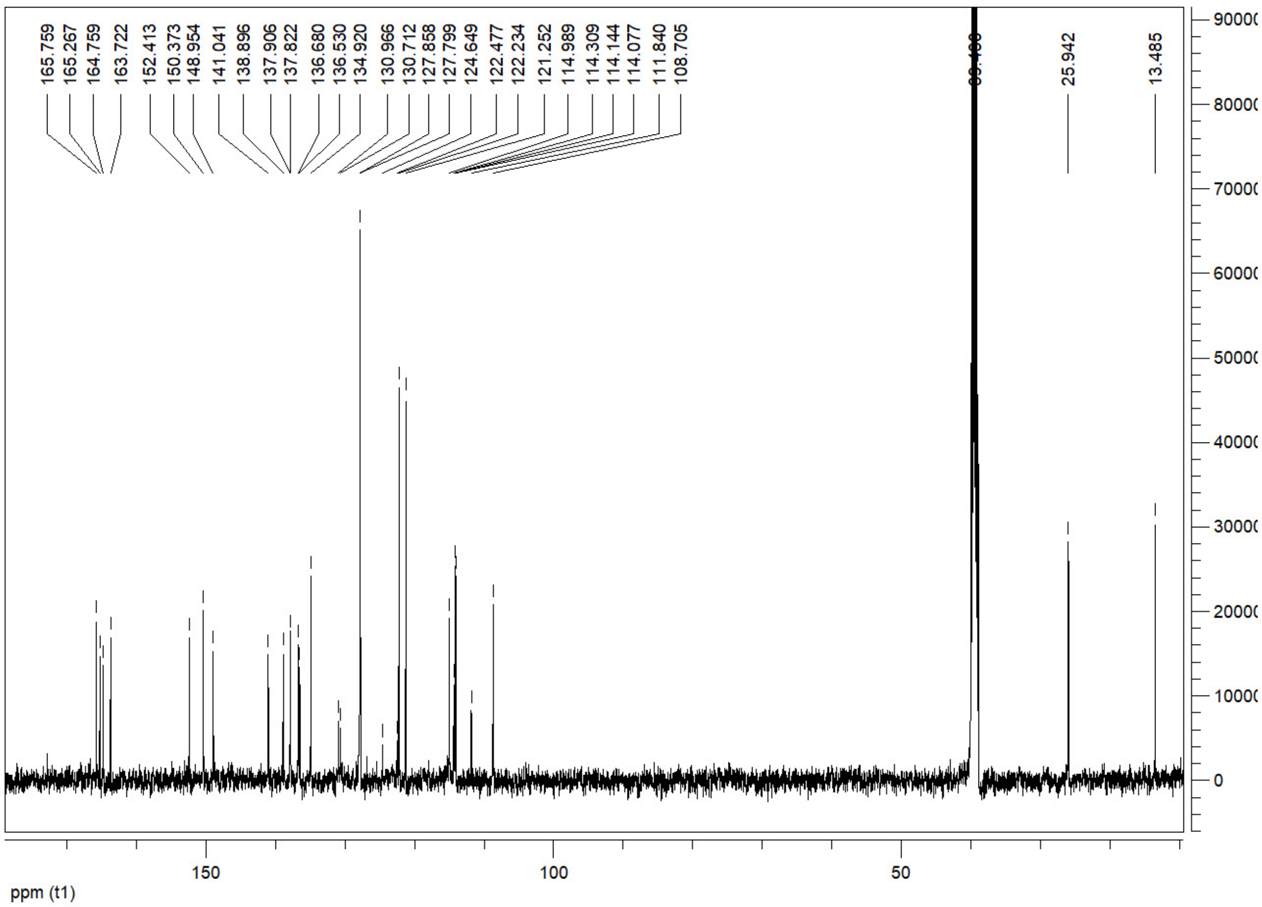
165.759
165.267
164.759
163.722
152.413
150.373
148.954
141.041
138.896
137.906
137.822
136.680
136.530
134.920
130.966
130.712
127.858
127.799
124.649
122.477
122.234
121.252
114.989
114.309
114.144
114.077
111.840
108.705
25.942
13.485
ppm (t1)

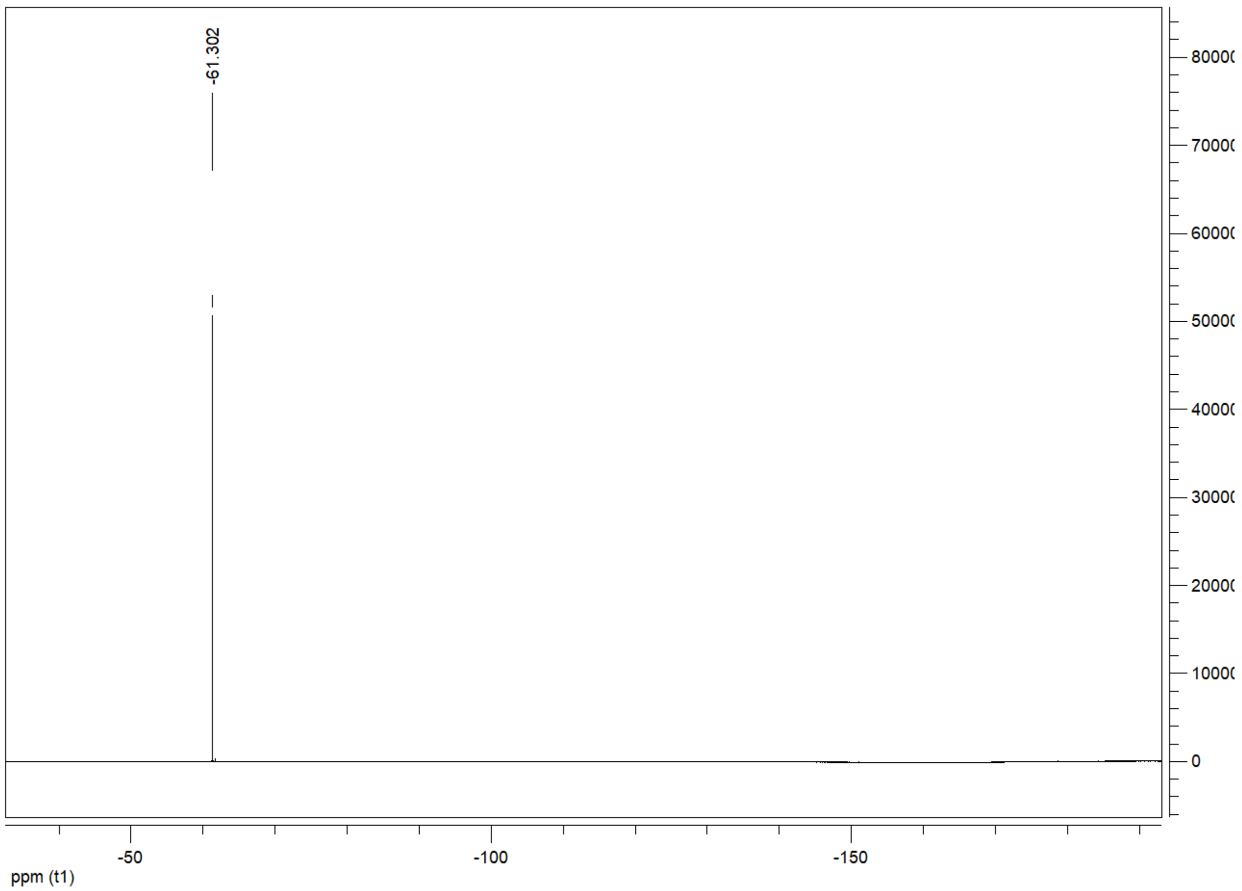
-61.302
ppm (t1)

^{1}H, ^{13}C and ^{19}F NMR spectra of compound **24** (DMSO-d6),

N1-(3-(4-methyl-1H-imidazol-1-yl)-5-(trifluoromethyl)phenyl)-N4-(2-methyl-5-nitrophenyl)terephthalamide

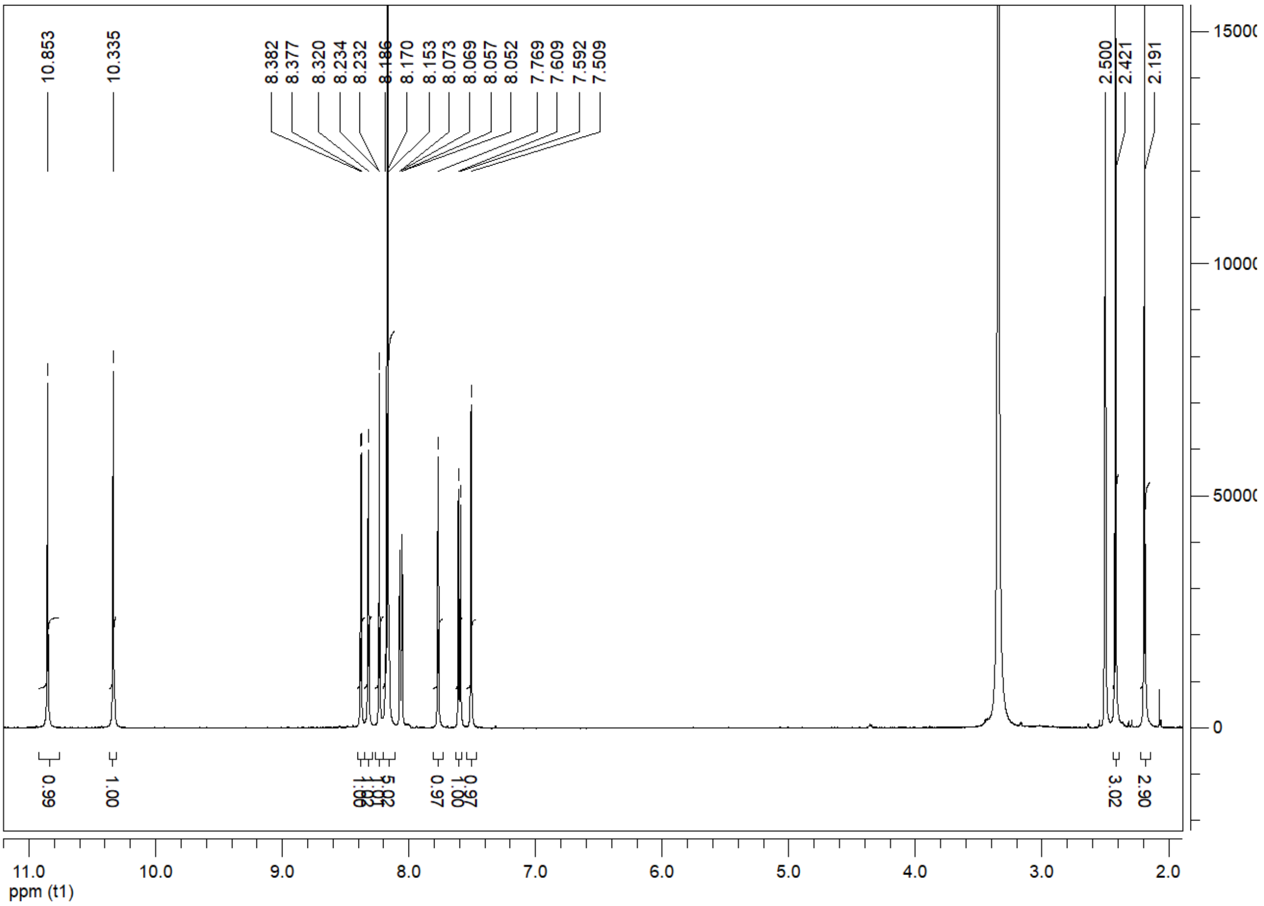

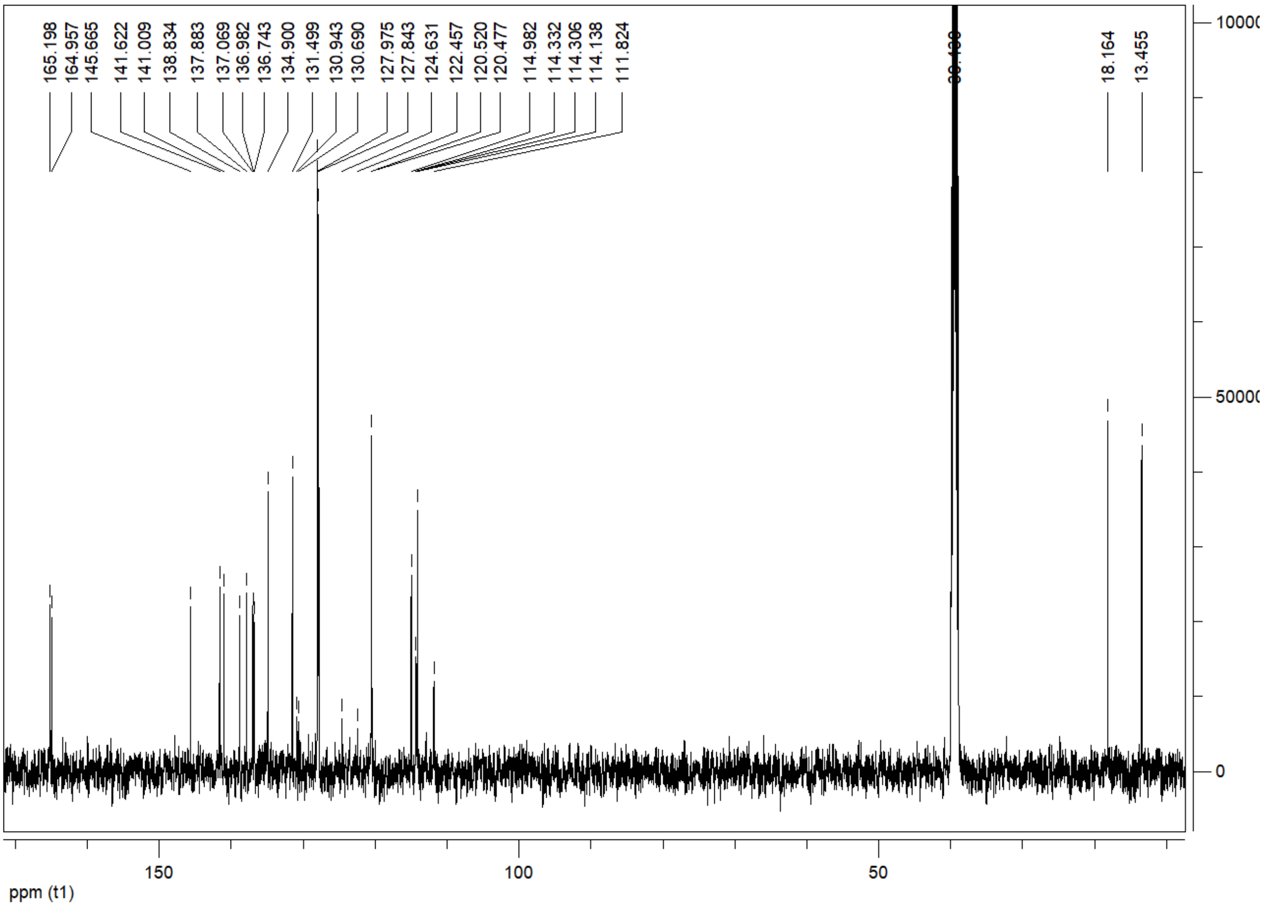

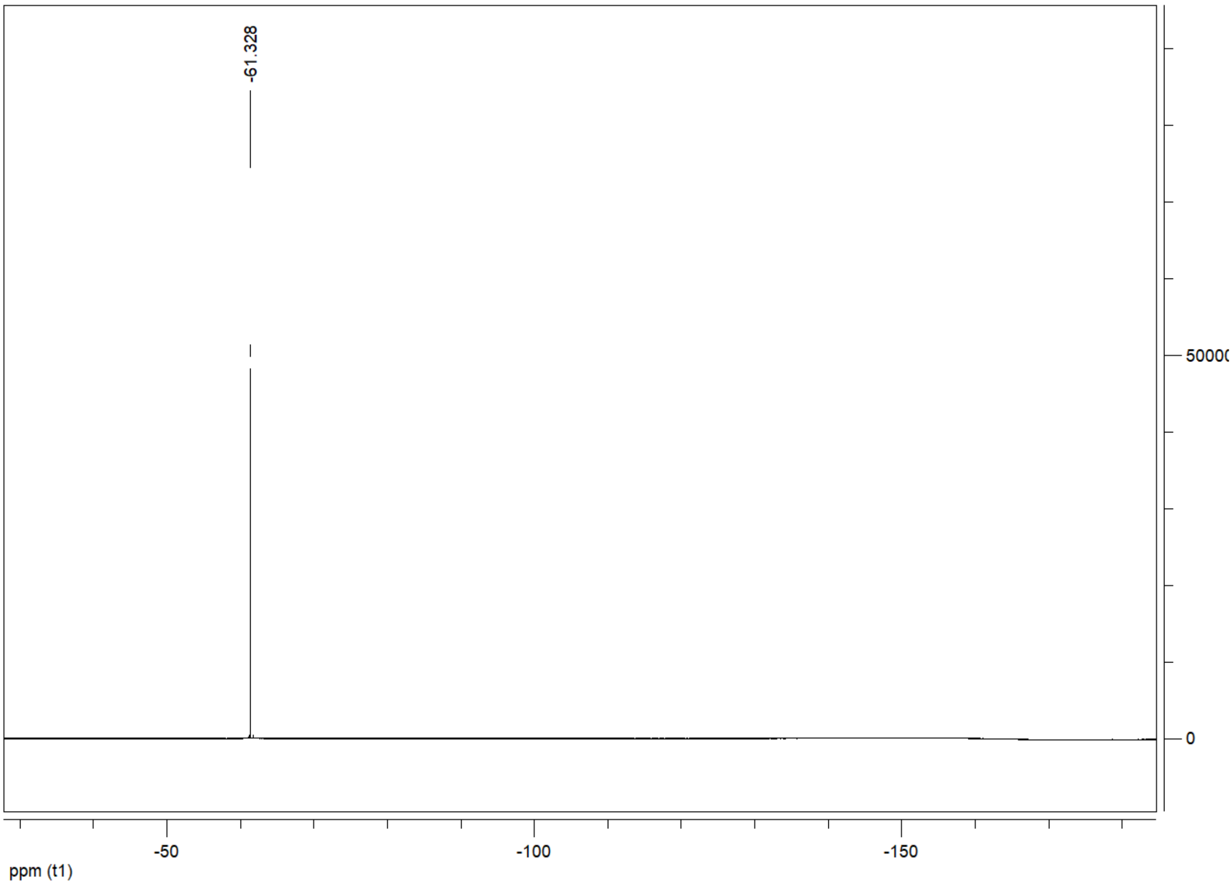

^{1}H, ^{13}C and ^{19}F NMR spectra of compound **25** (DMSO-d6),

4-(4-(4-Methoxybenzoyl)piperazine-1-carbonyl)-N-(3-(4-methyl-1H-imidazol-1-yl)-5-(trifluoromethyl)-phenyl)benzamide

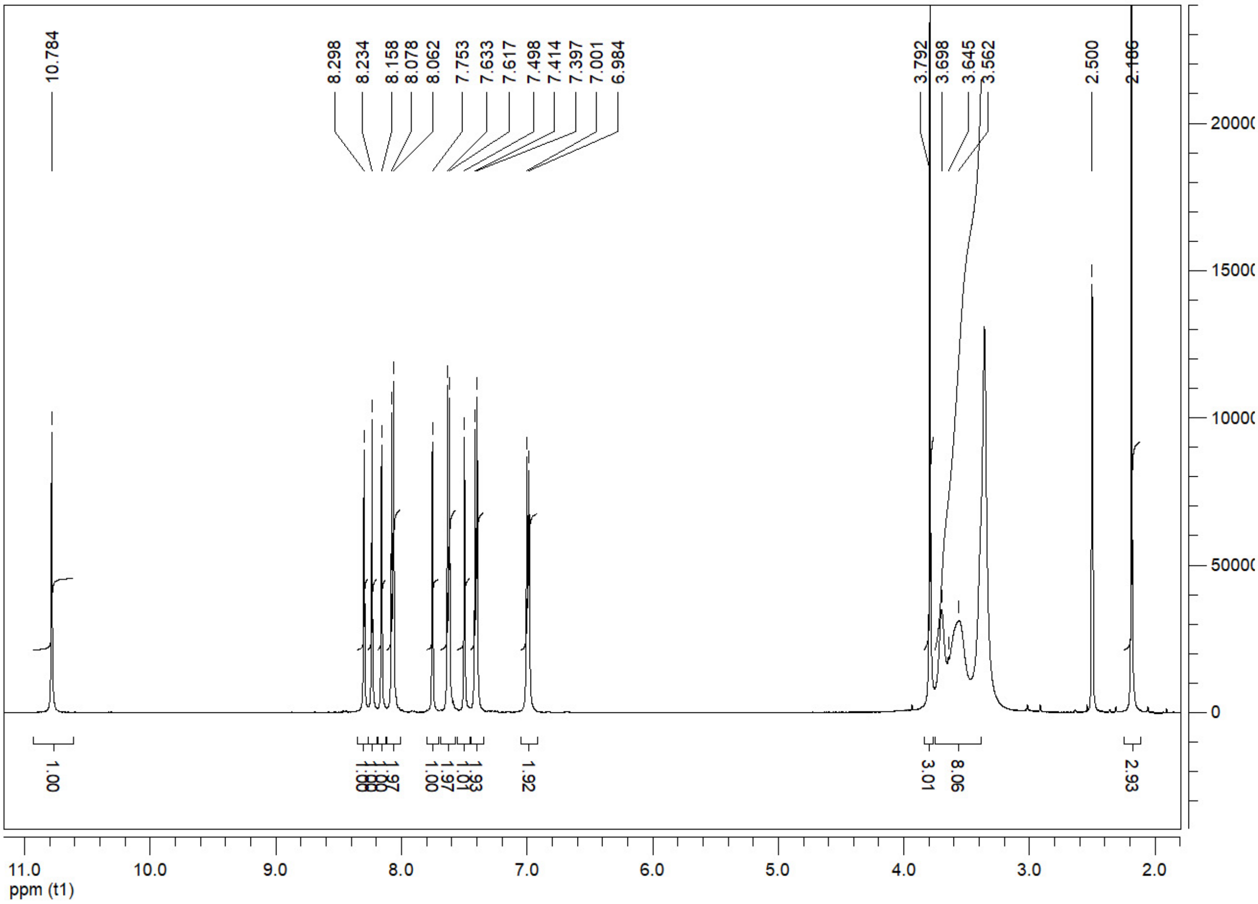

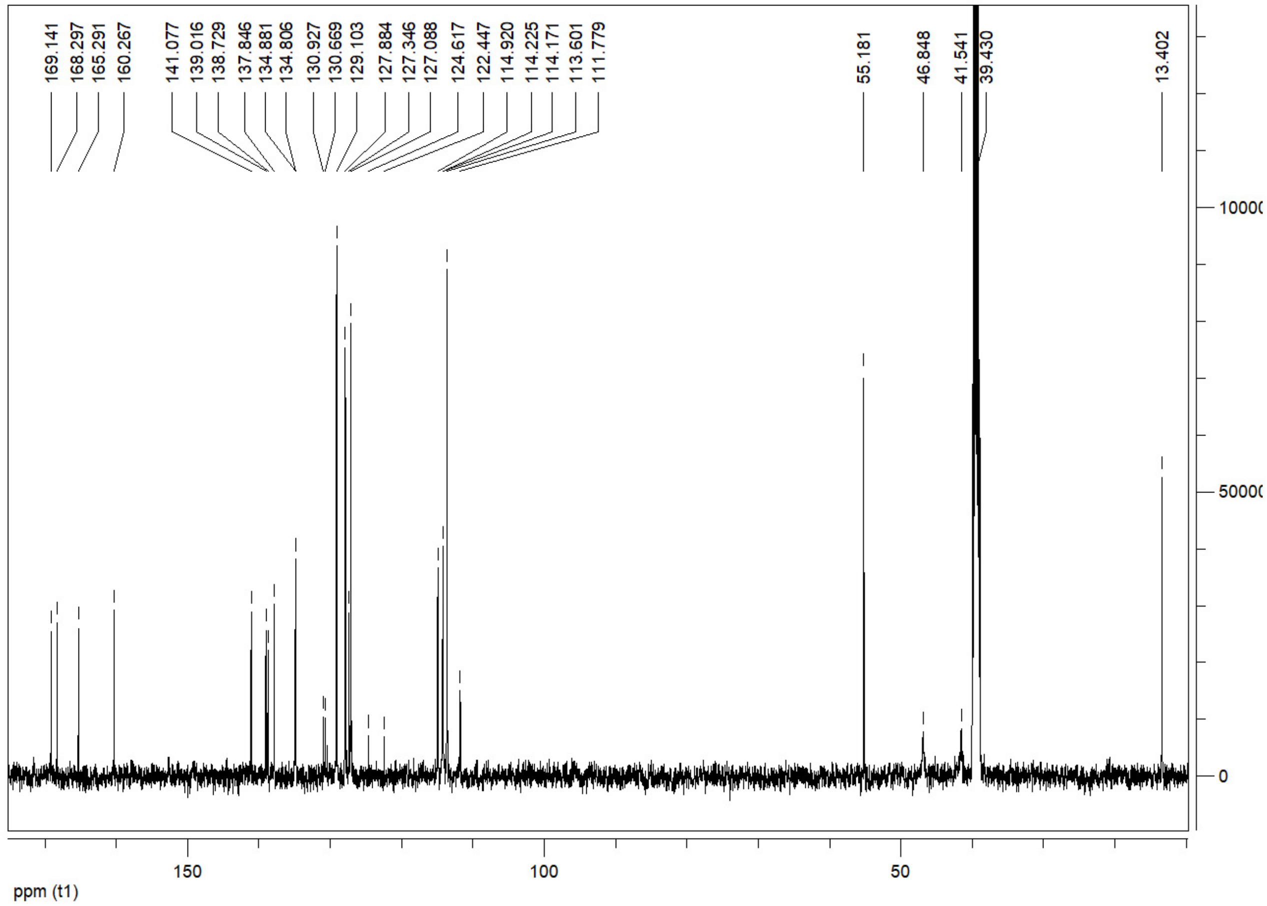
169.141
168.297
165.291
160.267
141.077
139.016
138.729
137.846
134.881
134.806
130.927
130.669
129.103
127.884
127.346
127.088
124.617
122.447
114.920
114.225
114.171
113.601
111.779
55.181
46.848
41.541
39.430
13.402
10000
50000
0
150
100
50
ppm (t1)

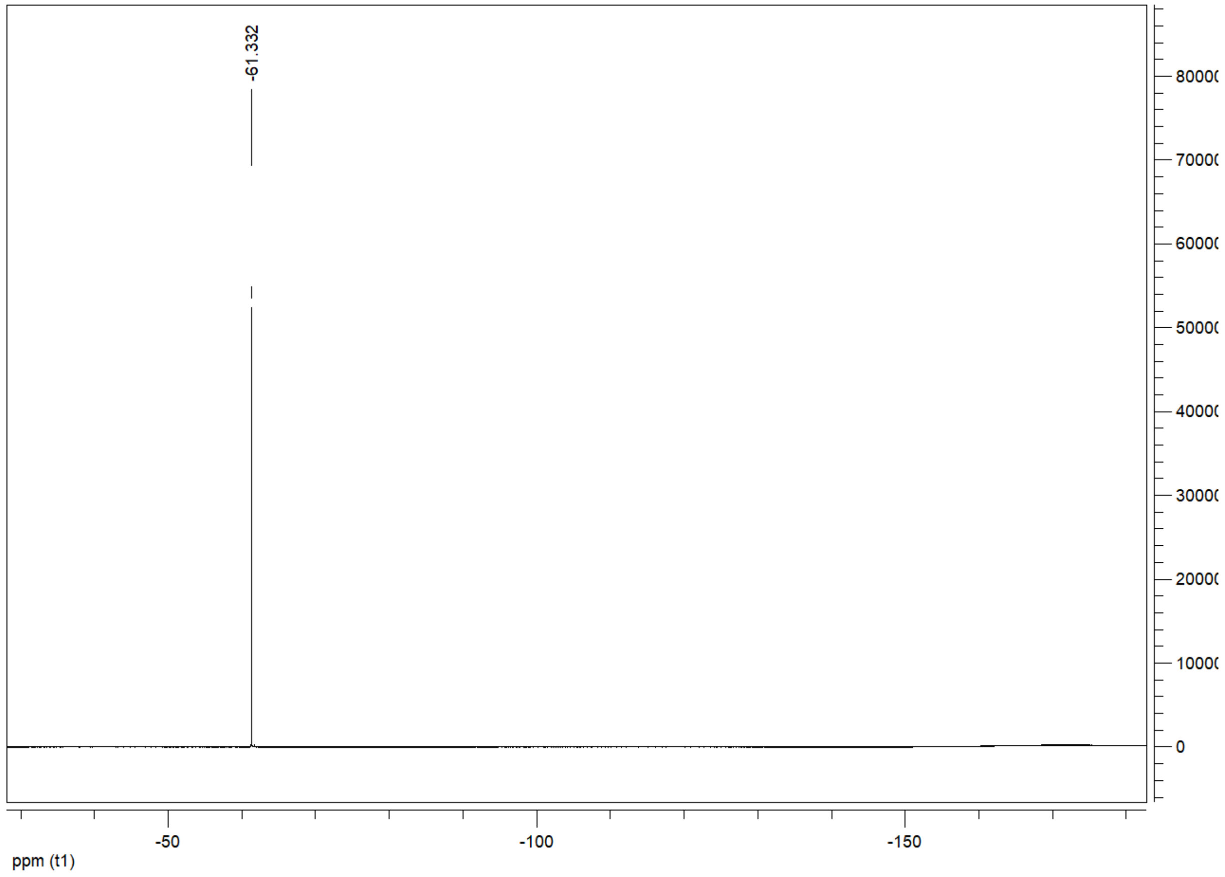
-61.332
80000
70000
60000
50000
40000
30000
20000
10000
0
-50
-100
-150
ppm (t1)

^{1}H, ^{13}C and ^{19}F NMR spectra of compound **26** (DMSO-d6),

4-(4-(2-Fluorobenzoyl)piperazine-1-carbonyl)-N-(3-(4-methyl-1H-imidazol-1-yl)-5-(trifluoromethyl)phenyl)benzamide

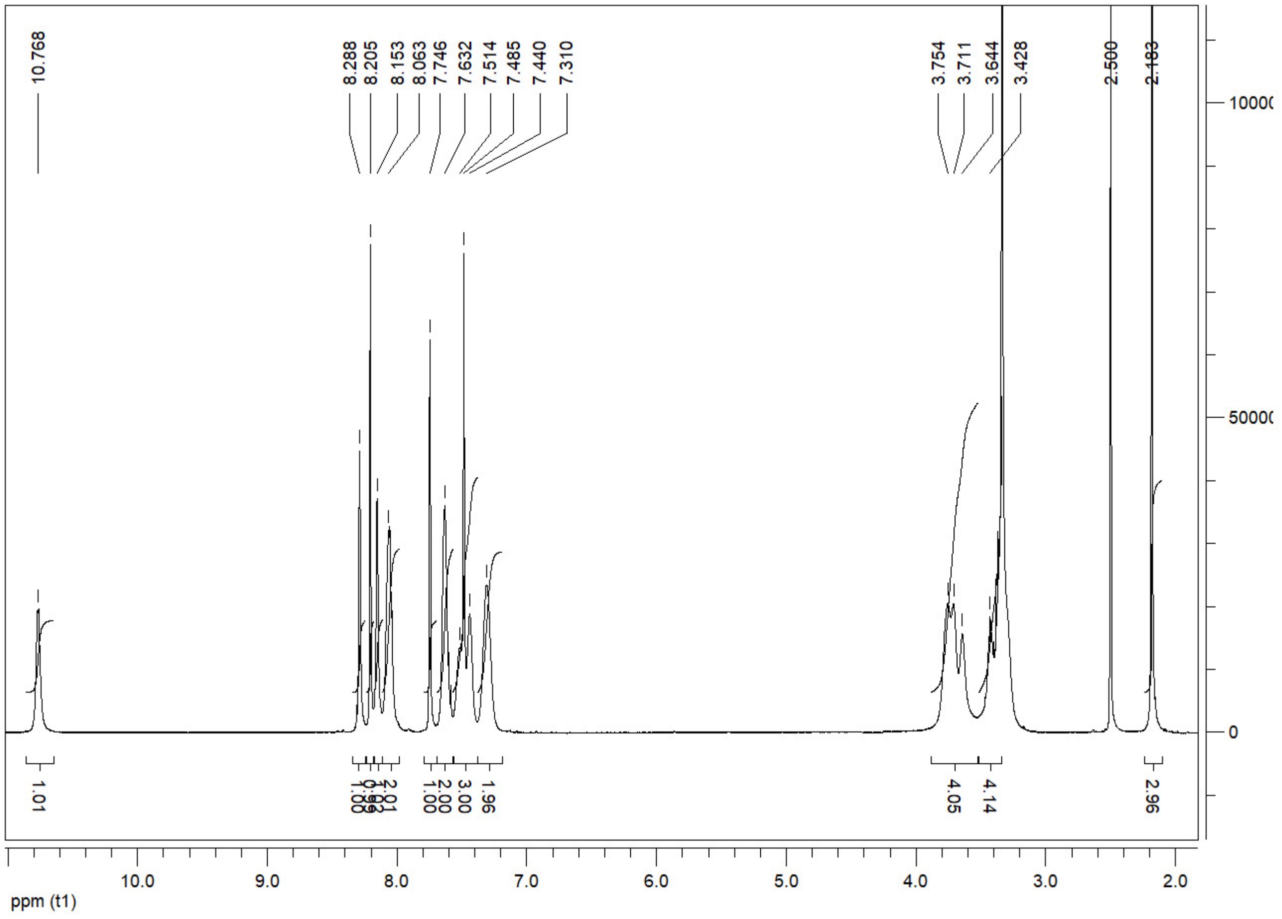

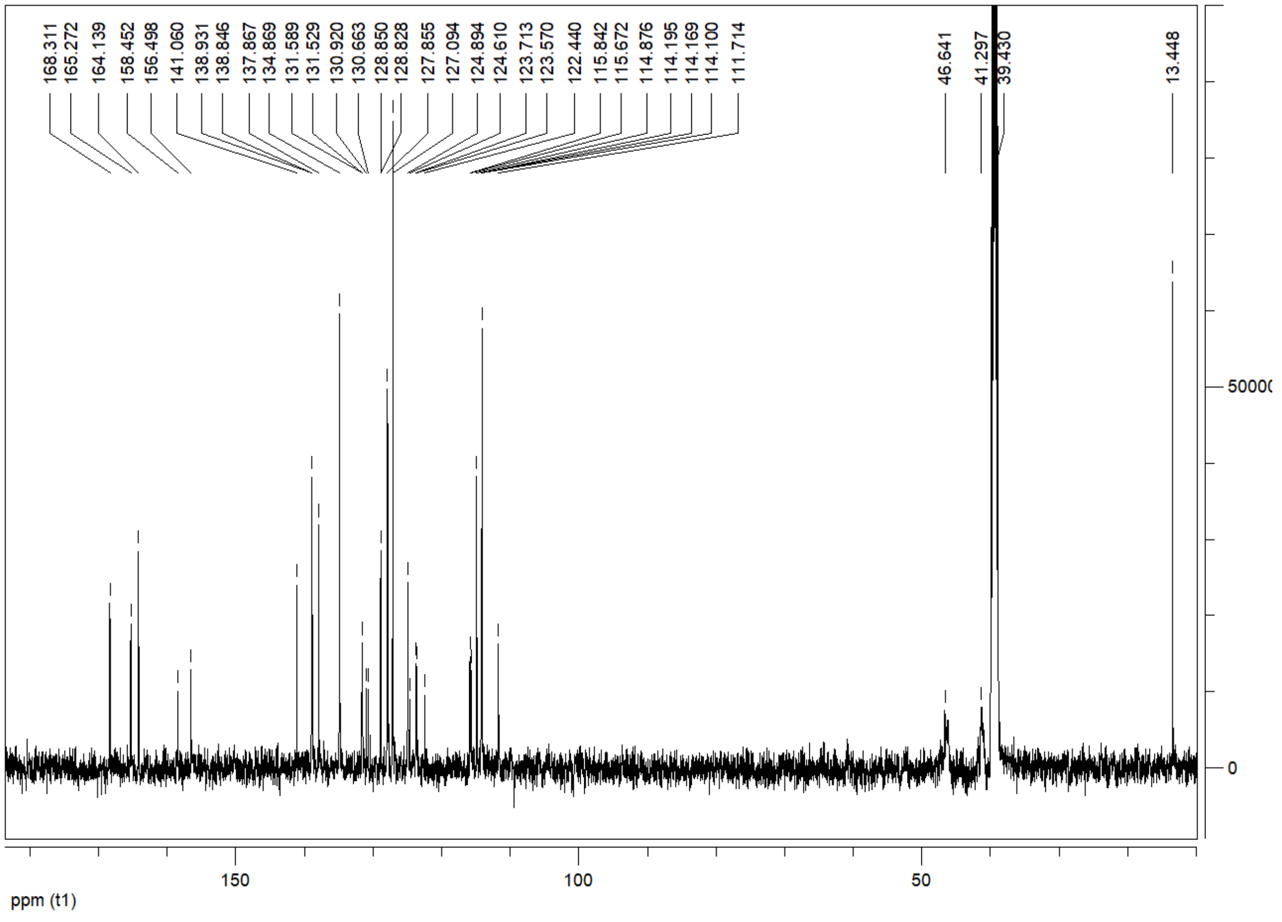

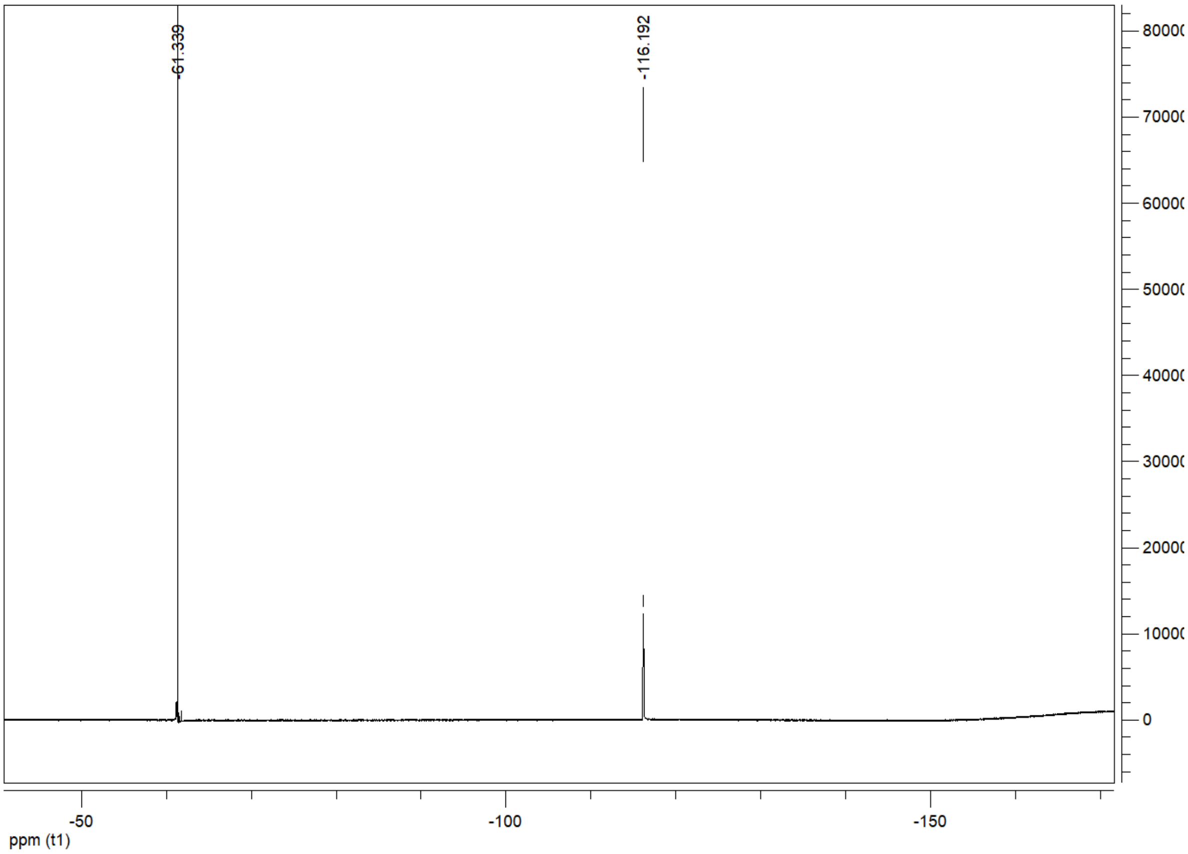

^{1}H, ^{13}C and ^{19}F NMR spectra of compound **27** (DMSO-d6),

4-(4-(3-Fluorobenzoyl)piperazine-1-carbonyl)-N-(3-(4-methyl-1H-imidazol-1-yl)-5-(trifluoromethyl)phenyl)benzamide

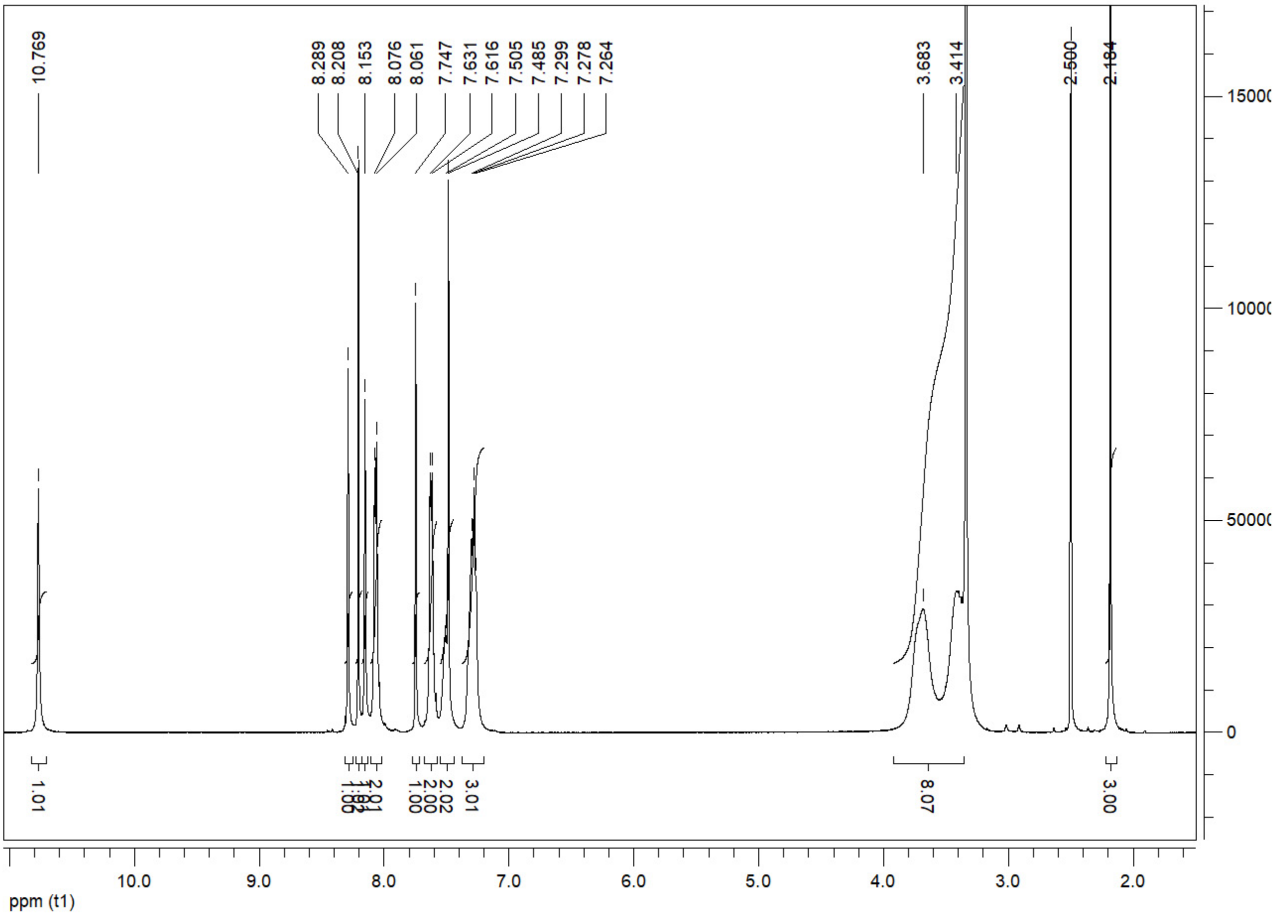

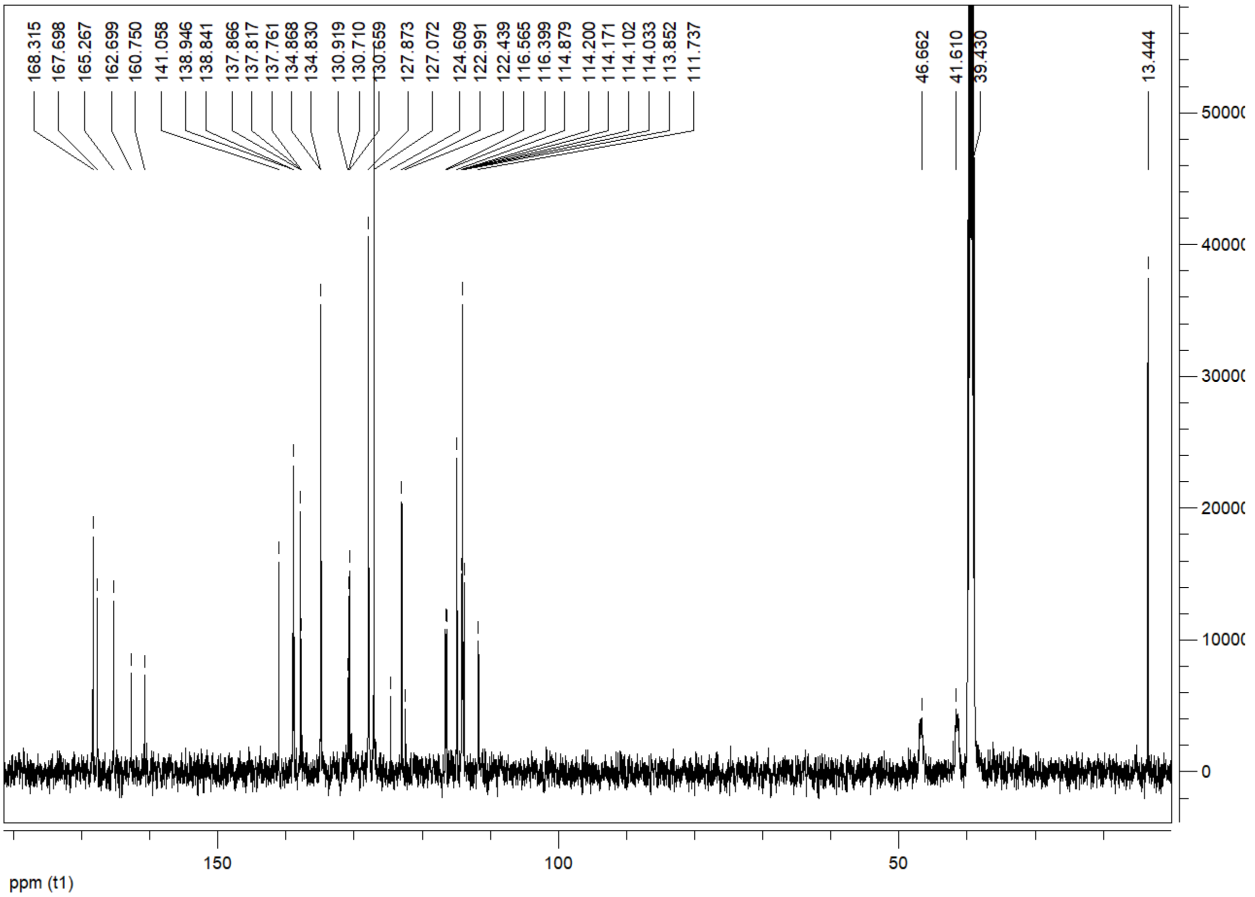
168.315
167.698
165.267
162.699
160.750
141.058
138.946
138.841
137.866
137.817
137.761
134.868
134.830
130.919
130.710
130.659
127.873
127.072
124.609
122.991
122.439
116.565
116.399
114.879
114.200
114.171
114.102
114.033
113.852
111.737
46.662
41.610
39.430
13.444
150
100
50
ppm (t1)

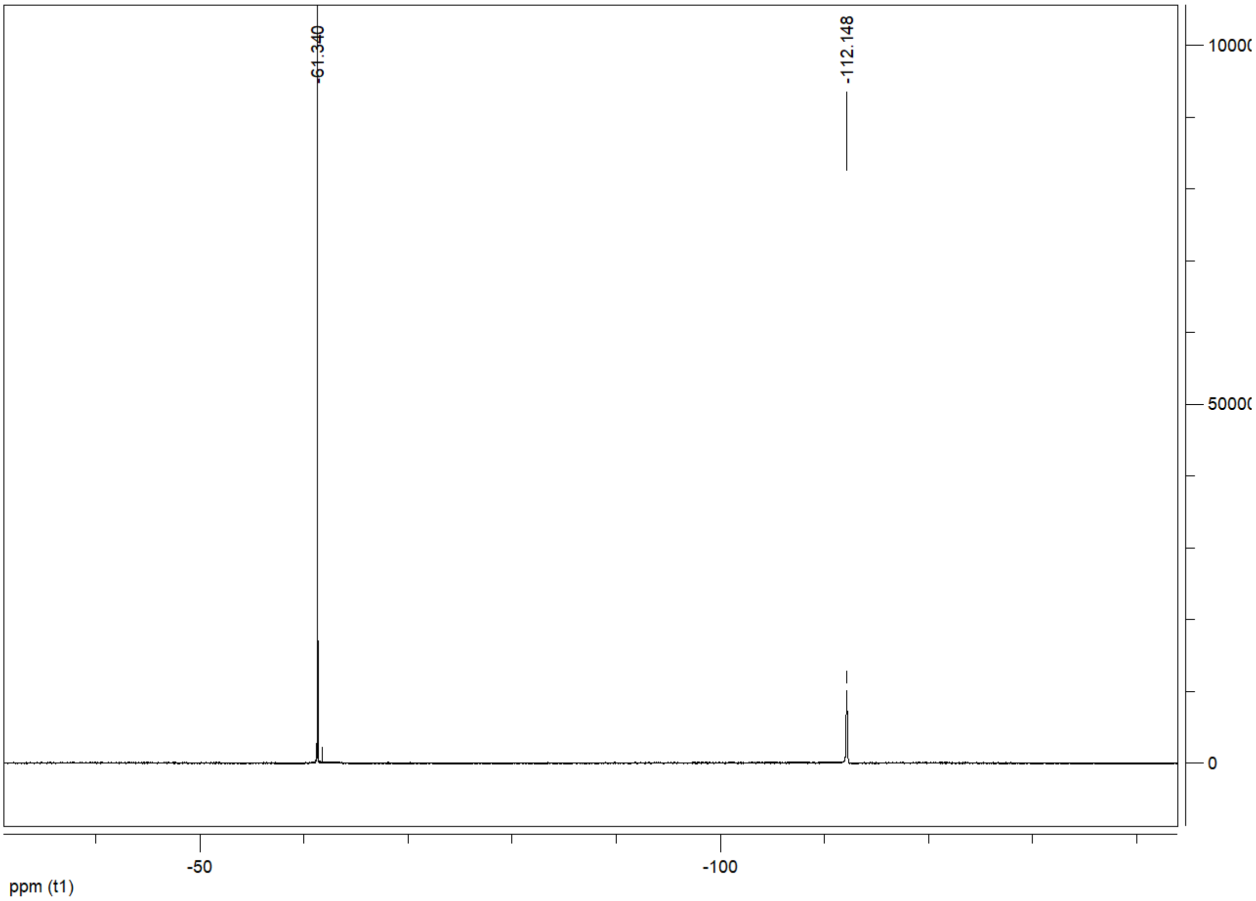
-61.340
-112.148
0
-50
-100
ppm (t1)

^{1}H and ^{13}C NMR spectra of compound **28** (DMSO-d6),

N1-(2-methyl-5-nitrophenyl)-N4-(4-((2-(methylcarbamoyl)pyridin-4-yl)oxy)phenyl)terephthalamide

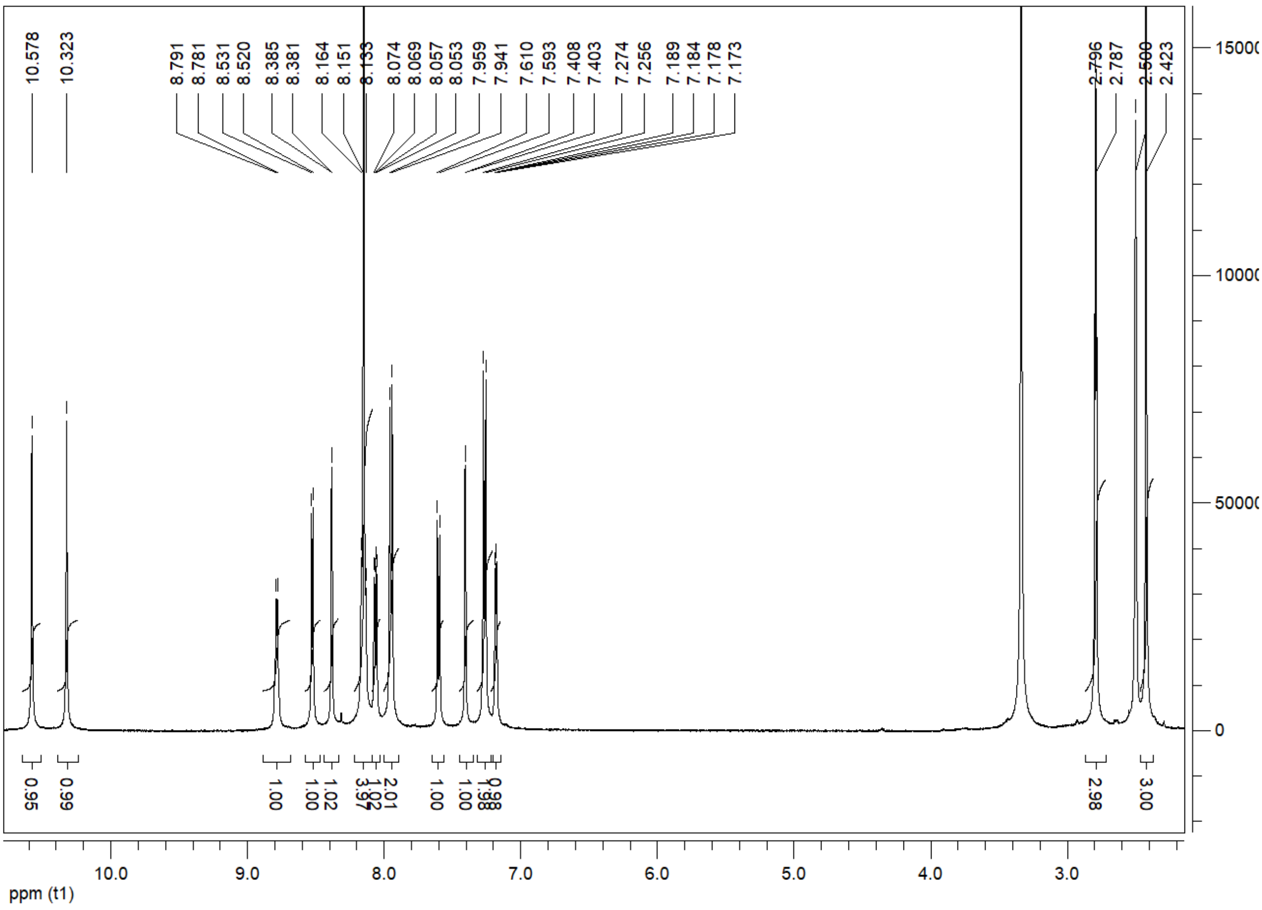

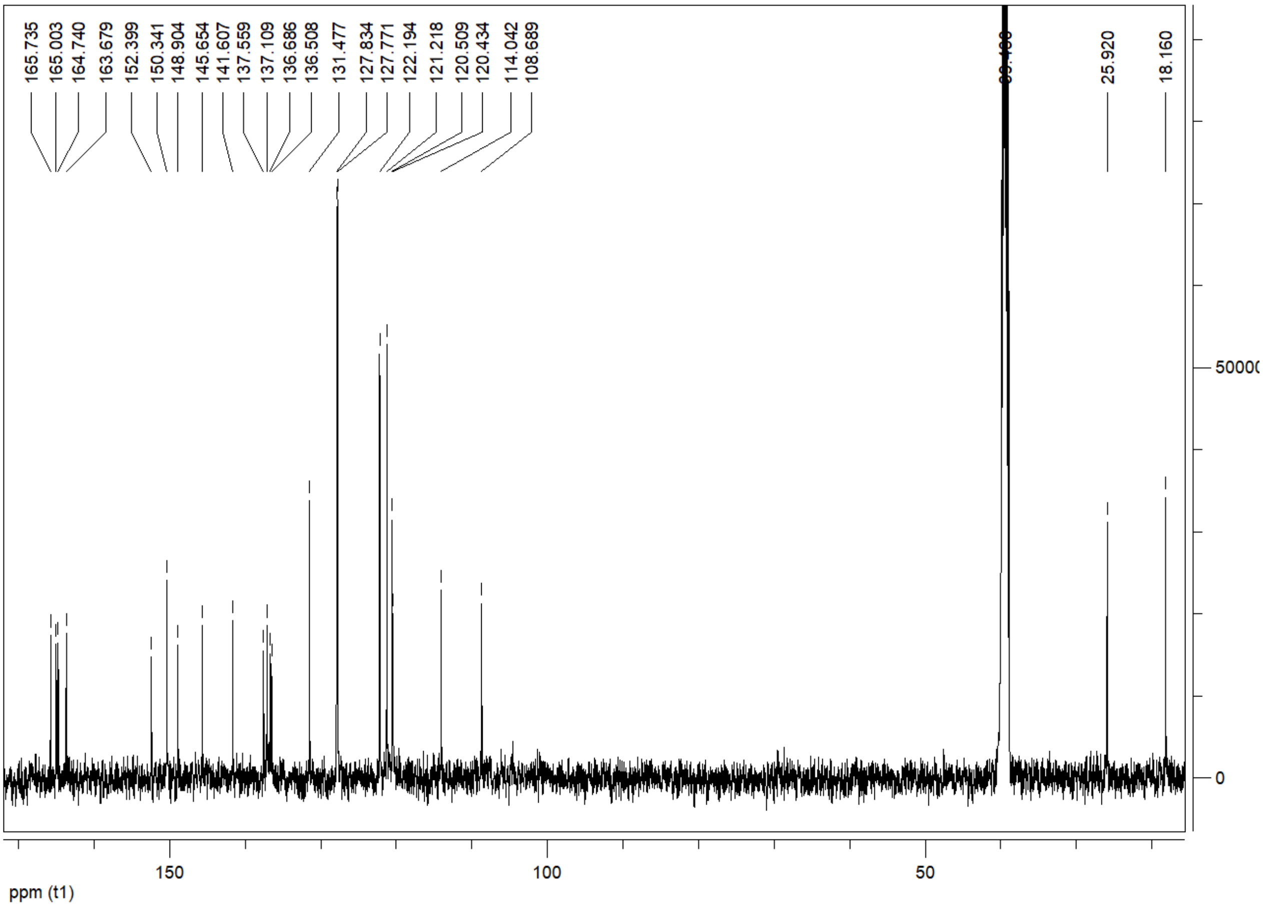

Supplement: Supplementary file 1 [file cimb-45-00117-s001.zip › cimb-2155694-supplementary.pdf]
